# An efficient access to β-ketosulfones *via* β-sulfonylvinylamines: metal–organic framework catalysis for the direct C–S coupling of sodium sulfinates with oxime acetates†

**DOI:** 10.1039/c8ra02389a

**Published:** 2018-05-14

**Authors:** Tuong A. To, Chau B. Tran, Ngoc T. H. Nguyen, Hai H. T. Nguyen, Anh T. Nguyen, Anh N. Q. Phan, Nam T. S. Phan

**Affiliations:** Faculty of Chemical Engineering, HCMC University of Technology, VNU-HCM 268 Ly Thuong Kiet, District 10 Ho Chi Minh City Viet Nam ptsnam@hcmut.edu.vn (+84 8) 38637504 (+84 8) 38647256 ext. 5681

## Abstract

A copper-based framework Cu_2_(OBA)_2_(BPY) was synthesized and used as a recyclable heterogeneous catalyst for the synthesis of β-sulfonylvinylamines from sodium sulfinates and oxime acetates *via* direct C–S coupling reaction. The transformation was remarkably affected by the solvent, and chlorobenzene emerged as the best option. This Cu-MOF displayed higher activity than numerous conventional homogeneous and MOF-based catalysts. The catalyst was reutilized many times in the synthesis of β-sulfonylvinylamines without considerably deteriorating in catalytic efficiency. These β-sulfonylvinylamines were readily converted to the corresponding β-ketosulfones *via* a hydrolysis step with aqueous HCl solution. To the best of our knowledge, this direct C–S coupling reaction to achieve β-sulfonylvinylamines was not previously conducted with a heterogeneous catalyst.

## Abstract 2

Cu_2_(OBA)_2_(BPY) was used as catalyst for the synthesis of β-sulfonylvinylamines from sodium sulfinates and oxime acetates. These β-sulfonylvinylamines were readily converted to corresponding β-ketosulfones *via* a hydrolysis step.

## Graphical abstract

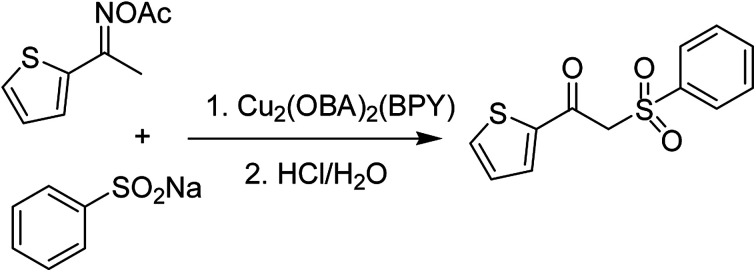

## 1. Introduction

Sulfone derivatives have emerged as essential intermediates for the synthesis of numerous compounds with potentially valuable biological activities.^1–4^ Diverse synthetic protocols have been developed for the construction of these skeletons owing to their significance. Tang *et al.* previously reported the synthesis of β-ketosulfones *via* a tetrabutylammonium iodide-mediated oxidative coupling reaction of sulfonylhydrazides with enamides.^5^ Kumar and Muthyala employed 1-butyl-3-methylimidazolium *p*-toluenesulfinate as a reagent to afford the corresponding β-ketosulfones.^6^ Loghmani-Khouzani and Hajiheidari prepared β-ketosulfones by the reaction of thiols with α-bromoacetophenones, subsequently followed by an oxidation step.^7^ Yang *et al.* demonstrated an efficient protocol to achieve β-ketosulfones *via* a visible-light initiated direct oxysulfonylation between alkenes and sulfinic acids under transition-metal-free conditions.^8^ Xie *et al.* synthesized β-ketosulfones based on a photoinduced radical fragmentation and rearrangement of vinyl tosylates.^9^ Recently, Tang *et al.* reported for the first time the Cu(OAc)_2_-catalyzed synthesis of β-sulfonylvinylamines from oxime acetates and sodium sulfinates, and upon hydrolysis, corresponding β-ketosulfones were readily achieved in high yields.^10^ To establish greener pathways to β-ketosulfones, heterogeneous catalysts should be utilized for the formation of β-sulfonylvinylamines to achieve easy catalyst recovery and recycling, as well as facile product isolation and purification.

Metal–organic frameworks (MOFs) are crystalline and self-organizing reticular materials, consisting of metal cations sharing polytopic organic linkers.^11–15^ Applications of MOFs in the field of catalysis are somewhat delaying behind other domains.^16,17^ With remarkably high porosity and almost infinite synthetic tunability, MOFs have exhibited several advantages in catalysis over non-porous and zeolitic materials.^18,19^ Generally, catalytically active sites on MOFs could originate from the metal-based building units or from functional groups on the organic linkers.^20–22^ The isolation of metal points in the networks would provide a greater number of active sites for the reactants.^23,24^ Many organic reactions conducted with MOFs-based catalysts have been declared in the literature.^20,25–28^ Nevertheless, β-sulfonylvinylamines and β-ketosulfones were not previously synthesized utilizing heterogeneous catalysts, and the exploration of recyclable catalysts should be necessary for the construction of these skeletons. In this manuscript, we would like to illustrate an efficient access to β-sulfonylvinylamines from sodium sulfinates and oxime acetates *via* direct C–S coupling reaction utilizing Cu_2_(OBA)_2_(BPY) metal–organic framework catalysis. The Cu-MOF catalyst was reutilized many times without a noticeably deterioration in catalytic efficiency. These β-sulfonylvinylamines were readily converted to β-ketosulfones with a hydrolysis step with aqueous HCl solution.

## 2. Experimental

### 2.1. Synthesis of metal–organic framework Cu_2_(OBA)_2_(BPY)

In a typical procedure, 4,4′-oxybis(benzoic) acid (H_2_OBA) (0.258 g, 1 mmol), copper(ii) nitrate trihydrate (Cu(NO_3_)_2_·3H_2_O) (0.242 g, 1 mmol), and 4,4′-bipyridine (BPY) (0.078 g, 0.5 mmol) were dissolved in the mixture of DMF and distilled water (14 mL, 11 : 3 v/v). The mixture was magnetically stirred for 30 min to obtain a clear solution. The solution was subsequently distributed to three 10 mL vials. The vials were carefully capped and heated at 85 °C in an isothermal oven for 48 h. Green crystals were formed during the experiment. After the vials were cooled to ambient temperature, the solid product in each vial was collected by decantation, and washed in DMF (3 × 10 mL). Solvent exchange was consequently performed with methanol (3 × 10 mL) at room temperature. The framework was then dried at 150 °C for 6 h under vacuum on a Shlenkline to have 0.29 g of Cu_2_(OBA)_2_(BPY) in the form of green crystals (75% yield regarding H_2_OBA).

### 2.2. Catalytic studies

In a typical catalytic experiment, a mixture of 1-(thiophen-2-yl)ethanone *O*-acetyl oxime (0.25 mmol, 45.8 mg), sodium benzenesulfinate (0.3 mmol, 49.2 mg), Cu_2_(OBA)_2_(BPY) (10 mol%) in chlorobenzene (1 mL) was added to a 10 mL screw-cap vial with magnetic stirrer bar. The catalyst amount was calculated regarding the copper/1-(thiophen-2-yl)ethanone *O*-acetyl oxime molar ratio. The mixture was stirred at 100 °C for 3 h under an argon atmosphere. After the reaction was complete, aliquots were withdrawn from the reaction mixture, quenched with brine (1 mL), extracted with ethyl acetate (3 × 1 mL), and dried over anhydrous Na_2_SO_4_. Samples were then analyzed by GC concerning *n*-dodecane internal standard, giving GC yield of (*Z*)-2-(phenylsulfonyl)-1-(thiophen-2-yl)ethenamine. Ethyl acetate was removed under vacuum, and the crude product was purified by recrystallization in chlorobenzene and hexane.

In order to achieve 2-(phenylsulfonyl)-1-(thiophen-2-yl)ethanone, after the first step, the reaction mixture was cooled to room temperature and filtered to remove the Cu_2_(OBA)_2_(BPY) catalyst. The filtrate was then magnetically stirred with aqueous HCl solution (1 M, 1 mL) at 80 °C for 3 h. The resulting mixture was quenched with brine (5 mL), and the organic ingredients were extracted into ethyl acetate (3 × 5 mL). The combined ethyl acetate solution was dried over anhydrous Na_2_SO_4_. The solvent was subsequently removed under vacuum, and the crude product was purified by silica gel column chromatography utilizing hexane and ethyl acetate (3 : 1, v/v) as eluent to obtain the expected β-ketosulfone. Product structure was subsequently confirmed by GC-MS, ^1^H NMR, and ^13^C NMR.

## 3. Results and discussion

**Scheme 1:**
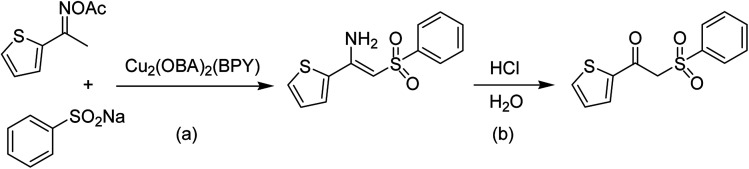
The direct C–S coupling reaction utilizing Cu_2_(OBA)_2_(BPY) catalyst (a), and the hydrolysis step to form β-ketosulfone (b).

**Fig. 1:**
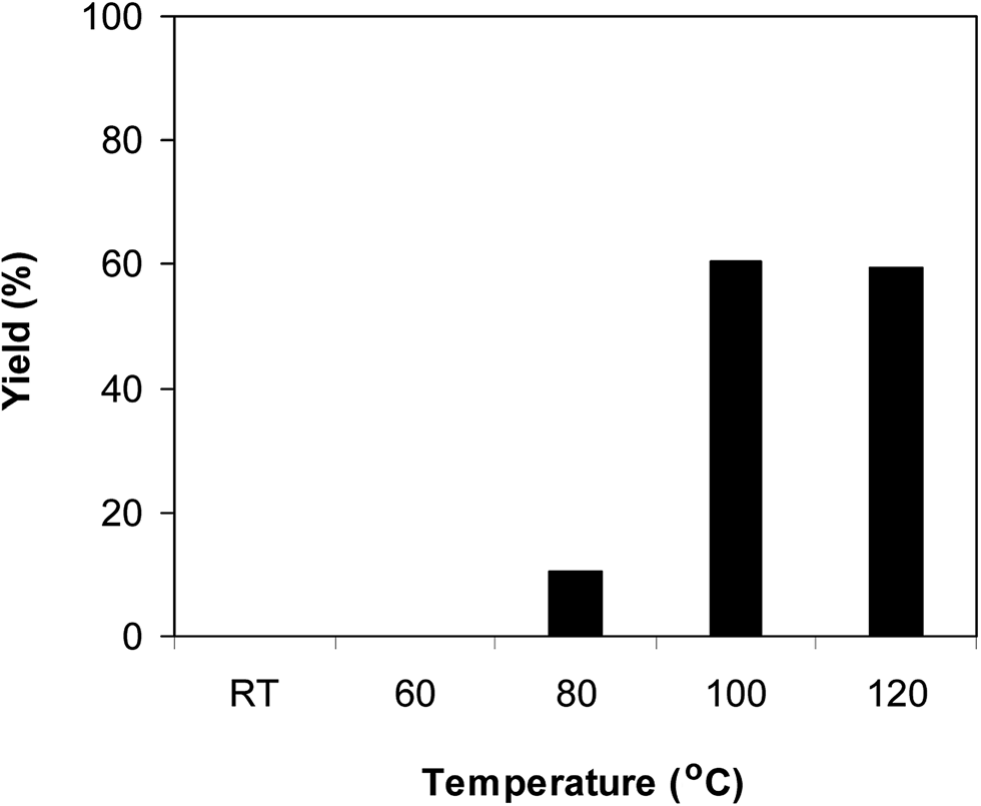
Yield of (*Z*)-2-(phenylsulfonyl)-1-(thiophen-2-yl)ethenamine *vs.* temperature.

**Fig. 2:**
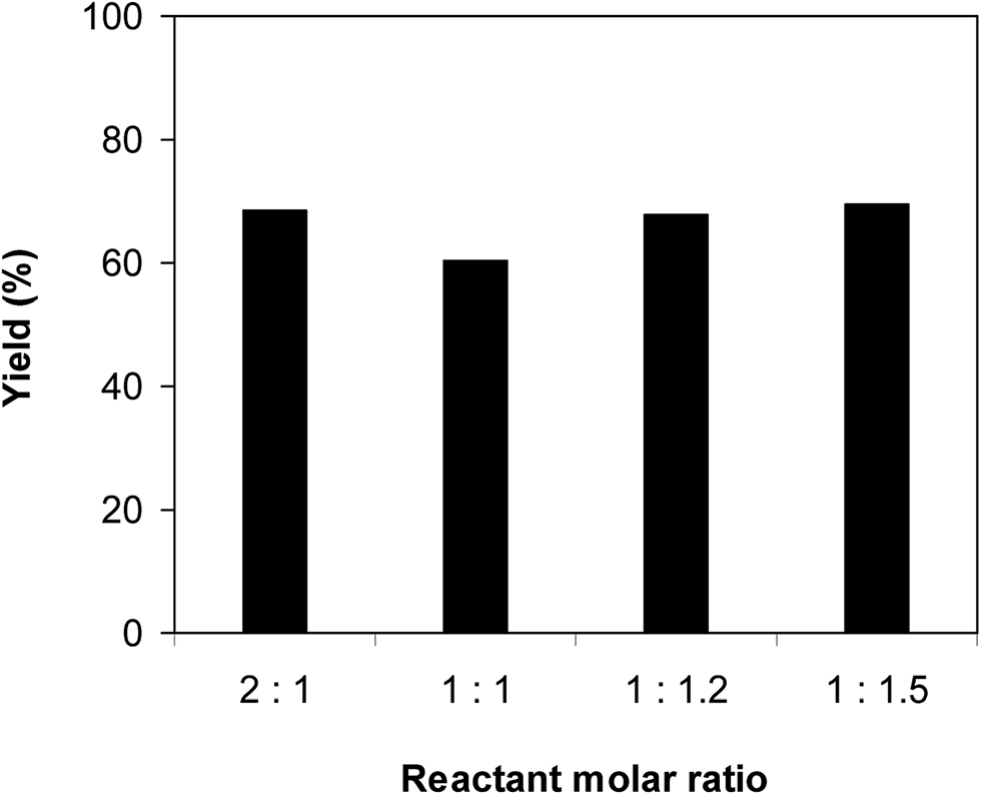
Yield of (*Z*)-2-(phenylsulfonyl)-1-(thiophen-2-yl)ethenamine *vs.* reactant molar ratio.

The Cu_2_(OBA)_2_(BPY) was synthesized in 75% yield from 4,4′-oxybis(benzoic) acid, copper(ii) nitrate trihydrate, and 4,4′-bipyridine following a literature approach.^29,30^ The copper–organic framework was consequently characterized by utilizing conventional analysis methods (Fig. S1–S7†). The Cu-MOF was initially used as a heterogeneous catalyst for the direct C–S coupling reaction between 1-(thiophen-2-yl)ethanone *O*-acetyl oxime and sodium benzenesulfinate to produce (*Z*)-2-(phenylsulfonyl)-1-(thiophen-2-yl)ethenamine (Scheme 1a). First, the influence of temperature on the reaction yield was explored. The reaction was performed in toluene under argon for 3 h, at 10 mol% catalyst, with reactant molar ratio of 1 : 1 and reactant concentration of 0.25 M, at room temperature, 60 °C, 80 °C, 100 °C, and 120 °C, respectively. The reaction did not proceed at room temperature and 60 °C with no trace quantity of product being noted. Increasing the temperature to 80 °C resulted in only 10% yield. The reaction yield was remarkably improved to 60% for the reaction conducted at 100 °C. Boosting the temperature to 120 °C did not led to higher yield of the desired product (Fig. 1). It was noted that the yield of the expected product was improved by changing the reactant molar ratio, having investigated the reaction with 1-(thiophen-2-yl)ethanone *O*-acetyl oxime : sodium benzenesulfinate molar ratio of 2 : 1, 1 : 1, 1 : 1.2, and 1 : 1.5, respectively. The reaction utilizing two equivalents of 1-(thiophen-2-yl)ethanone *O*-acetyl oxime afforded 69% yield. Similar yield was achieved by employing 1.2 equivalents of sodium benzenesulfinate. Increasing the amount of sodium benzenesulfinate to 1.5 equivalents was not necessary as the yield of (*Z*)-2-(phenylsulfonyl)-1-(thiophen-2-yl)ethenamine was not enhanced noticeably (Fig. 2).

**Fig. 3:**
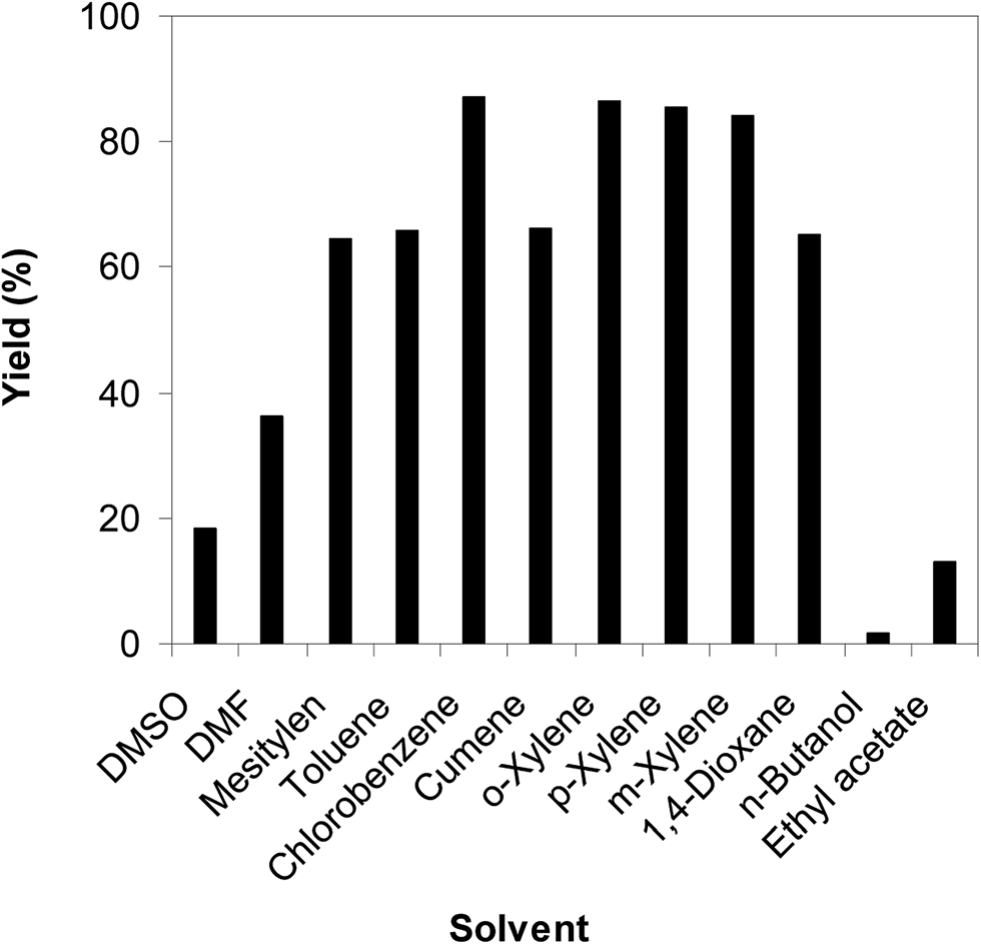
Yield of (*Z*)-2-(phenylsulfonyl)-1-(thiophen-2-yl)ethenamine *vs.* solvent.

As the direct C–S coupling reaction using the copper-based framework catalyst proceeded in solution, changing the solvent could led to higher yield. Tang *et al.* previously reported for the first time the Cu(OAc)_2_-catalyzed synthesis of β-sulfonylvinylamines from oxime acetates and sodium sulfinates, and pointed out that toluene was the best solvent for this transformation.^10^ We consequently studied the impact of different solvents on the yield of (*Z*)-2-(phenylsulfonyl)-1-(thiophen-2-yl)ethenamine. The reaction was conducted at 100 °C in each solvent under argon for 3 h, in the presence of 10 mol% catalyst, with 1.2 equivalents of sodium benzenesulfinate and at reactant concentration of 0.25 M. DMSO and DMF were not appropriate for the reaction, producing the β-sulfonylvinylamine product in 18% and 36% yields, respectively. Changing the solvent to mesitylen, 64% yield was observed, while 68% and 66% yields were detected to the case of toluene and cumene, respectively. The yield was upgraded to 86% for the reaction carried out in *o*-xylene. Similarly, 85% and 84% yields were obtained for the reaction in *p*-xylene and *m*-xylene, respectively. Moving to 1,4-dioxane, the yield was decreased to 65% yield. *n*-Butanol and ethyl acetate should not be used for this reaction. Compared to these solvents, chlorobenzene emerged as the solvent of choice, affording 87% yield of (*Z*)-2-(phenylsulfonyl)-1-(thiophen-2-yl)ethenamine (Fig. 3).

**Fig. 4:**
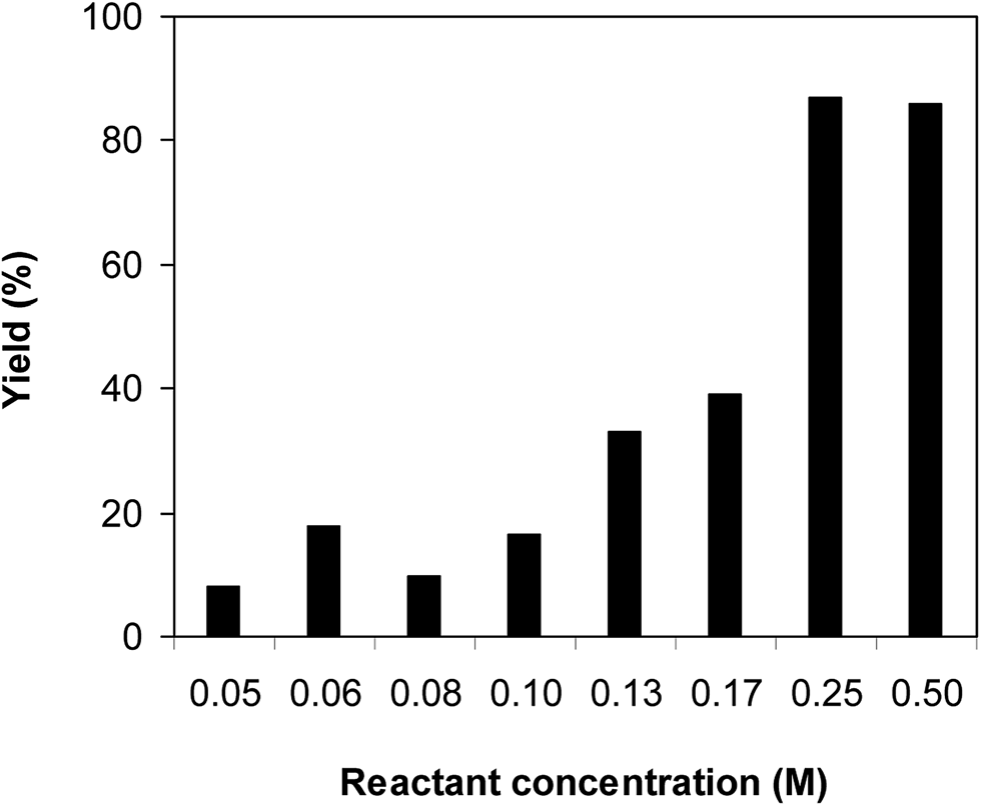
Yield of (*Z*)-2-(phenylsulfonyl)-1-(thiophen-2-yl)ethenamine *vs.* reactant concentration.

**Fig. 5:**
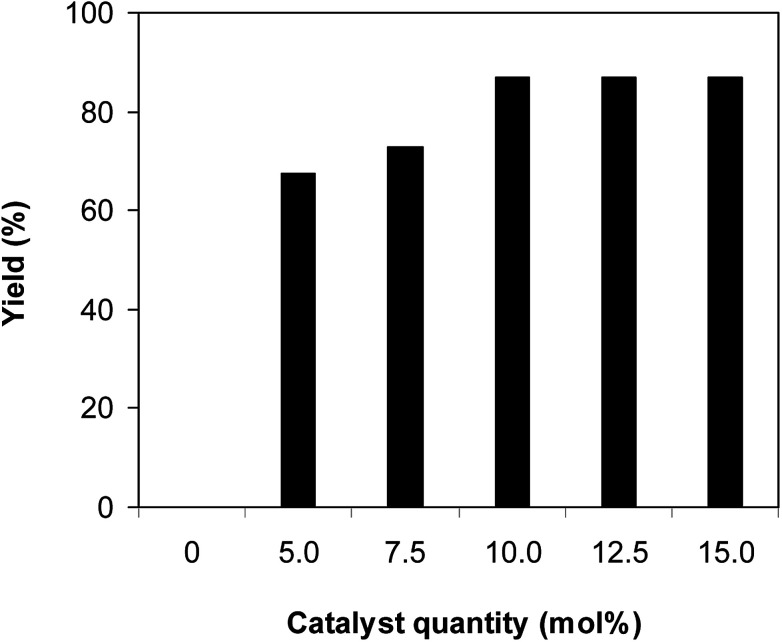
Yield of (*Z*)-2-(phenylsulfonyl)-1-(thiophen-2-yl)ethenamine *vs.* catalyst quantity.

One more factor that should be explored is the reactant concentration. We consequently conducted the reaction with various concentrations of 1-(thiophen-2-yl)ethanone *O*-acetyl oxime, ranging from 0.05 M to 0.50 M. Very low yield of the β-sulfonylvinylamine product was detected with reactant concentration of 0.05 M. Extending the concentration to 0.17 M afforded 39% yield. Interestingly, increasing the reactant concentration to 0.25 M significantly accelerated the transformation, affording 87% yield of (*Z*)-2-(phenylsulfonyl)-1-(thiophen-2-yl)ethenamine. However, lower yield of the desired product was observed for the reaction with higher reactant concentration (Fig. 4). This could be explained due to the mass transfer of the reactants when a solid catalyst was employed. Having these results, the impact of catalyst quantity on the reaction was consequently explored. The reaction was performed at 100 °C in chlorobenzene under argon for 3 h, with 1.2 equivalents of sodium benzenesulfinate and reactant concentration of 0.25 M, in the presence of 5 mol%, 7.5 mol%, 10 mol%, 12.5 mol%, and 15 mol% catalyst, respectively. It was noted that no trace evidence of the β-sulfonylvinylamine product was detected in the absence of the catalyst. Utilizing 5 mol% catalyst resulted in 67% yield, while 73% yield was obtained for the case of 7.5 mol% catalyst. The yield of (*Z*)-2-(phenylsulfonyl)-1-(thiophen-2-yl)ethenamine was improved to 87% for the reaction utilizing 10 mol% catalyst. Extending the catalyst amount to 15 mol% did not led to higher yield of the desired product (Fig. 5). Noted that benzenesulfinic acid was inactive in this reaction, with the desired product being detected in trace amount. Additionally, potassium benzenesulfinate was significantly less reactive than the sodium salt, affording only 16% yield.

**Fig. 6:**
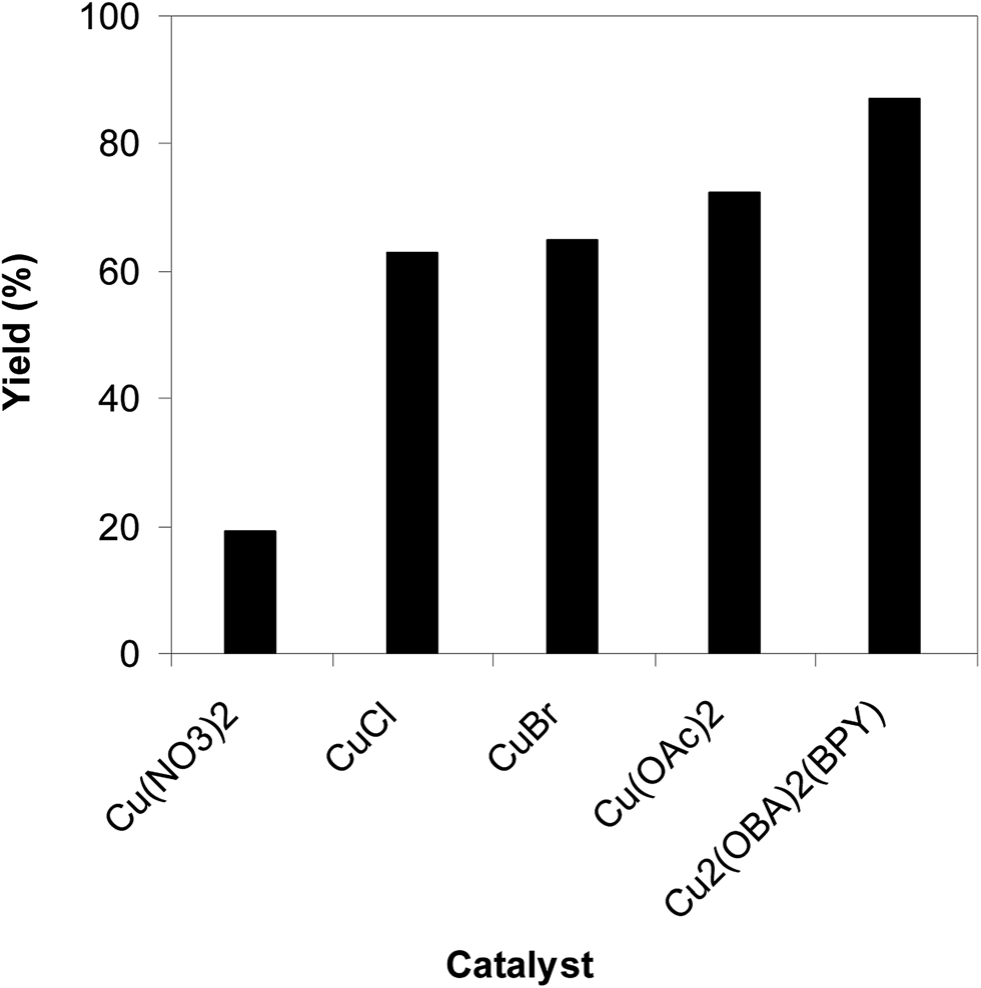
Yield of (*Z*)-2-(phenylsulfonyl)-1-(thiophen-2-yl)ethenamine *vs.* homogeneous catalyst.

**Fig. 7:**
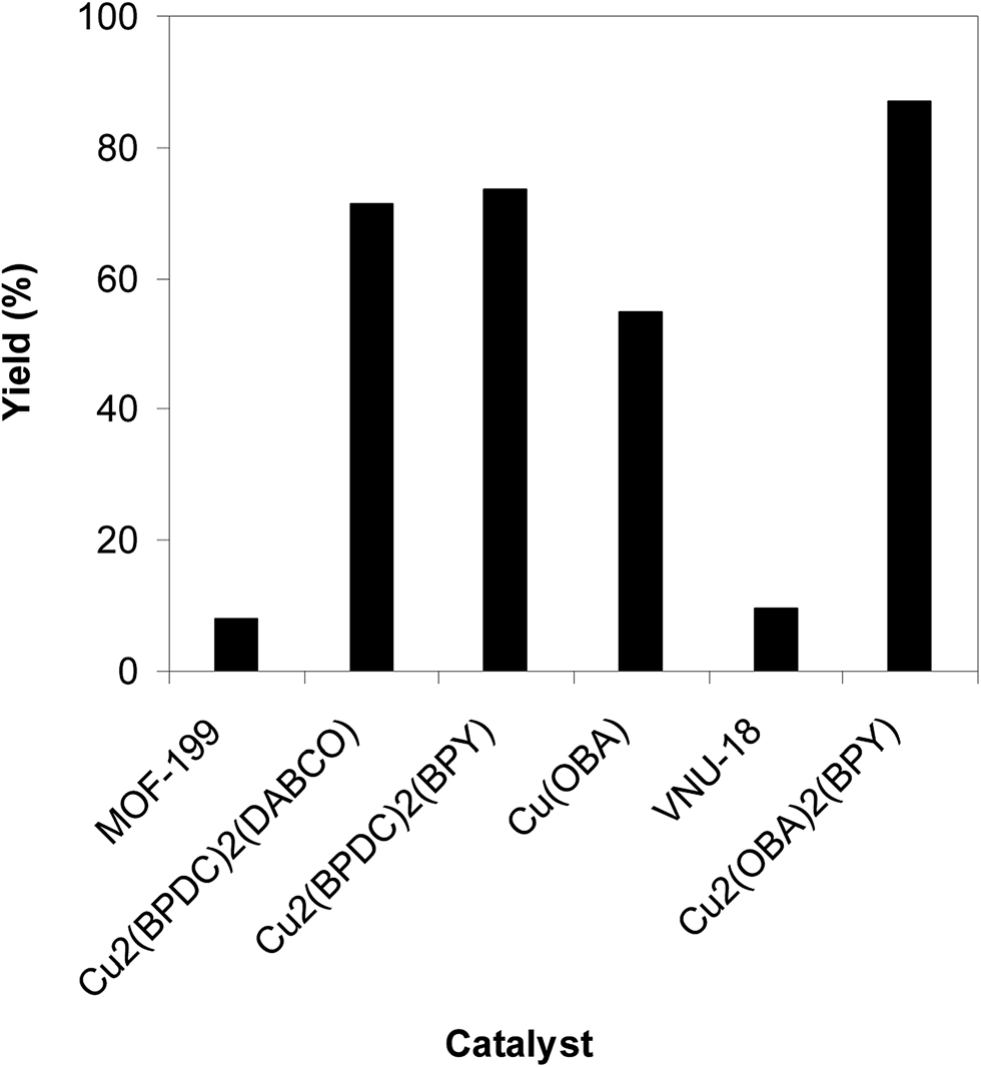
Yield of (*Z*)-2-(phenylsulfonyl)-1-(thiophen-2-yl)ethenamine *vs.* heterogeneous catalyst.

To highlight the benefit of the Cu_2_(OBA)_2_(BPY) in the direct C–S coupling reaction, its catalytic efficiency was compared to other homogeneous and heterogeneous catalysts. The reaction was conducted at 100 °C in chlorobenzene under argon for 3 h, with 1.2 equivalents of sodium benzenesulfinate and reactant concentration of 0.25 M, in the presence of 10 mol% catalyst. Several homogeneous copper catalysts displayed reasonable activity for the reaction. The CuCl-catalyzed transformation progressed to 63% yield, while 65% yield was detected for the case of CuBr. Utilizing Cu(OAc)_2_ as catalyst, the yield was improved to 72% (Fig. 6). Noted that in the first Cu(OAc)_2_-catalyzed synthesis of β-sulfonylvinylamines from oxime acetates and sodium sulfinates, Tang *et al.* pointed out that thiophene oxime acetate was less reactive than benzene oxime acetate.^10^ Moving to copper-based MOFs as catalysts, it was noted that MOF-199 and VNU-18 exhibited very low activity towards the direct C–S coupling reaction, with less than 10% yield being recorded. Cu(OBA) was more active, affording the β-sulfonylvinylamine product in 55% yield. This value was improved to 71% for the reaction utilizing Cu_2_(BPDC)_2_(DABCO) catalyst, while 74% yield was observed for that employing Cu_2_(BPDC)_2_(BPY) catalyst. Compared to these homogeneous and heterogeneous catalysts, Cu_2_(OBA)_2_(BPY) displayed higher performance, generating the expected product in 87% yield (Fig. 7).

**Fig. 8:**
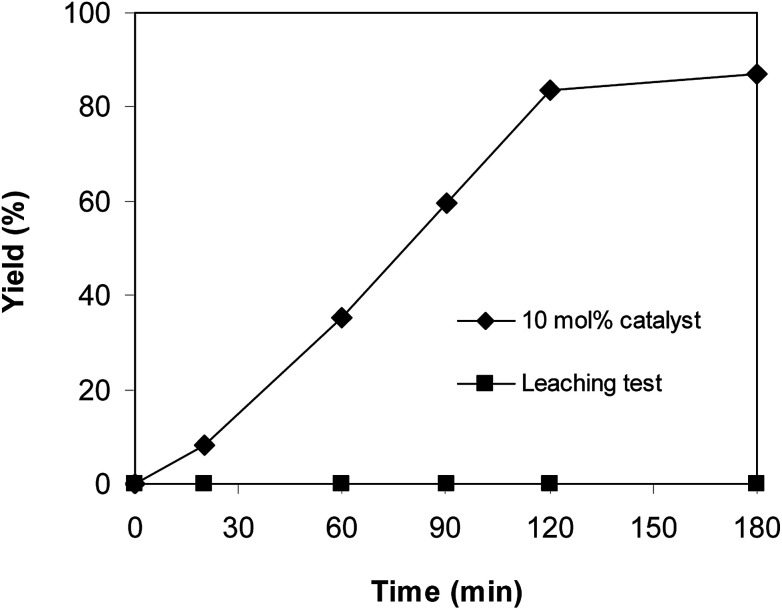
Leaching test verified that the reaction proceeded under truly heterogeneous catalysis.

Since the direct C–S coupling reaction between 1-(thiophen-2-yl)ethanone *O*-acetyl oxime and sodium benzenesulfinate proceeded in chlorobenzene, the leaching experiment was then carried out to confirm that if any copper species in the liquid phase contributed to the yield of (*Z*)-2-(phenylsulfonyl)-1-(thiophen-2-yl)ethenamine. The reaction was performed at 100 °C in chlorobenzene under argon for 3 h, with 1.2 equivalents of sodium benzenesulfinate and reactant concentration of 0.25 M, in the presence of 10 mol% catalyst. Upon the completion of the reaction, the copper-based framework catalyst was removed from the reactor by centrifugation. The liquid phase was subsequently transferred to a new and clean screw-cap vial. Fresh reagents were consequently added to this vial. The resulting mixture was magnetically stirred at 100 °C under argon for 3 h. Under these conditions, no additional (*Z*)-2-(phenylsulfonyl)-1-(thiophen-2-yl)ethenamine was detected. This result verified that the direct C–S coupling reaction utilizing Cu_2_(OBA)_2_(BPY) catalyst progressed under truly heterogeneous catalysis (Fig. 8).

**Scheme 2:**
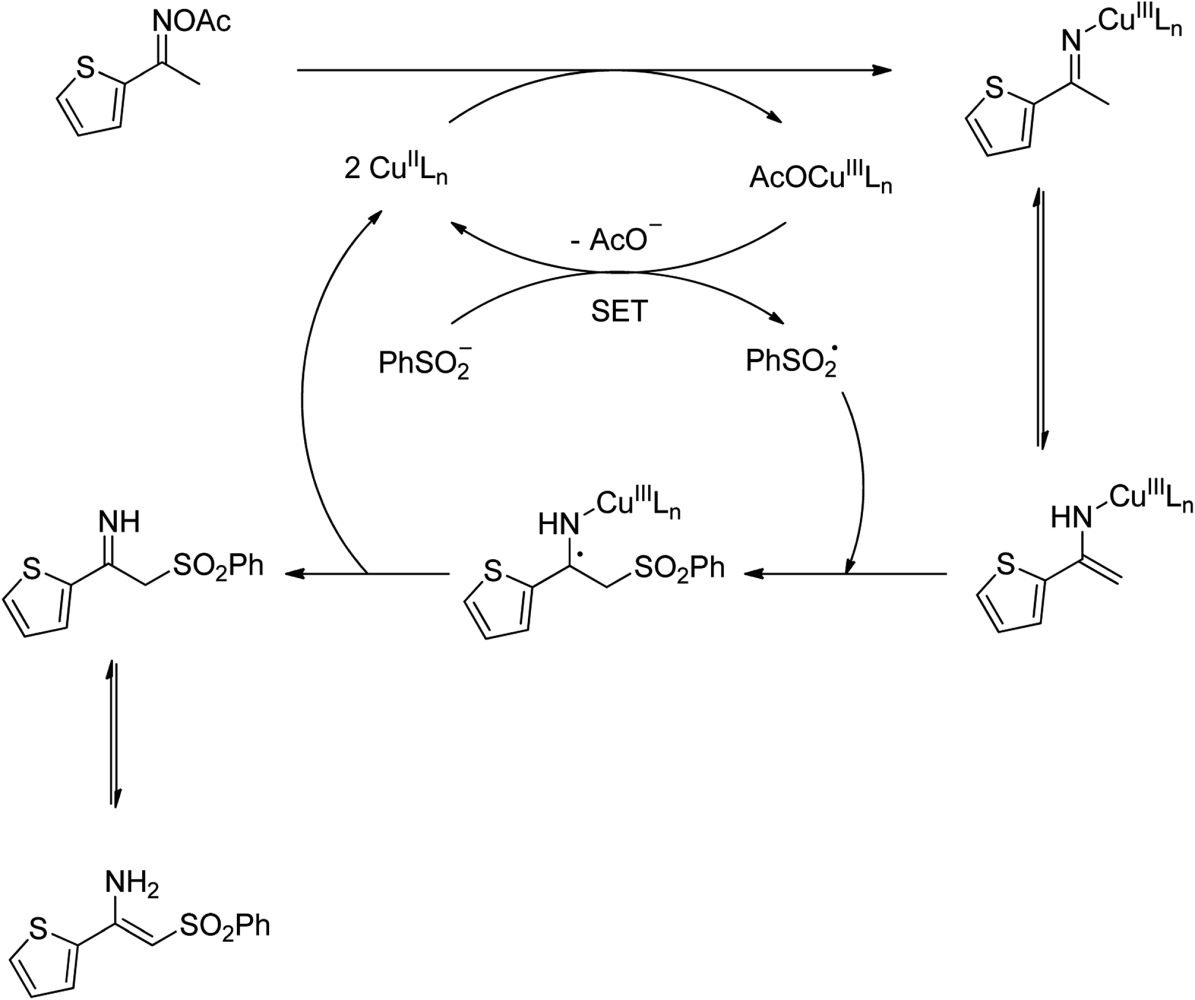
Plausible mechanism for the formation of (*Z*)-2-(phenylsulfonyl)-1-(thiophen-2-yl)ethenamine.

To gain more information about the pathway of the reaction, (2,2,6,6-tetramethylpiperidin-1-yl)oxy (TEMPO) was utilized as a radical scavenger. In the first experiment, TEMPO was added at the beginning of the reaction, and no trace amount of product was detected. In the second experiment, after the first 1 h reaction time, TEMPO was introduced to the reactor, and the reaction mixture was magnetically stirred under argon at 100 °C for further 2 h. Under these conditions, no additional β-sulfonylvinylamine product was observed. Furthermore, the direct C–S coupling reaction was also allowed to proceed in the presence of 1,1-diphenylethylene. It was noted that the transformation was considerably affected by this radical scavenger. These observations verified that radical species should be involved in the catalytic cycle. With these data, and based on previous report,^10^ a plausible pathway was proposed (Scheme 2). Initially, a copper enamide intermediate was produced from the oxime acetate, while copper(ii) were converted to copper(iii) species. A sulfonyl free radical was then formed *via* a single-electron-transfer (SET) process, releasing the copper(ii) species. Next, the sulfonyl free radical attacked to the enamide intermediate, followed by the regeneration of the copper(ii) species. Finally, tautomerization of the imine intermediate afforded the corresponding β-sulfonylvinylamine.

**Fig. 9:**
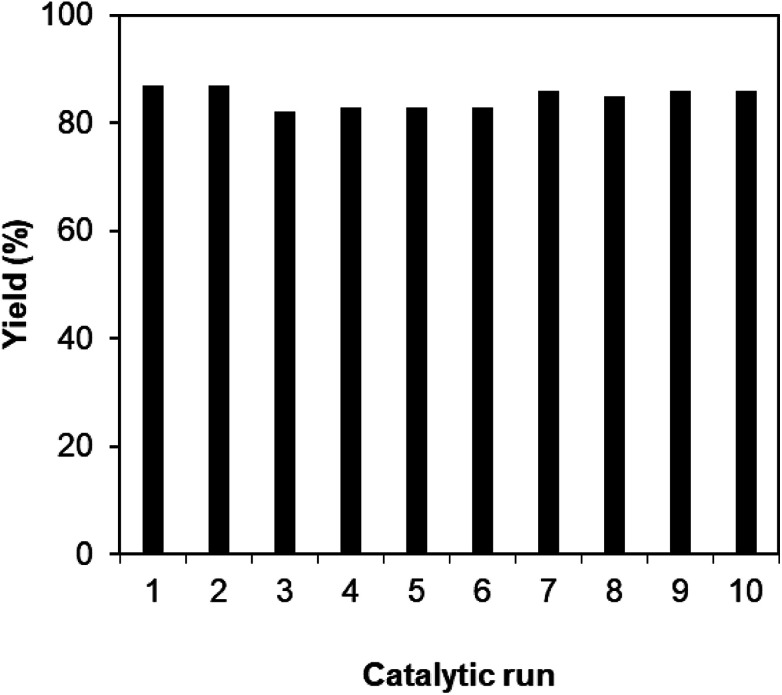
Catalyst reutilizing studies.

**Fig. 10:**
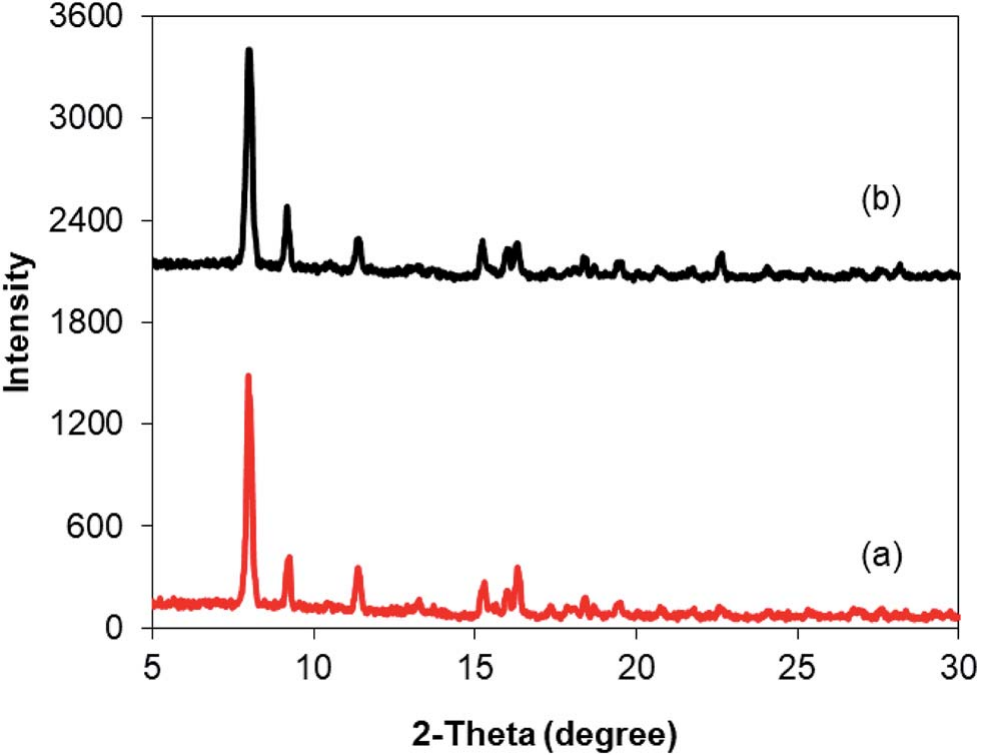
XRD observations of the new (a) and reused (b) Cu_2_(OBA)_2_(BPY).

**Fig. 11:**
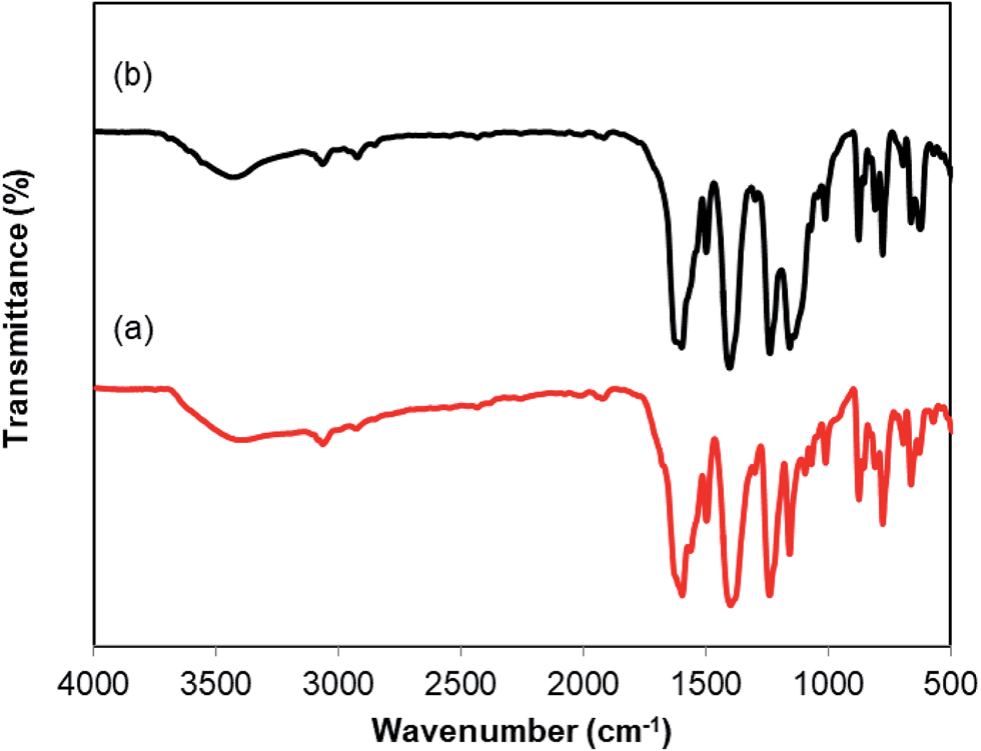
FT-IR spectra of new (a) and reutilized (b) Cu_2_(OBA)_2_(BPY).

As mentioned previously, the Cu_2_(OBA)_2_(BPY) displayed higher catalytic performance than numerous homogeneous and MOF-based catalysts. To emphasize the significance of this catalyst, one important issue would be its reusability. The copper-based framework was accordingly explored for reusability in 10 sequential catalytic runs. The reaction was carried out at 100 °C in chlorobenzene under argon for 3 h, with 1.2 equivalents of sodium benzenesulfinate and reactant concentration of 0.25 M, in the presence of 10 mol% catalyst. After each catalytic cycle, the copper-based catalyst was collected by centrifugation, washed extensively with DMF and methanol, dried at 150 °C under vacuum on a Shlenkline for 6 h. This catalyst sample was subsequently utilized for next catalytic run with the same reaction conditions. It was noted that the Cu_2_(OBA)_2_(BPY) was reusable many times without a substantial deterioration in catalytic efficiency. Certainly, 86% yield of the desired product was still obtained for the 10^th^ catalytic run (Fig. 9). Additionally, the reutilized catalyst was characterized by XRD (Fig. 10) and FT-IR (Fig. 11). Analysis results verified that the catalyst structure was preserved during the experiments.

With the significance of β-ketosulfones in pharmaceutical and agrochemical industries, we expanded the work to achieve these valuable structures *via* a hydrolysis step (Scheme 1b and Table 1). The reaction was performed at 100 °C in chlorobenzene under argon for 3 h, with 1.2 equivalents of sodium benzenesulfinate and reactant concentration of 0.25 M, in the presence of 10 mol% catalyst. After that, the catalyst was recovered, the reaction mixture was treated with aqueous HCl solution, and the expected β-ketosulfone was purified by silica gel column chromatography. Following this procedure, 2-(phenylsulfonyl)-1-(thiophen-2-yl)ethanone was obtained in 76% yield (entry 1). Tang *et al.* previously demonstrated the Cu(OAc)_2_-catalyzed synthesis of β-sulfonylvinylamines from oxime acetates and sodium sulfinates, and upon hydrolysis, corresponding β-ketosulfones were produced in high yields.^10^ It was reported that thiophene-based ketoximes were less reactive than benzene-based ketoximes. In this work, we also found that 1-*p*-tolylethanone *O*-acetyl oxime was more reactive than 1-(thiophen-2-yl)ethanone *O*-acetyl oxime, and 2-(phenylsulfonyl)-1-*p*-tolylethanone was generated in 88% yield (entry 2).

**Table 1:** Synthesis of β-ketosulfones *via* Cu_2_(OBA)_2_(BPY)-catalyzed direct C–S coupling reaction, followed by hydrolysis step

Entry	Reactant 1	Reactant 2	Product	Isolated yield
1	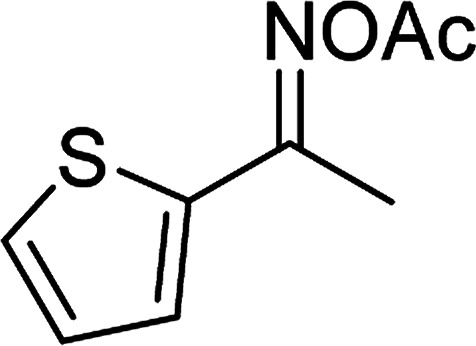	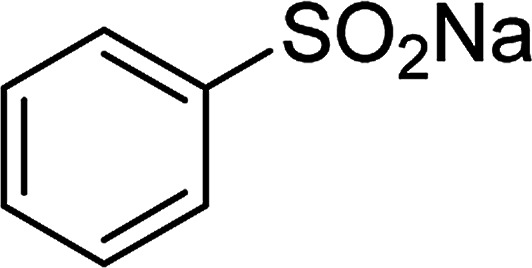	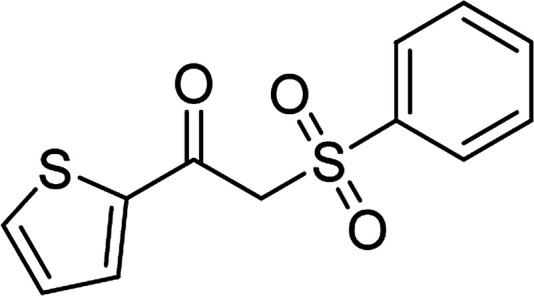	76%
2	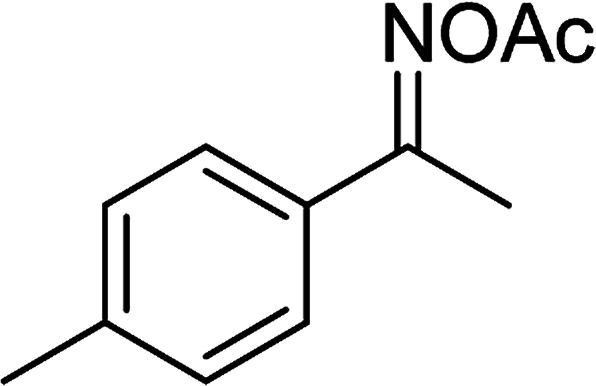	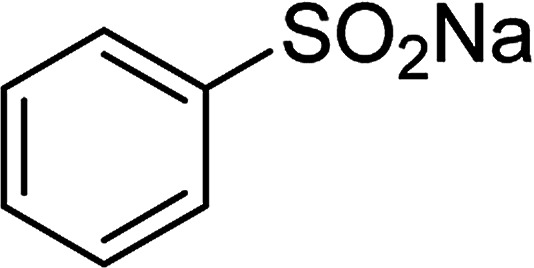	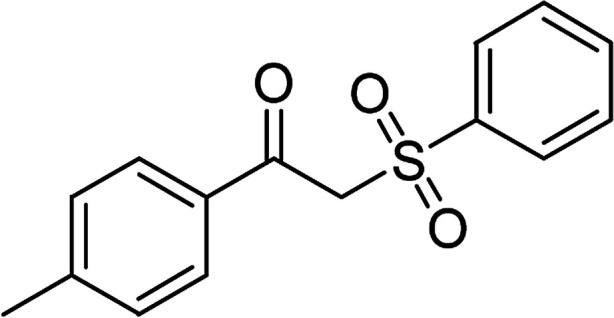	88%
3	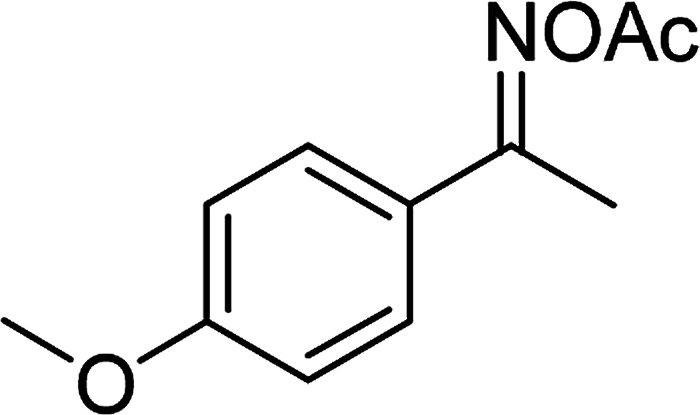	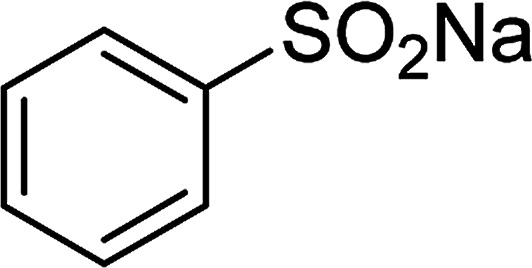	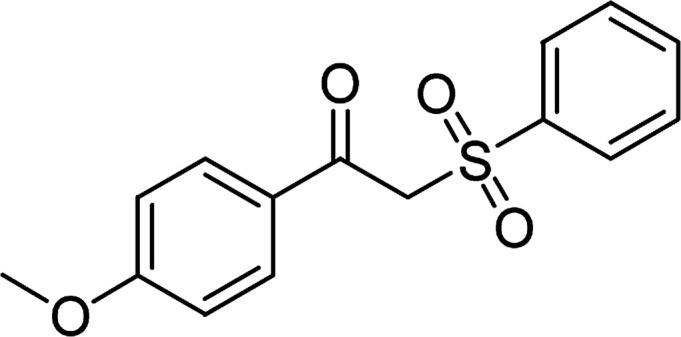	91%
4	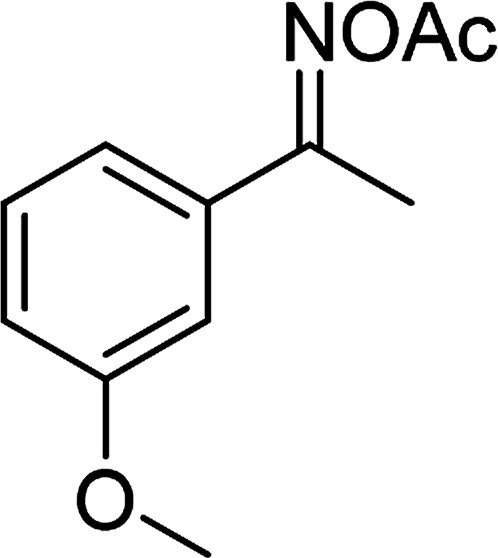	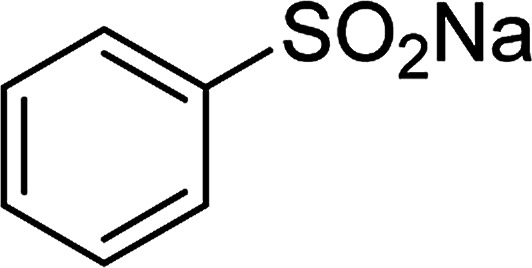	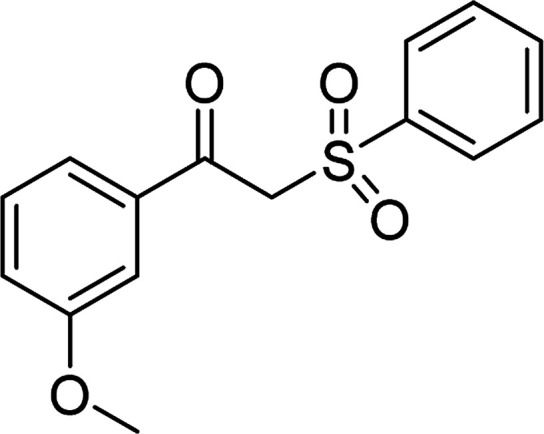	89%
5	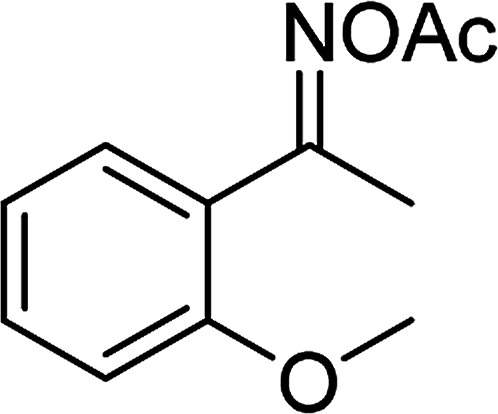	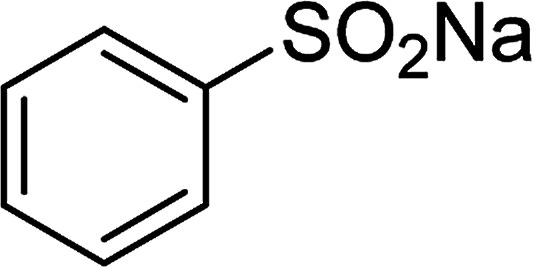	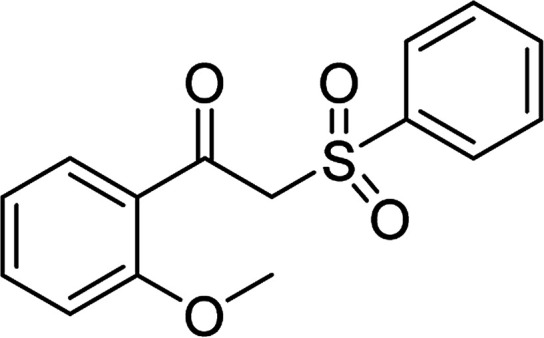	83%
6	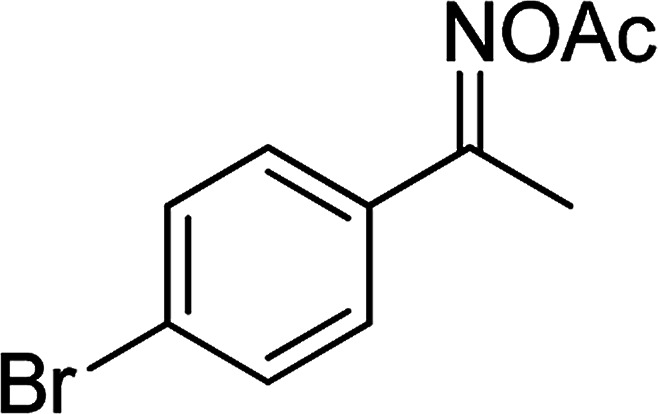	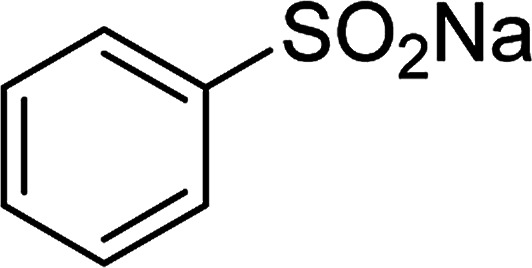	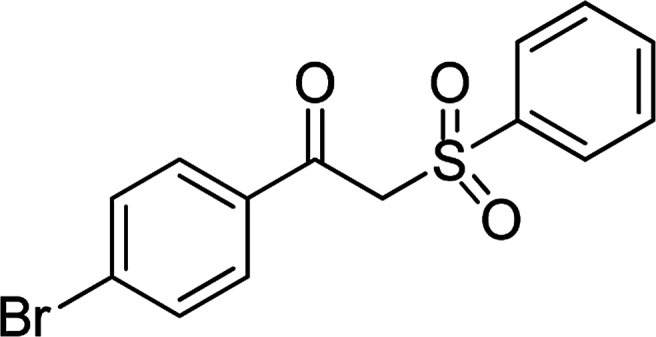	76%
7	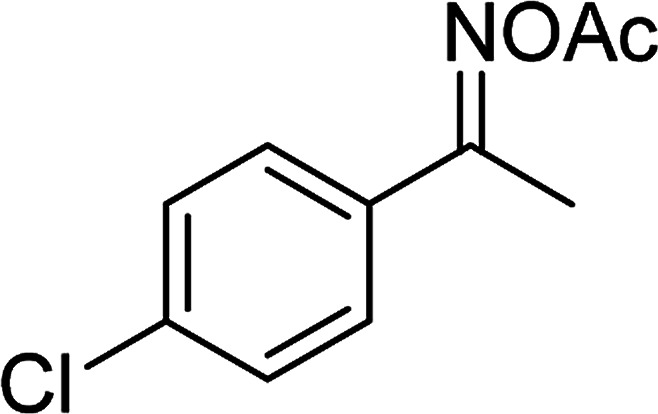	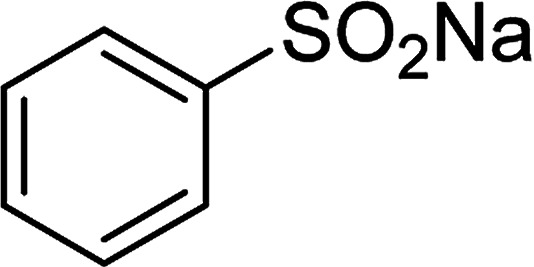	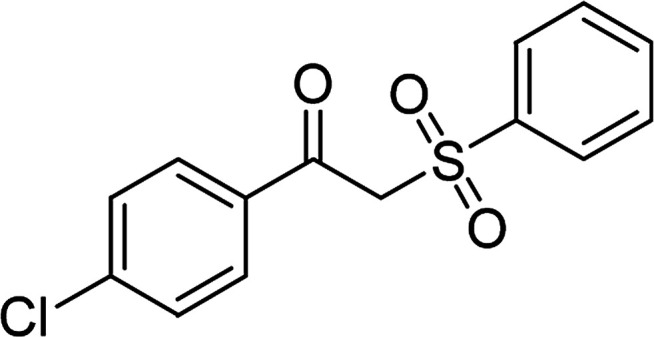	80%
8	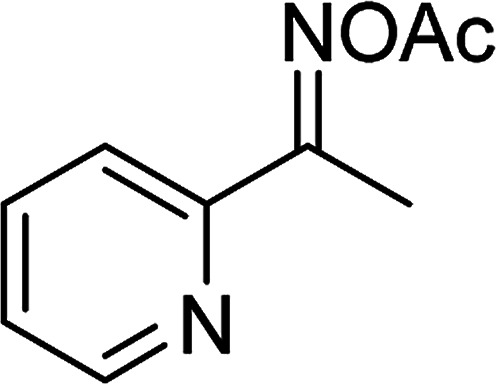	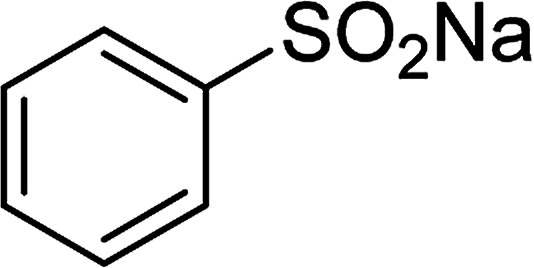	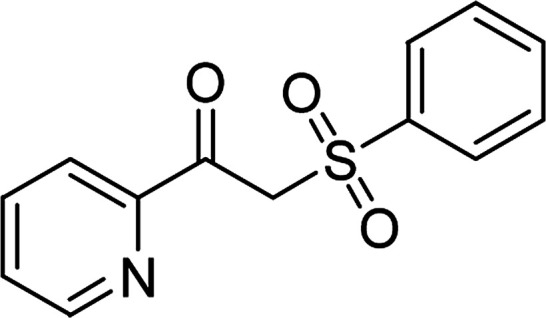	80%
9	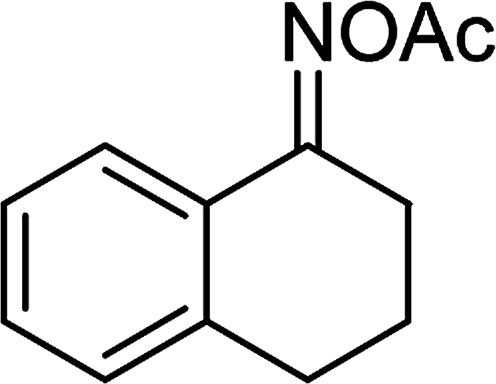	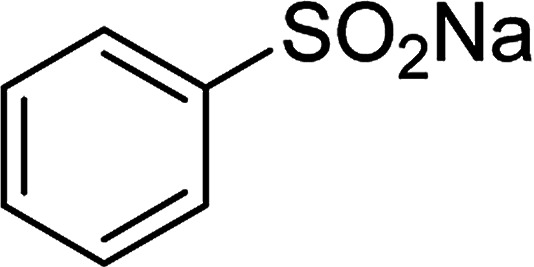	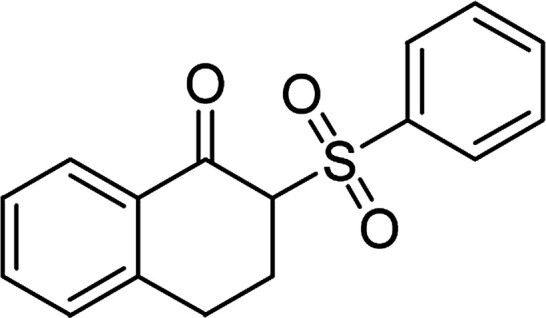	82%
10	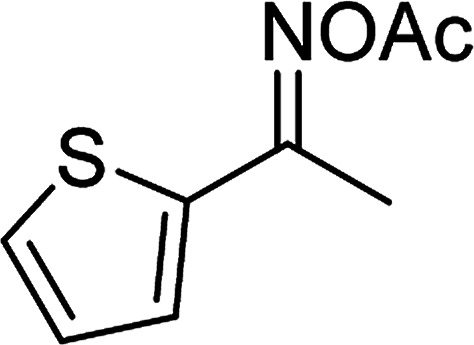	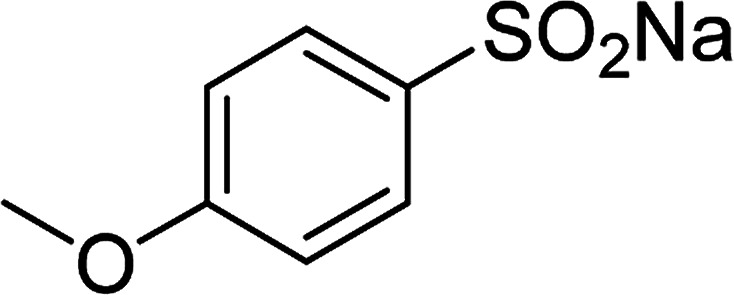	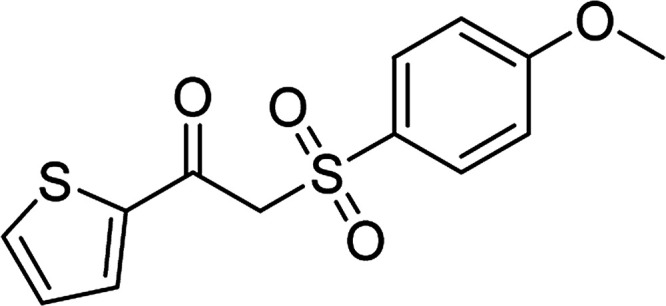	90%
11	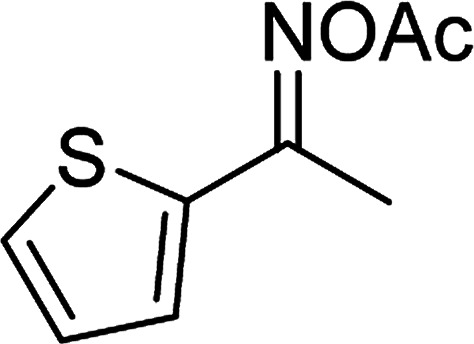	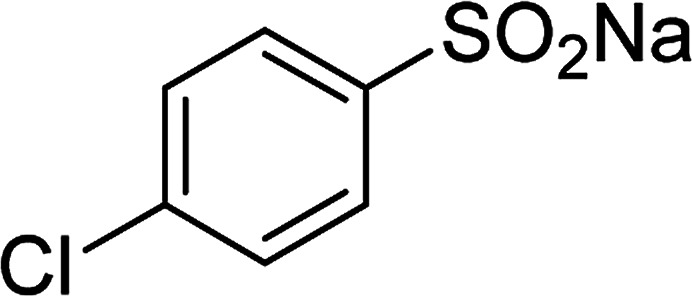	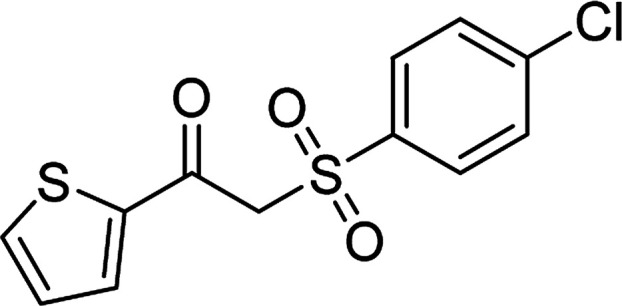	78%
12	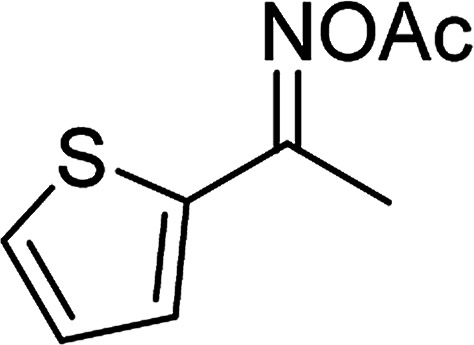	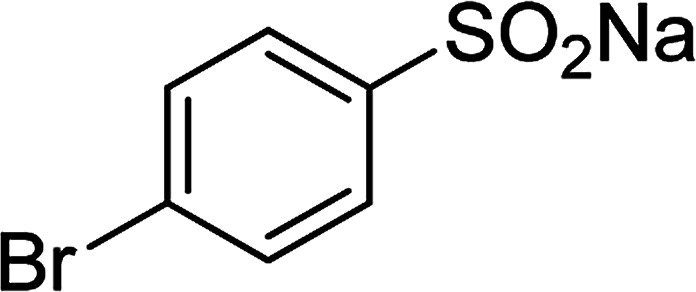	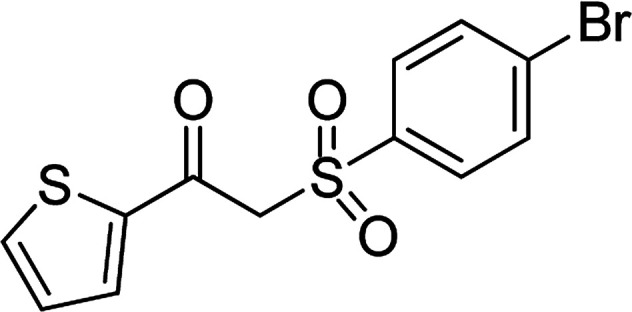	65%
13	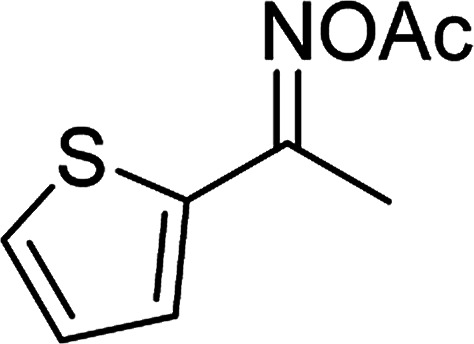	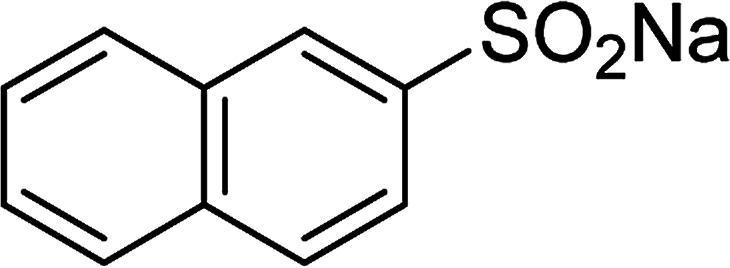	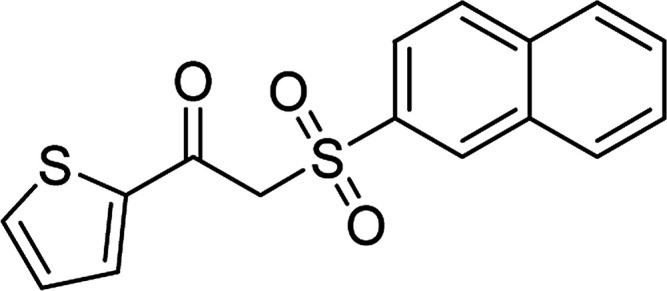	74%
14	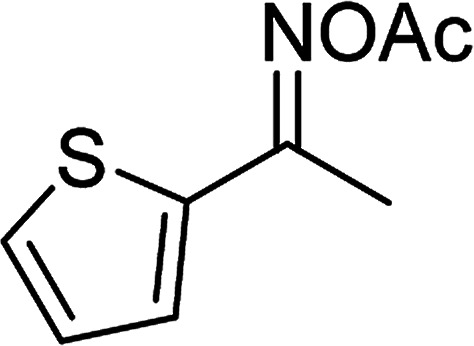	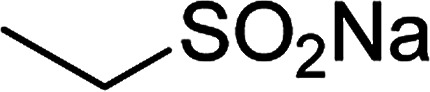	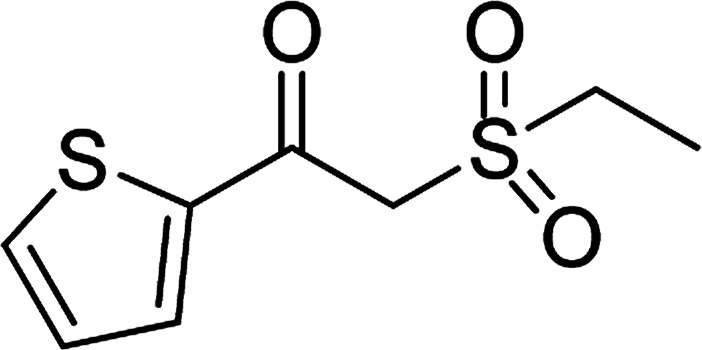	79%

Several benzene-based ketoximes were utilized in the synthesis of β-ketosulfones *via* Cu_2_(OBA)_2_(BPY)-catalyzed direct C–S coupling reaction, followed by hydrolysis step. 1-(4-Methoxyphenyl)-2-(phenylsulfonyl)ethanone was achieved in 91% (entry 3). Similarly, the reactions of meta- and ortho-substituted ketoximes afforded the corresponding products in high yields (entries 4 and 5). Ketoximes possessing a halogen substituent were slightly less reactive, forming 1-(4-bromophenyl)-2-(phenylsulfonyl)ethanone, and 1-(4-chlorophenyl)-2-(phenylsulfonyl)ethanone in 76%, and 80% yields, respectively (entries 6 and 7). 2-(Phenylsulfonyl)-1-(pyridin-2-yl)ethanone was also obtained in 80% yield (entry 8). 3,4-Dihydronaphthalen-1(2*H*)-one *O*-acetyl oxime was also reactive, and 82% yield of 2-(phenylsulfonyl)-3,4-dihydronaphthalen-1(2*H*)-one was recorded (entry 9). Different sodium benzenesulfinates were also used, and 2-(4-methoxyphenylsulfonyl)-1-(thiophen-2-yl)ethanone (entry 10), 2-(4-chlorophenylsulfonyl)-1-(thiophen-2-yl)ethanone (entry 11), 2-(4-bromophenylsulfonyl)-1-(thiophen-2-yl)ethanone (entry 12), and 2-(naphthalen-2-ylsulfonyl)-1-(thiophen-2-yl)ethanone (entry 13) were generated in 90%, 78%, 65%, and 74% yields, respectively. Additionally, the transformation of sodium ethanesulfinate afforded 2-(ethylsulfonyl)-1-(thiophen-2-yl)ethan-1-one in 79% yield (entry 14).

## 4. Conclusions

The copper–organic framework Cu_2_(OBA)_2_(BPY) was utilized as a recyclable heterogeneous catalyst for the direct C–S coupling reaction between sodium sulfinates and oxime acetates to produce β-sulfonylvinylamines. The solvent expressed a remarkable impact on the transformation, and chlorobenzene emerged as the best option. This Cu-MOF was more active towards the coupling reaction than a series of copper-based homogeneous and MOF-based catalysts. The coupling reaction utilizing Cu_2_(OBA)_2_(BPY) catalyst progressed under truly heterogeneous catalysis. The catalyst was reutilized many times in the synthesis of β-sulfonylvinylamines without a considerably deterioration in catalytic efficiency. These β-sulfonylvinylamines were promptly converted to corresponding β-ketosulfones *via* a hydrolysis step with aqueous HCl solution. The fact that β-sulfonylvinylamines were generated under a heterogeneous catalysis approach, and readily converted to β-ketosulfones upon hydrolysis, was consequently profitable to pharmaceutical and agrochemical industries.

## Conflicts of interest

There are no conflicts to declare.

## Supplementary Material

RA-008-C8RA02389A-s001
