# Supplementary material for: An efficient access to β-ketosulfones via β-sulfonylvinylamines: metal–organic framework catalysis for the direct C–S coupling of sodium sulfinates with oxime acetates
Source: RSC Adv. 2018 May 14;8(31):17477–85. doi: 10.1039/c8ra02389a (PMC9080424; doi:10.1039/c8ra02389a)
Supplement: RA-008-C8RA02389A-s001 [file RA-008-C8RA02389A-s001.pdf]

Electronic Supplementary Material (ESI) for RSC Advances.
This journal is © The Royal Society of Chemistry 2018

An efficient acess to β-ketosulfones via β-sulfonylvinylamines: Metal-organic framework catalysis for the coupling of sodium sulfinates with oxime acetates

Tuong A. To, Chau B. Tran, Ngoc T. H. Nguyen, Hai H. T. Nguyen, Anh T. Nguyen, Anh N. Q. Phan, Nam T. S. Phan*

Faculty of Chemical Engineering, HCMC University of Technology, VNU-HCM,

268 Ly Thuong Kiet, District 10, Ho Chi Minh City, Viet Nam

*Email: ptsnam@hcmut.edu.vn

Ph: (+84 8) 38647256 ext. 5681 Fx: (+84 8) 38637504

Supporting Information

Materials and instrumentation

All reagents and starting materials were obtained commercially from Sigma-Aldrich and Merck, and were used as received without any further purification unless otherwise noted. Nitrogen physisorption measurements were conducted using a Micromeritics 2020 volumetric adsorption analyzer system. Samples were pretreated by heating under vacuum at 150 ^{o}C for 3 h. A Netzsch Thermoanalyzer STA 409 was used for thermogravimetric analysis (TGA) with a heating rate of 10 ^{o}C/min under a nitrogen atmosphere. X-ray powder diffraction (XRD) patterns were recorded using a Cu Kα radiation source on a D8 Advance Bruker powder diffractometer. Scanning electron microscopy studies were conducted on a S4800 Scanning Electron Microscope (SEM). Transmission electron microscopy studies were performed using a JEOL JEM 1400 Transmission Electron Microscope (TEM) at 100 kV. The sample was dispersed on holey carbon

grids for TEM observation. Elemental analysis with inductively coupled plasma (ICP) was performed on a Shimadzu ICPE-9000. Fourier transform infrared (FT-IR) spectra were obtained on a Nicolet 6700 instrument, with samples being dispersed on potassium bromide pallets.

Gas chromatographic (GC) analyses were performed using a Shimadzu GC 2010-Plus equipped with a flame ionization detector (FID) and an SPB-5 column (length = 30 m, inner diameter = 0.25 mm, and film thickness = 0.25 μm). In the GC temperature program, the sample of the reaction was held at 100°C for 1 min; heated from 100 to 280°C at 40°C/min; held at 280°C for 6.5 min. GC yields of the reaction were calculated using *n*-dodecane as an internal standard. GC-MS analyses were performed using a Shimadzu GCMS-QP2010Ultra with a ZB-5MS column (length = 30 m, inner diameter = 0.25 mm, and film thickness = 0.25 μm). The temperature program for GC-MS analysis held samples at 50 °C for 2 min; heated samples from 50 to 280°C at 10 °C/min and held them at 280 °C for 10 min. Inlet temperature was set constant at 280 °C. MS spectra were compared with the spectra gathered in the NIST library. The ^{1}H NMR and ^{13}C NMR were recorded on Bruker AV 500 spectrometers using residual solvent peak as a reference.

General procedure to prepare oxime acetates

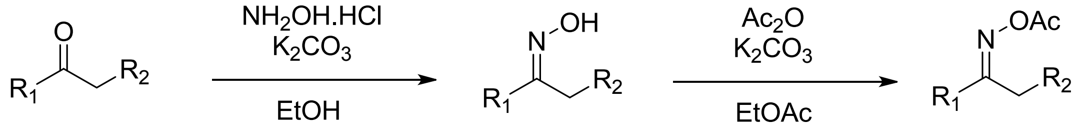

Ketones (22 mmol), K_2CO_3 (4.264 g, 30.9 mmol), hydroxylamine hydrochloride ($NH_2OH.HCl$) (2.29 g, 33 mmol) and ethanol (10 mL) were magnetically stirred at 60 ^oC for 1 h. The reaction mixture was cooled to room temperature, quenched with H_2O then organic components were extracted with ethyl acetate (2 x 30 mL) and washed with brine (2 x 30 mL) then neutralized by HCl 1 M. The organic layers were dried over anhydrous Na_2SO_4 and concentrated under vacuum to obtain the crude oximes which were used on the next step without purification. The crude oximes and K_2CO_3 (22.2 mmol) were added to the mixture of anhydride acetic (4.2 mL, 44.4 mmol) and ethyl acetate (10 mL), then stirred at room temperature for 1 h. The next work-up procedure was conducted similarly to that of previous step, yielding solid product. It could be further washed with hexane, if necessary.

General procedure to prepare sodium sulfinates

Commercial sodium sulfinates were purchased from suppliers and were used as received without further purification. Other sulfinate salts were prepared from their corresponding sulfonyl chlorides by the following procedure.

$$RSO_2Cl \quad + \quad Na_2SO_3 \xrightarrow[H_2O]{NaHCO_3} RSO_2Na$$

A mixture of sulfonyl chlorides (20 mmol), sodium sulfite (5.04 g, 40 mmol) and sodium bicarbonate (3.36 g, 40 mmol) were heated and magnetically stirred in water (20 mL) at 80 ^oC for 8 h. After cooling to room temperature, water was removed under vacuum. Recrystallization of the residues in ethanol afforded the sodium sulfinates.

Catalyst characterization

X-ray powder diffraction observation showed a highly sharp peak with 2θ of 7.98 ^{o}, proving that the copper-based framework was crystalline (Fig. S1). The scanning electron microscopy image demonstrated that well-formed crystals were obtained (Fig. S2). Nevertheless, transmission electron microscopy micrograph did not provide a clear pore structure of the framework (Fig. S3). Nitrogen physisorption analysis results indicated that the Cu-MOF would be microporous with diameters of less than 20 Å being recorded (Fig. S4). Langmuir surface areas of 379 m^2/g were noticed for the $Cu_2(OBA)_2(BPY)$, calculated using nitrogen adsorption/desorption isotherm data (Fig. S5). Thermal degradation studies revealed that the Cu-MOF would be stable over 300 ^{o}C (Fig. S6). Fourier Transform-Infrared Spectroscopy results of the $Cu_2(OBA)_2(BPY)$ displayed an obvious difference as compared to those of 4,4′-oxybis(benzoic) acid and 4,4′-bipyridine linkers (Fig. S7).

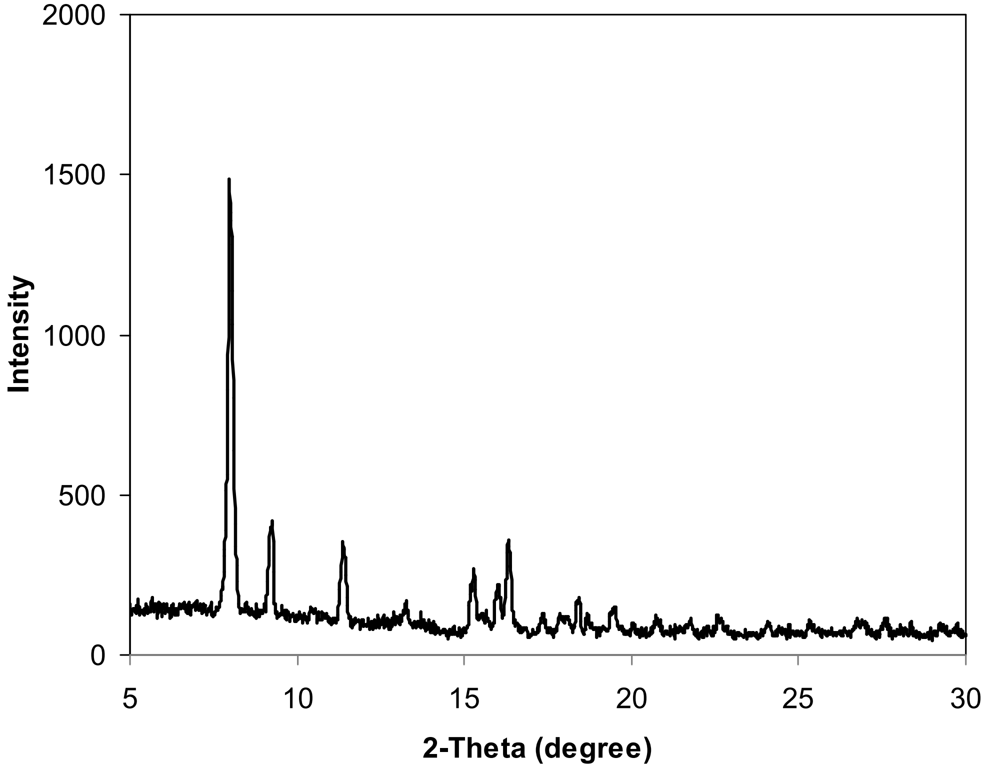

Fig. S1. X-ray powder diffractograms of the $Cu_2(OBA)_2(BPY)$.

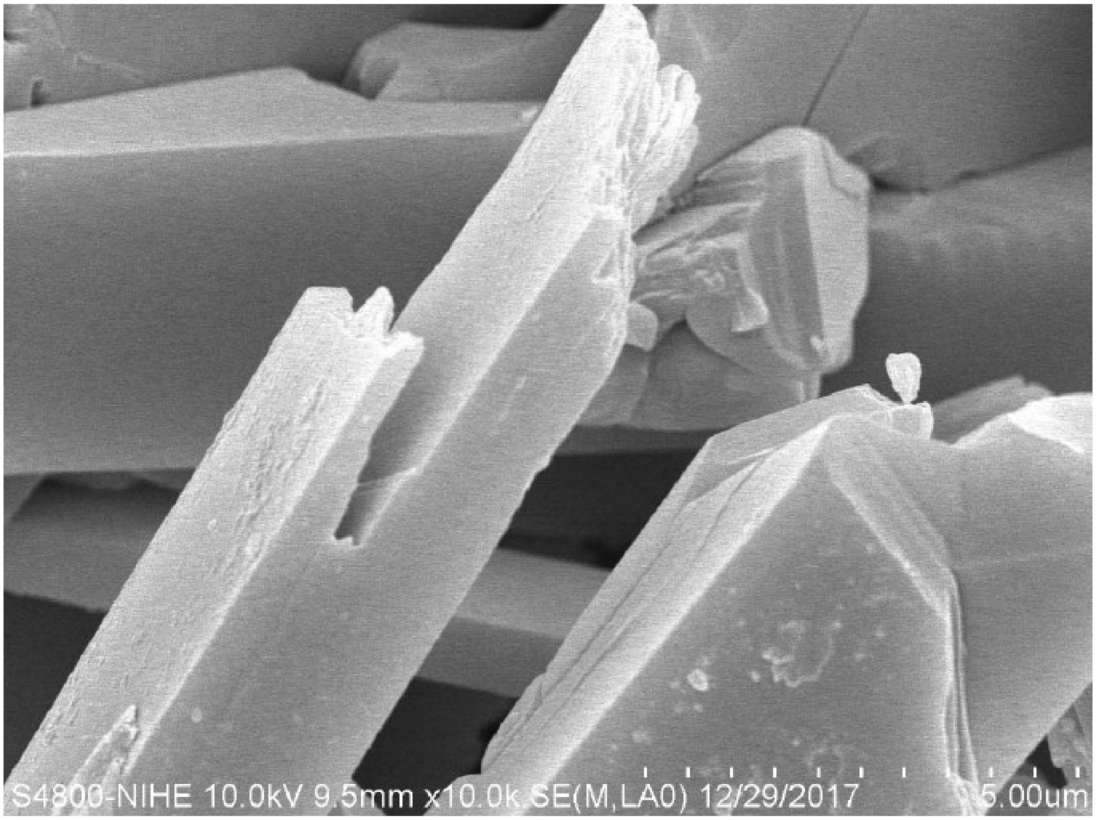

Fig. S2. SEM micrograph of the $Cu_2(OBA)_2(BPY)$.

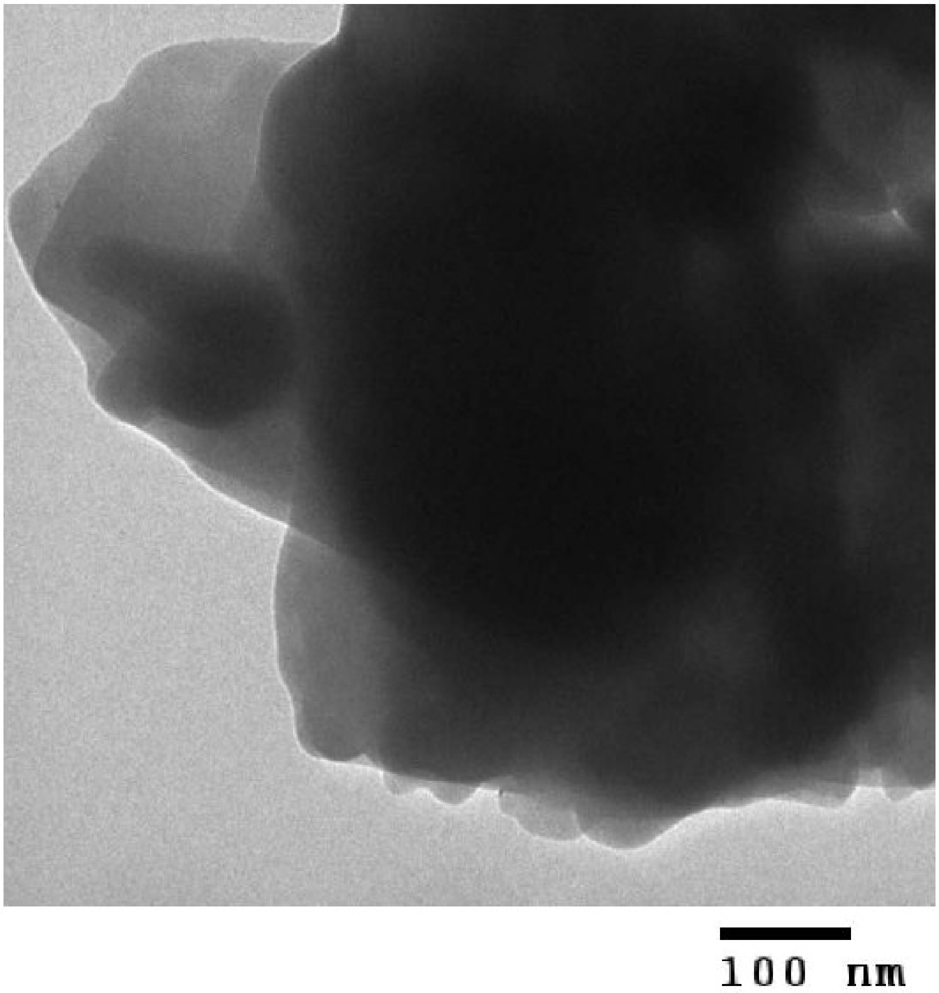

Fig. S3. TEM micrograph of the $Cu_2(OBA)_2(BPY)$.

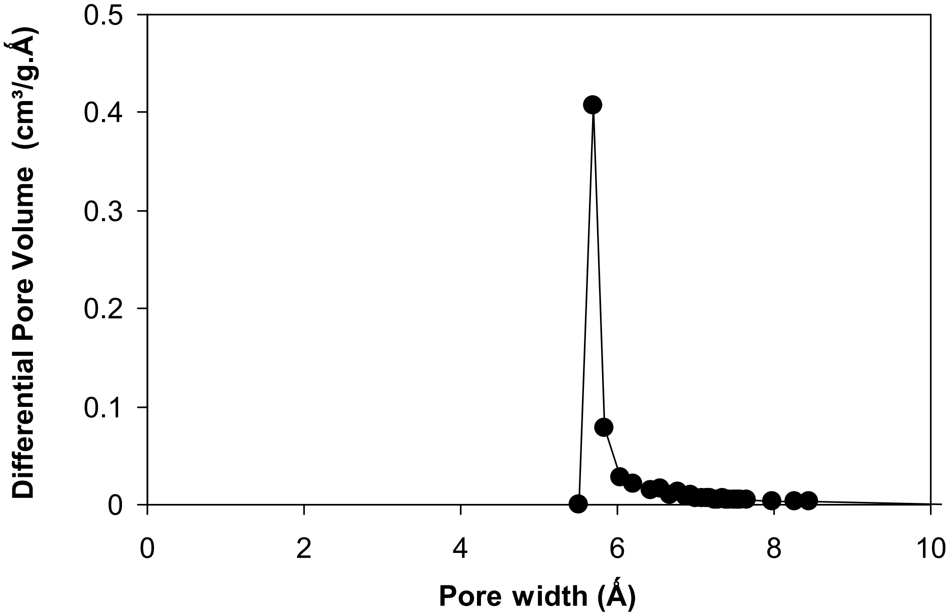

Fig. S4. Pore size distribution of the $Cu_2(OBA)_2(BPY)$.

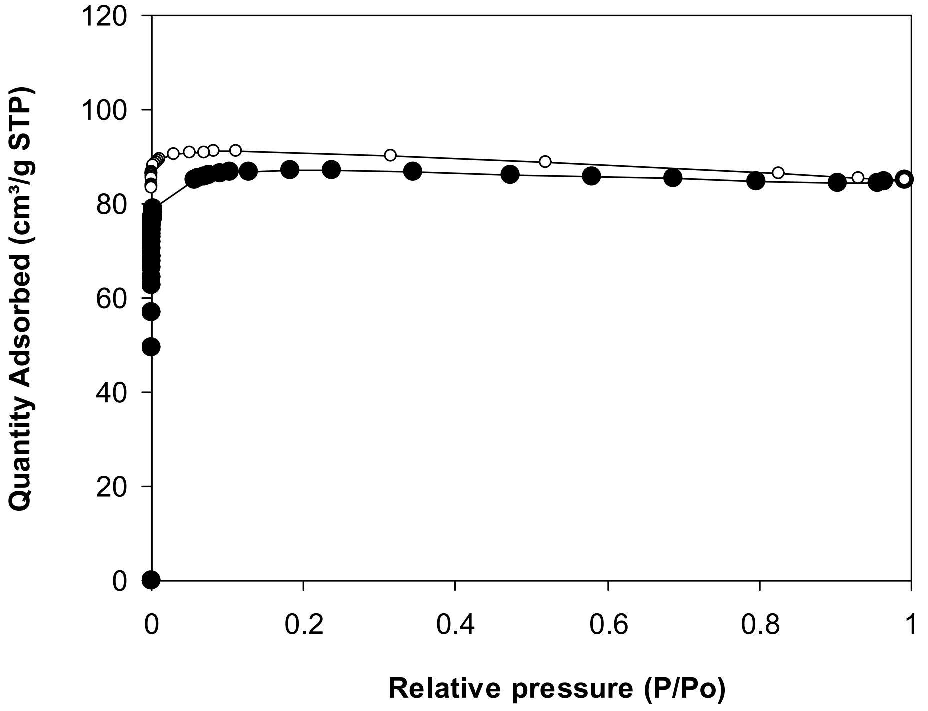

Fig. S5. Nitrogen adsorption/desorption isotherm of the $Cu_2(OBA)_2(BPY)$. Adsorption data are shown as closed circles and desorption data as open circles.

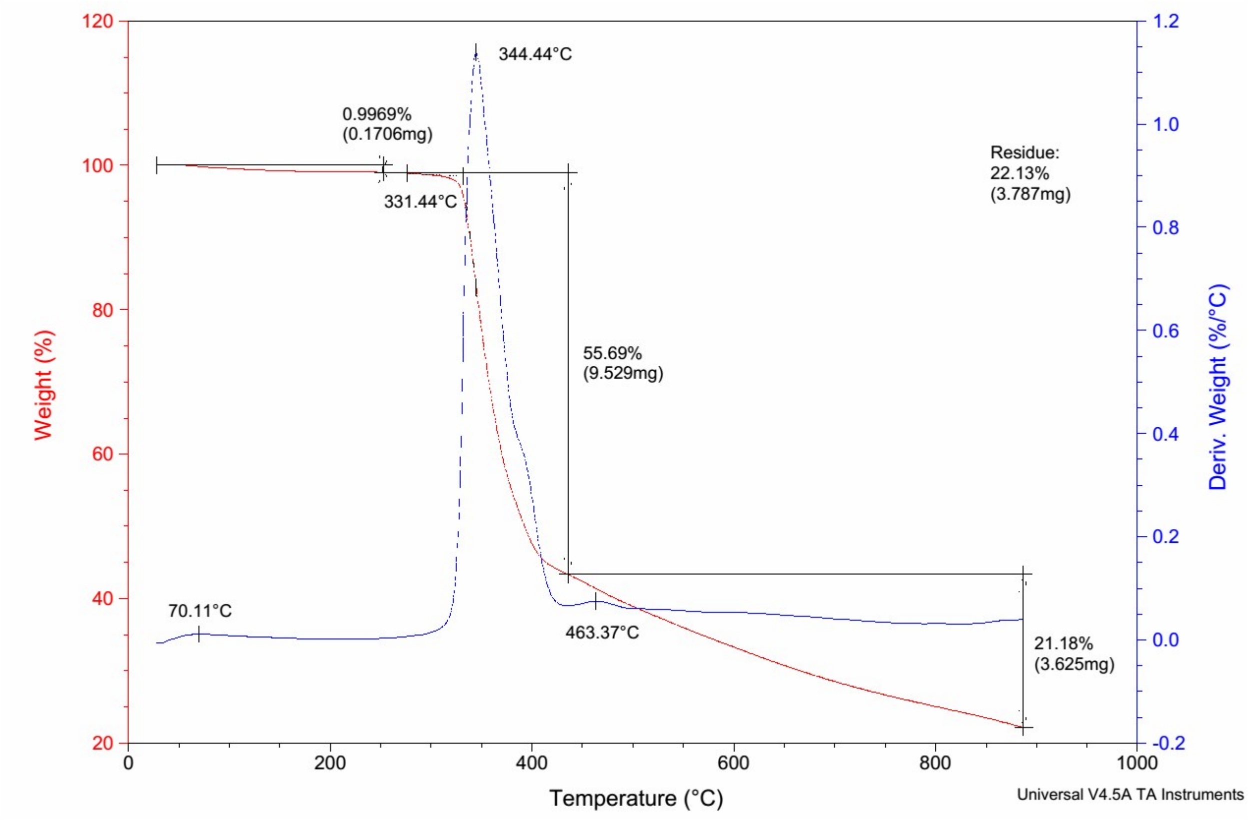

Fig. S6. TGA analysis of the $Cu_2(OBA)_2(BPY)$.

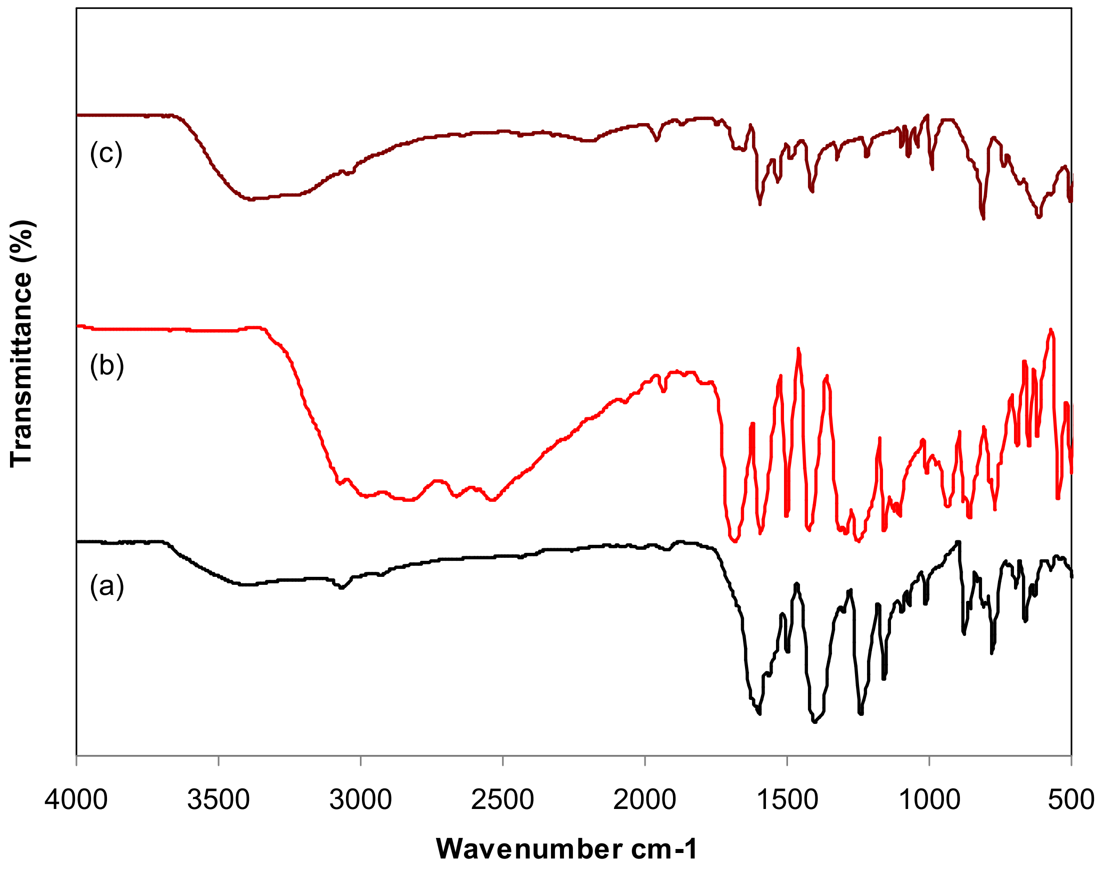

Fig. S7. FT-IR spectra of $Cu_2(OBA)_2(BPY)$ (a), H_2OBA (b), 4,4'-bipyridine (c).

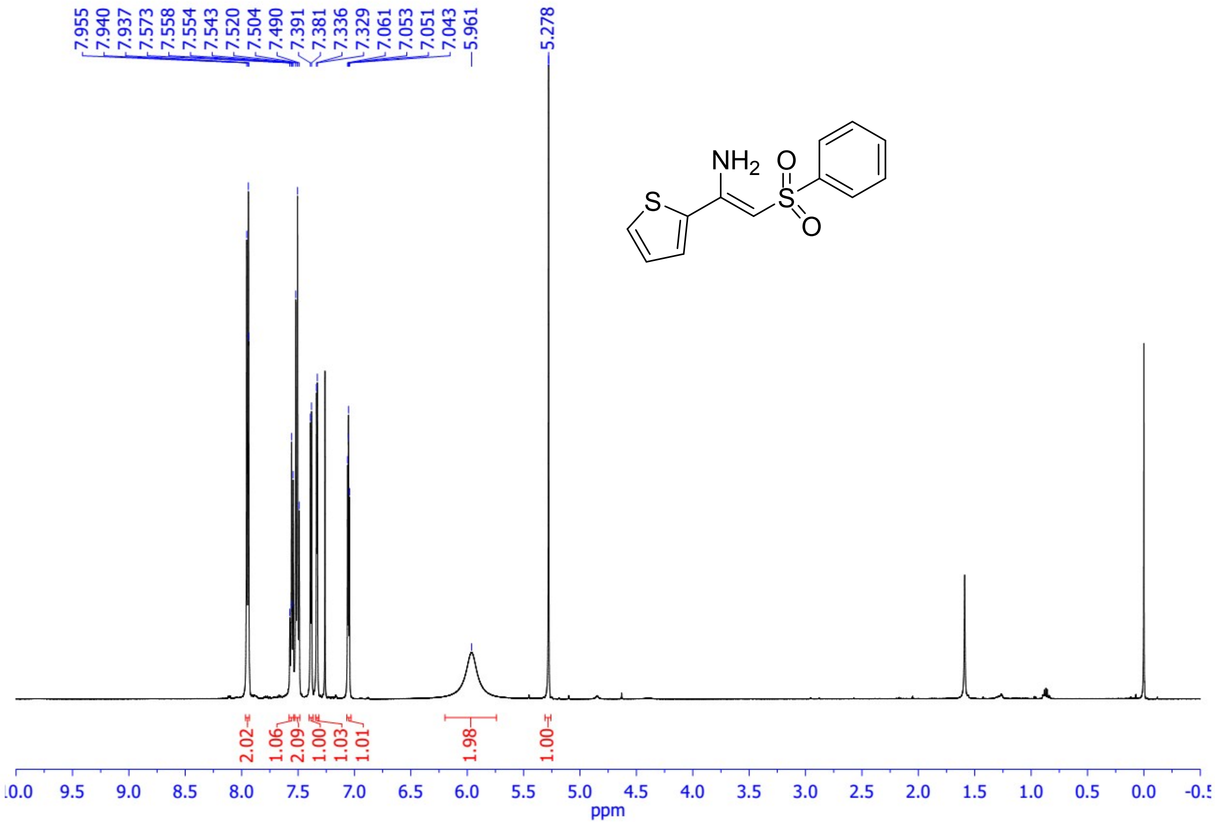

Fig. S8. ^{1}H-NMR spectra of (Z)-2-(phenylsulfonyl)-1-(thiophen-2-yl)ethenamine

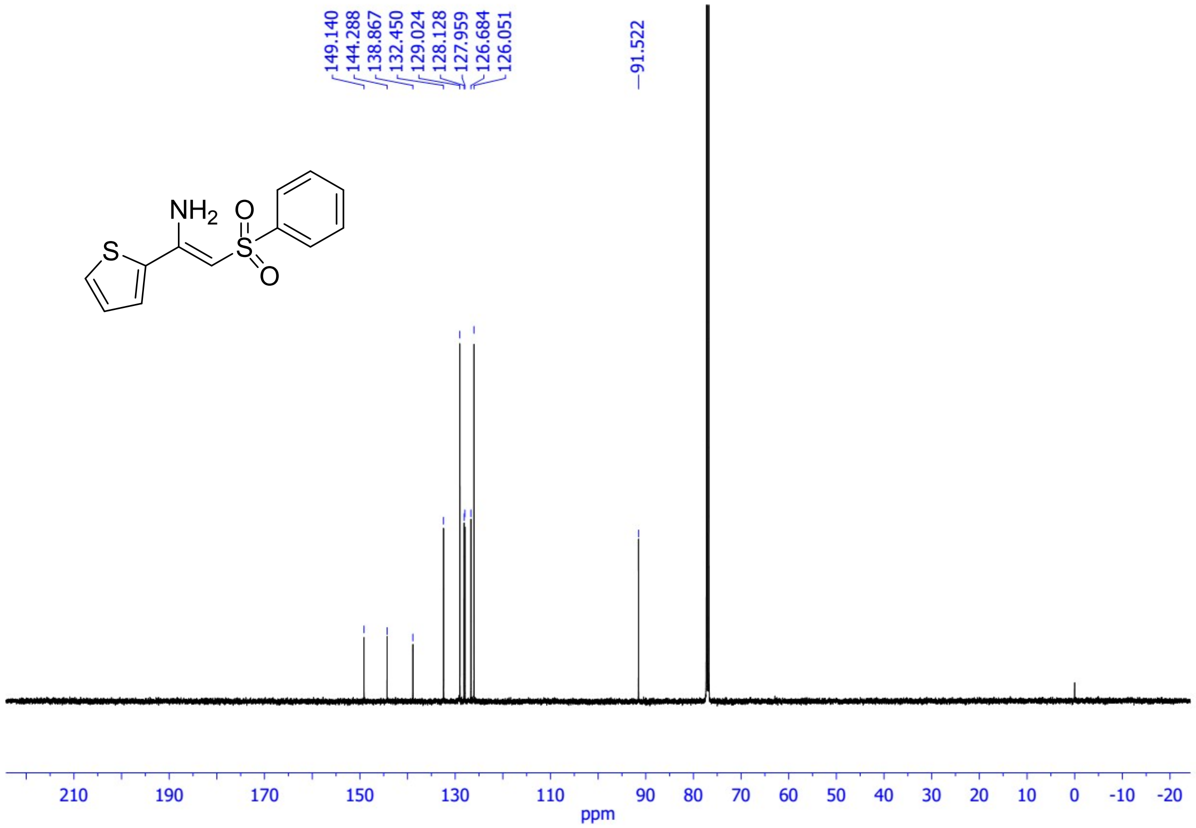

Fig. S9. ^{13}C-NMR spectra of (Z)-2-(phenylsulfonyl)-1-(thiophen-2-yl)ethenamine

Characterization Data for (Z)-2-(phenylsulfonyl)-1-(thiophen-2-yl)ethenamine

Prepared as shown in the general experimental procedure and purified by recrystallization in chlorobenzene and hexane: white crystal, 76% yield. ^{1}H NMR (500 MHz, $CDCl_3$) δ 7.96 – 7.93 (m, 2H), 7.58 – 7.40 (m, 1H), 7.53 – 7.48 (m, 2H), 7.40 – 7.37 (m, 1H), 7.35 – 7.33 (m, 1H), 7.05 (dd, J = 5, 4 Hz, 1H), 5.96 (br, 2H), 5.28 (s, 1H). ^{13}C NMR (125 MHz, $CDCl_3$) δ = 149.1, 144.3, 138.9, 132.4, 129.0, 128.1, 128.0, 126.7, 126.0, 91.5.

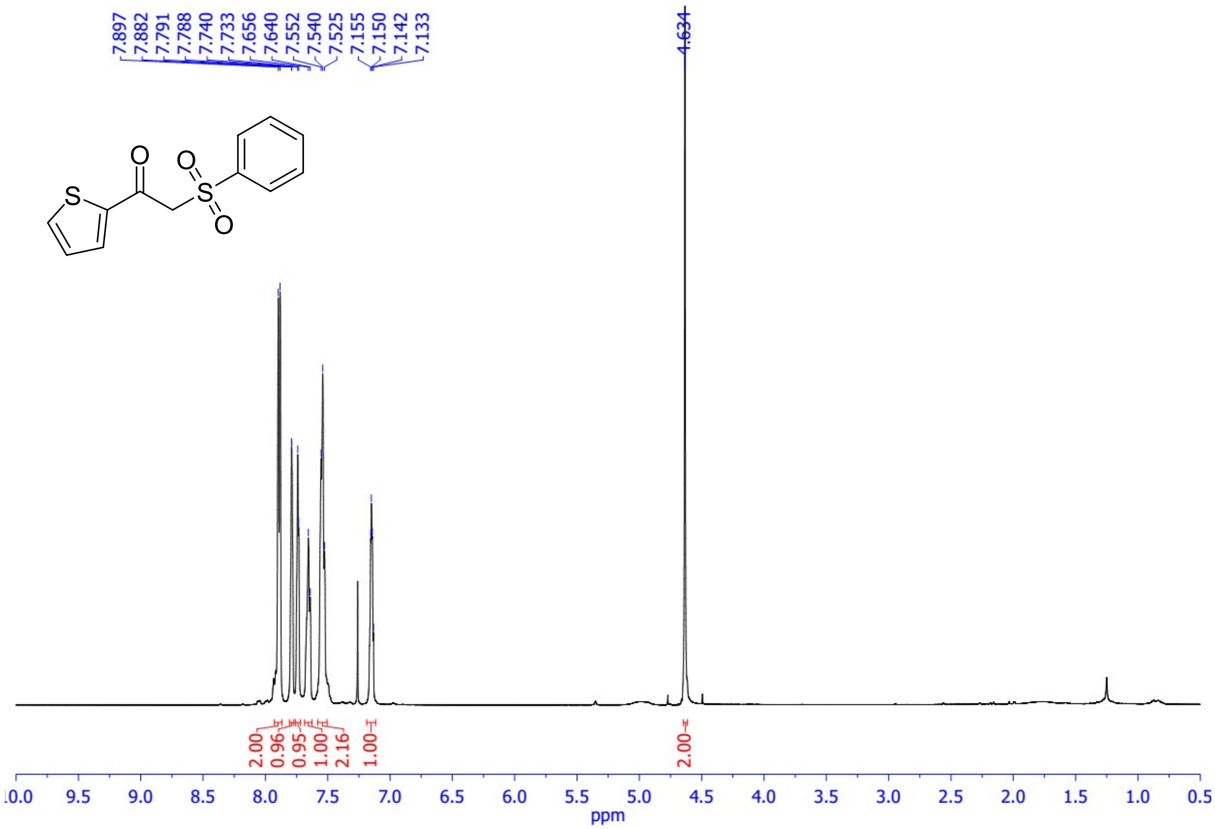

Fig. S10. ^{1}H-NMR spectra of 2-(phenylsulfonyl)-1-(thiophen-2-yl)ethanone

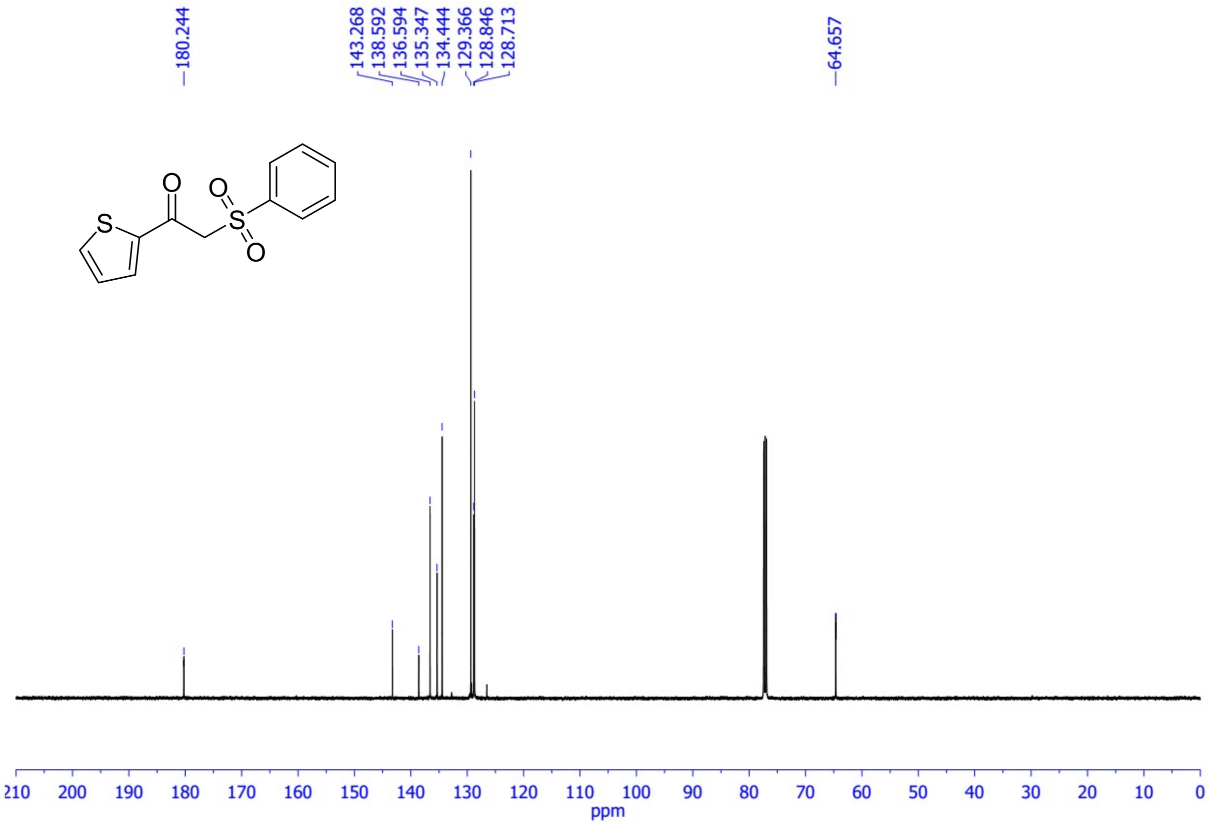

Fig. S11. ^{13}C-NMR spectra of 2-(phenylsulfonyl)-1-(thiophen-2-yl)ethanone

Characterization Data for 2-(phenylsulfonyl)-1-(thiophen-2-yl)ethanone

Prepared as shown in the general experimental procedure and purified on silica gel (hexane/ethyl acetate = 3:1): white solid, 76% yield. ^{1}H NMR (500 MHz, $CDCl_3$) δ 7.89 (d, J = 7.5 Hz, 2H), 7.81 – 7.77 (m, 1H), 7.76 – 7.72 (m, 1H), 7.68 – 7.63 (m, 1H), 7.57 – 7.52 (m, 2H), 7.18 – 7.13 (m, 1H), 4.63 (s, 2H). ^{13}C NMR (125 MHz, $CDCl_3$) δ = 180.2, 143.3, 138.6, 136.6, 135.4, 134.4, 129.37, 128.8, 128.7, 64.7.

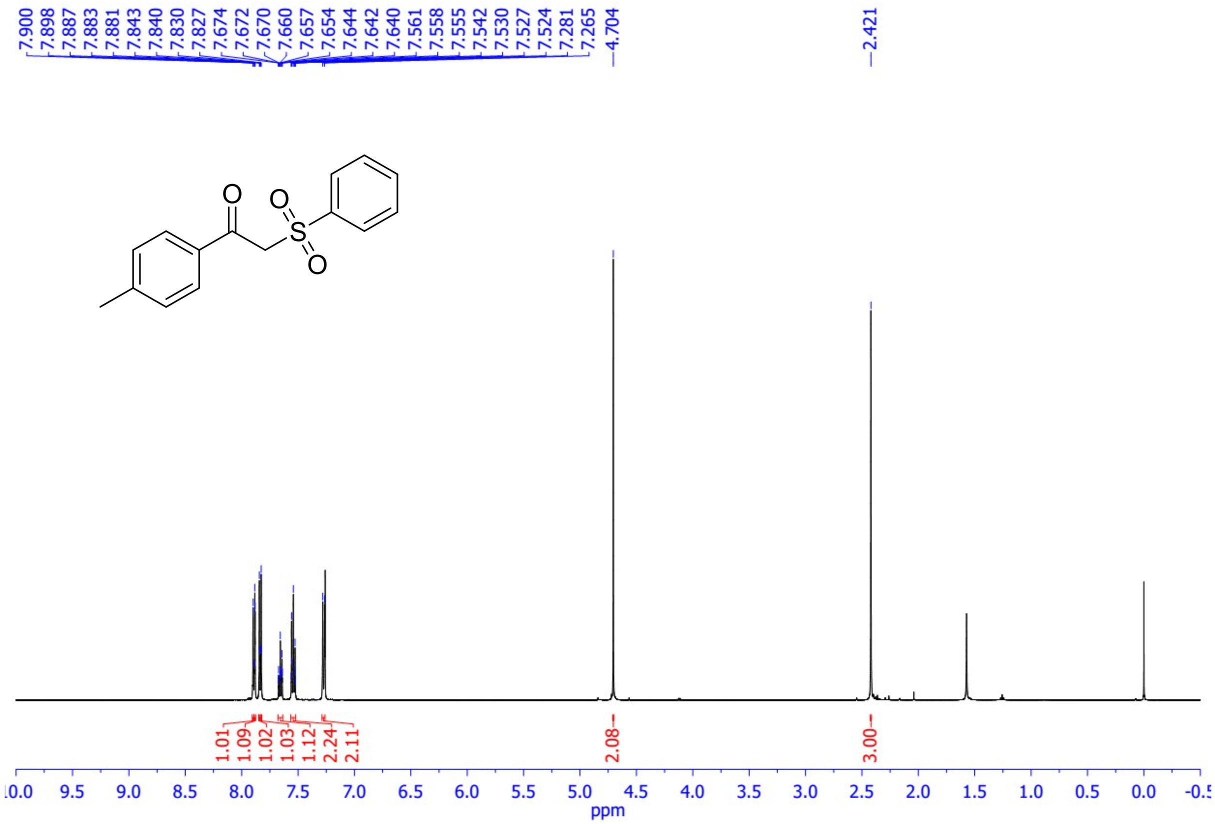

Fig. S12. ^{1}H-NMR spectra of 2-(phenylsulfonyl)-1-(p-tolyl)ethanone

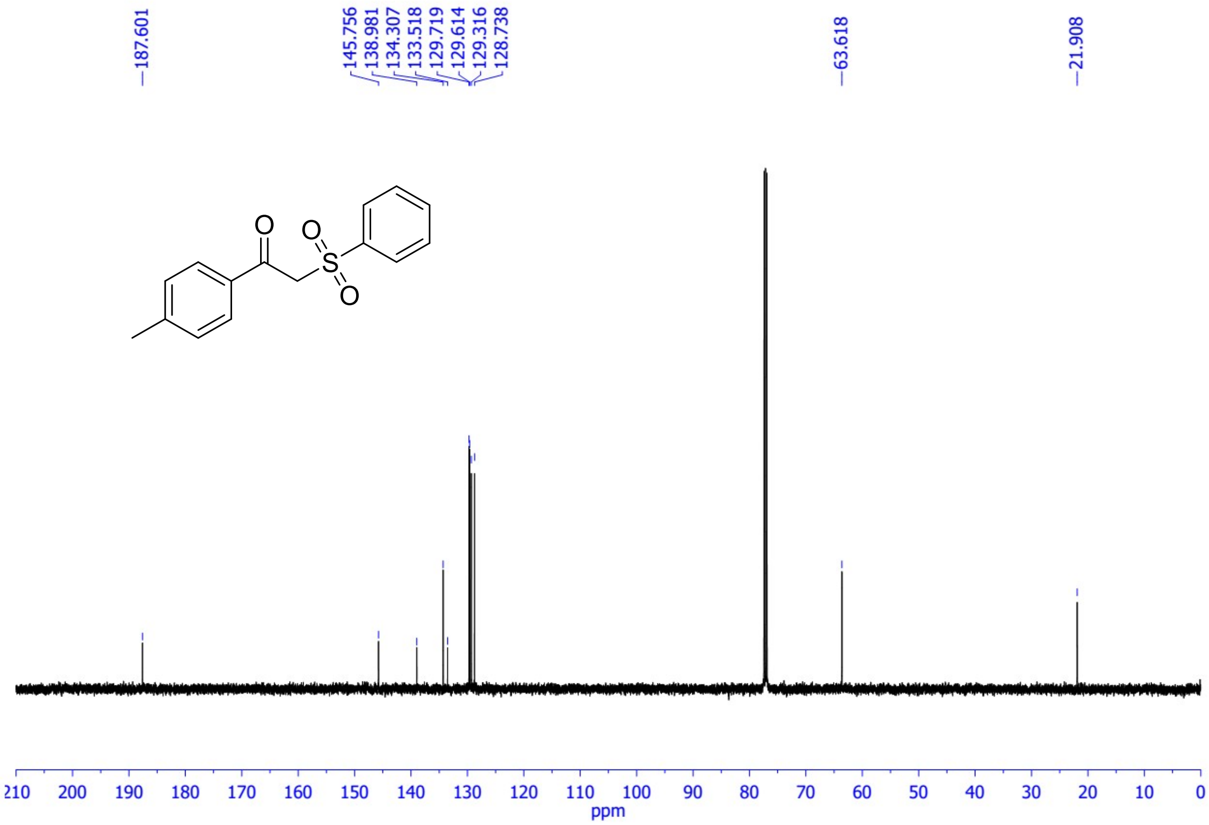

Fig. S13. ^{13}C-NMR spectra of 2-(phenylsulfonyl)-1-(p-tolyl)ethanone

Characterization Data for 2-(phenylsulfonyl)-1-(p-tolyl)ethanone

Prepared as shown in the general experimental procedure and purified on silica gel (hexane/ethyl acetate = 3:1): white solid, 88% yield. ^{1}H NMR (500 MHz, $CDCl_3$) δ 7.90 – 7.89 (m, 1H), 7.89 – 7.87 (m, 1H), 7.68 – 7.64 (m, 1H), 7.56 – 7.52 (m, 2H), 7.27 (d, J = 8.0 Hz, 2H), 4.70 (s, 2H), 2.42 (s, 3H). ^{13}C NMR (125 MHz, $CDCl_3$) δ = 187.60, 145.76, 138.98, 134.31, 133.52, 129.72, 129.61, 129.32, 128.74, 63.62, 21.91.

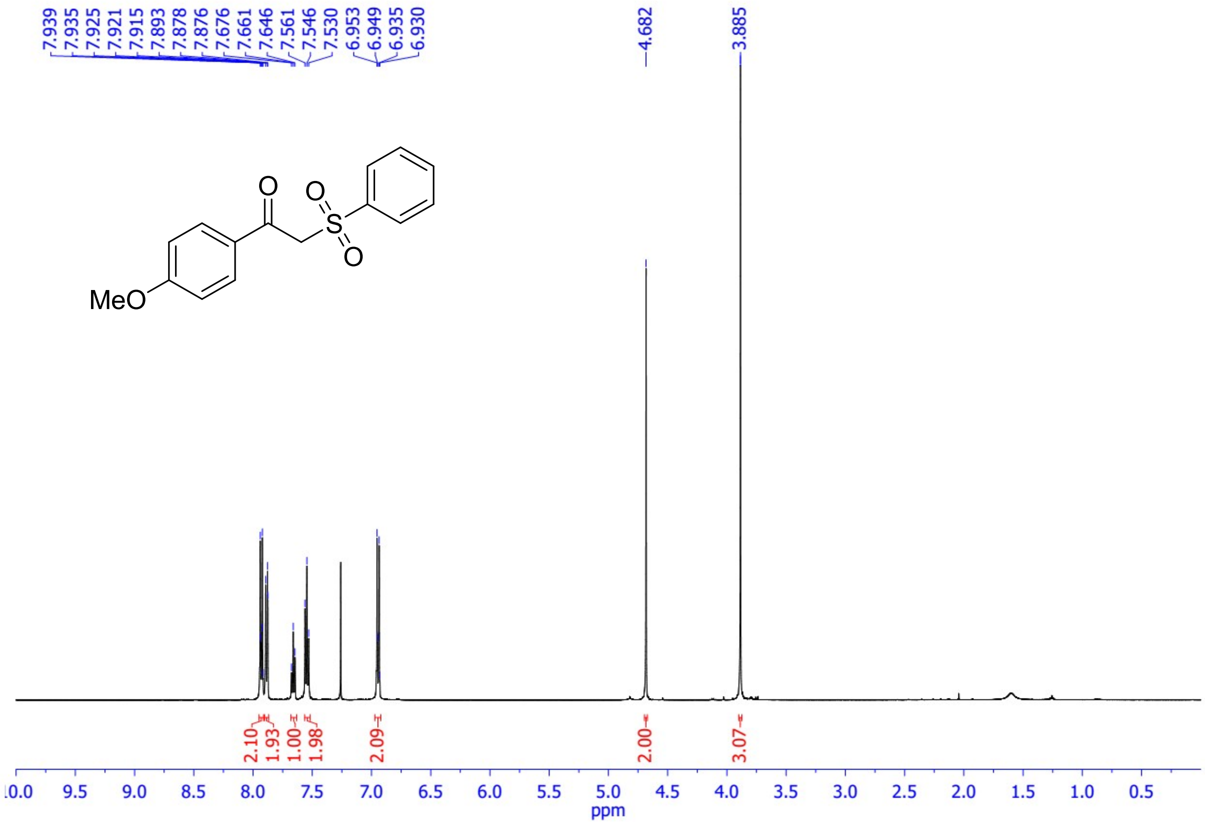

Fig. S14. ^{1}H-NMR spectra of 1-(4-methoxyphenyl)-2-(phenylsulfonyl)ethanone

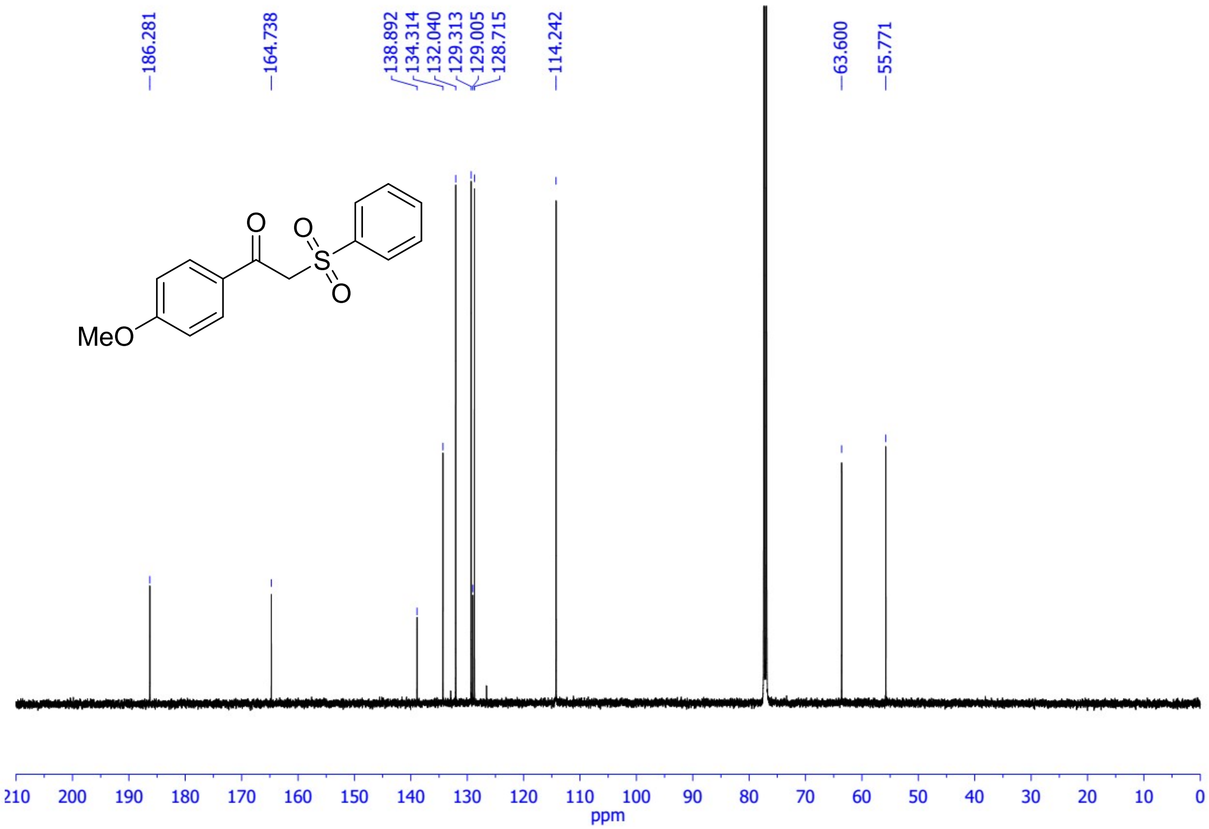

Fig. S15. ^{13}C-NMR spectra of 1-(4-methoxyphenyl)-2-(phenylsulfonyl)ethanone

Characterization Data for 1-(4-methoxyphenyl)-2-(phenylsulfonyl)ethanone

Prepared as shown in the general experimental procedure and purified on silica gel (hexane/ethyl acetate = 3:1): yellow solid, 91% yield. ^{1}H NMR (500 MHz, $CDCl_3$) δ 7.95 – 7.91 (m, 2H), 7.90 – 7.87 (m, 2H), 7.66 (t, J = 7.5 Hz, 1H), 7.55 (t, J = 7.5 Hz, 2H), 6.96 – 6.92 (m, 2H), 4.68 (s, 2H), 3.89 (s, 3H). ^{13}C NMR (125 MHz, $CDCl_3$) δ = 186.3, 164.7, 138.9, 134.3, 132.0, 129.3, 129.0, 128.7, 114.2, 63.6, 55.8.

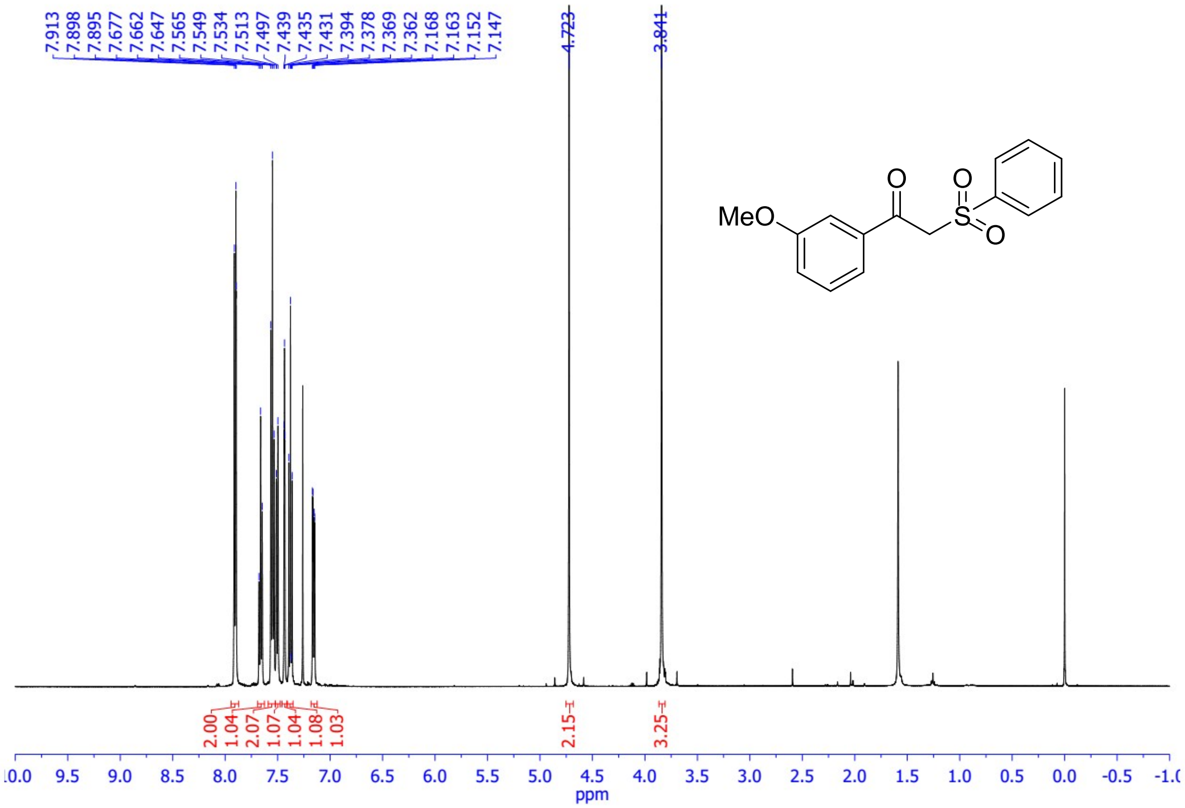

Fig. S16. ^{1}H NMR spectra of 1-(3-methoxyphenyl)-2-(phenylsulfonyl)ethanone

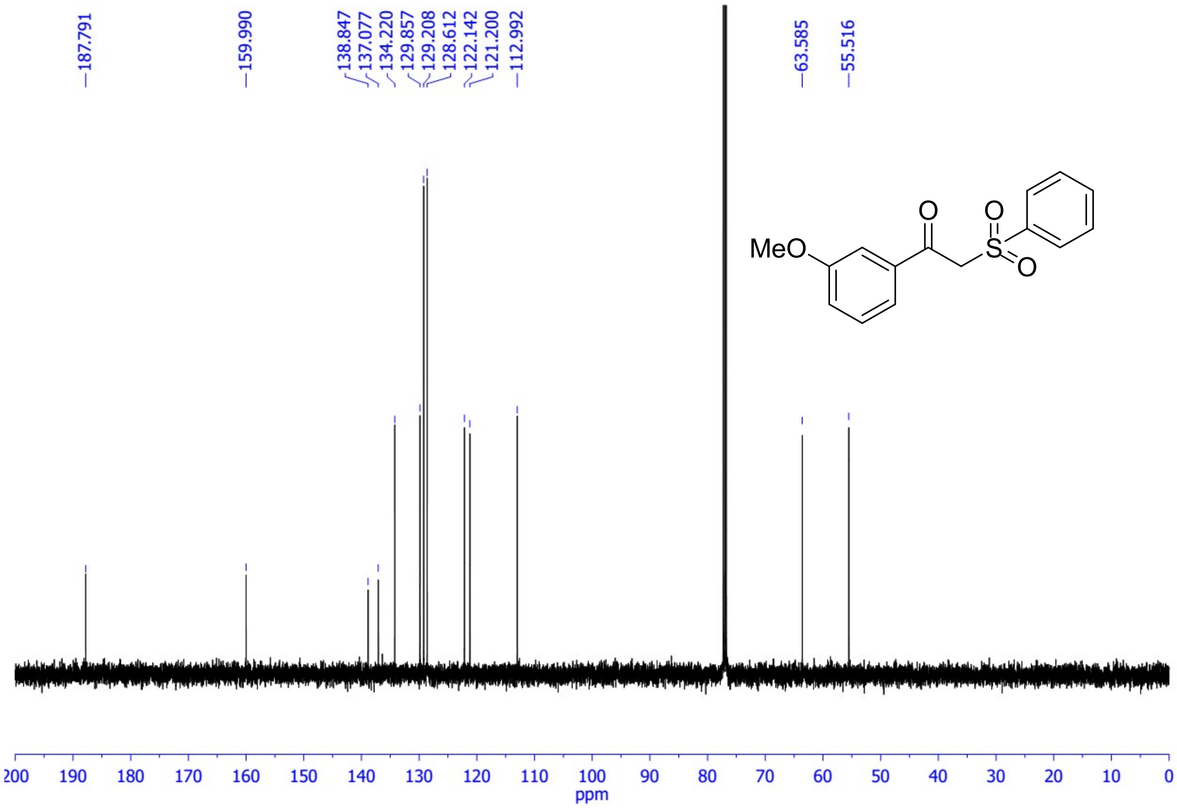

Fig. S17. ^{13}C NMR spectra of 1-(3-methoxyphenyl)-2-(phenylsulfonyl)ethanone

Characterization Data for 1-(3-methoxyphenyl)-2-(phenylsulfonyl)ethanone

Prepared as shown in the general experimental procedure and purified on silica gel (hexane/ethyl acetate = 3:1): white solid, 89% yield. ^{1}H NMR (500 MHz, $CDCl_3$) δ 7.94 – 7.87 (m, 2H), 7.66 (t, J = 7.5 Hz, 1H), 7.55 (t, J = 8 Hz, 2H), 7.50 (d, J = 7.5 Hz, 1H), 7.46 – 7.41 (m, 1H), 7.38 (t, J = 8.0 Hz, 1H), 7.16 (dd, J = 8, 2.5 Hz, 1H), 4.72 (s, 2H), 3.84 (s, 3H). ^{13}C NMR (125 MHz, $CDCl_3$) δ = 187.8, 156.0, 138.8, 137.1, 134.2, 129.9, 129.2, 128.6, 122.1, 121.2, 113.0, 63.6, 55.5.

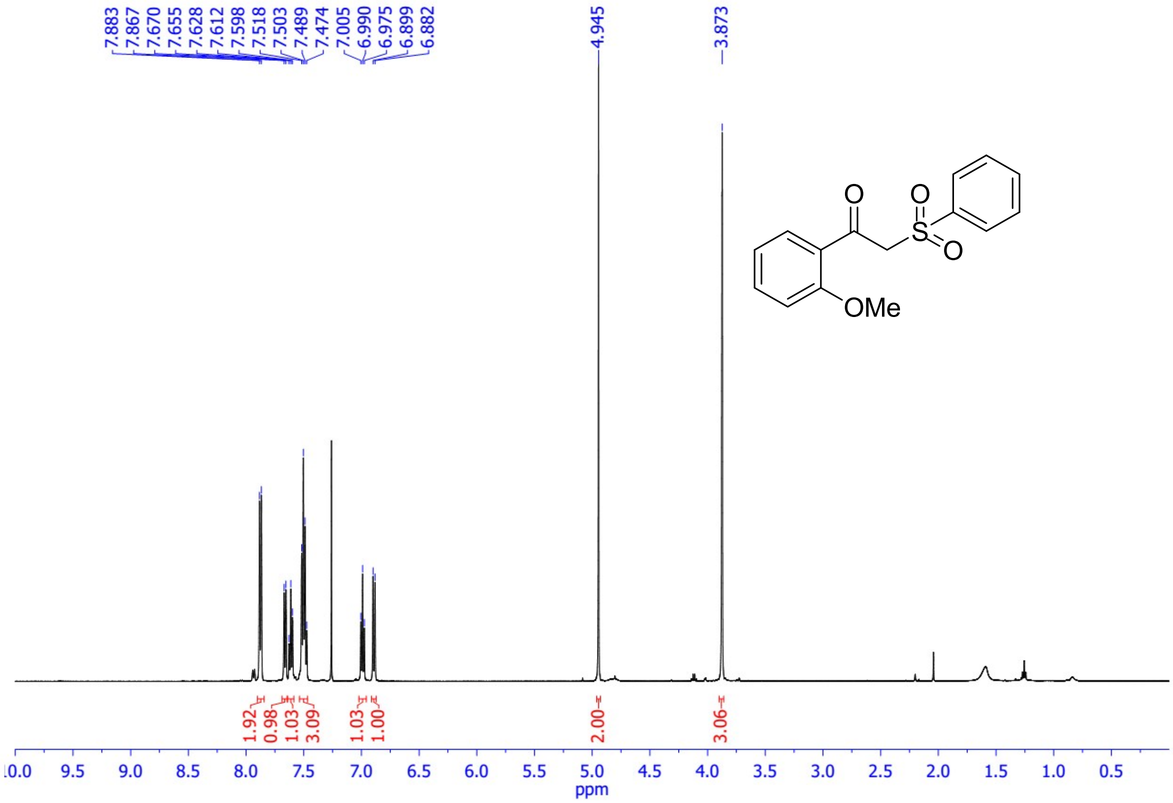

Fig. S18. ^{1}H NMR spectra of 1-(2-methoxyphenyl)-2-(phenylsulfonyl)ethanone

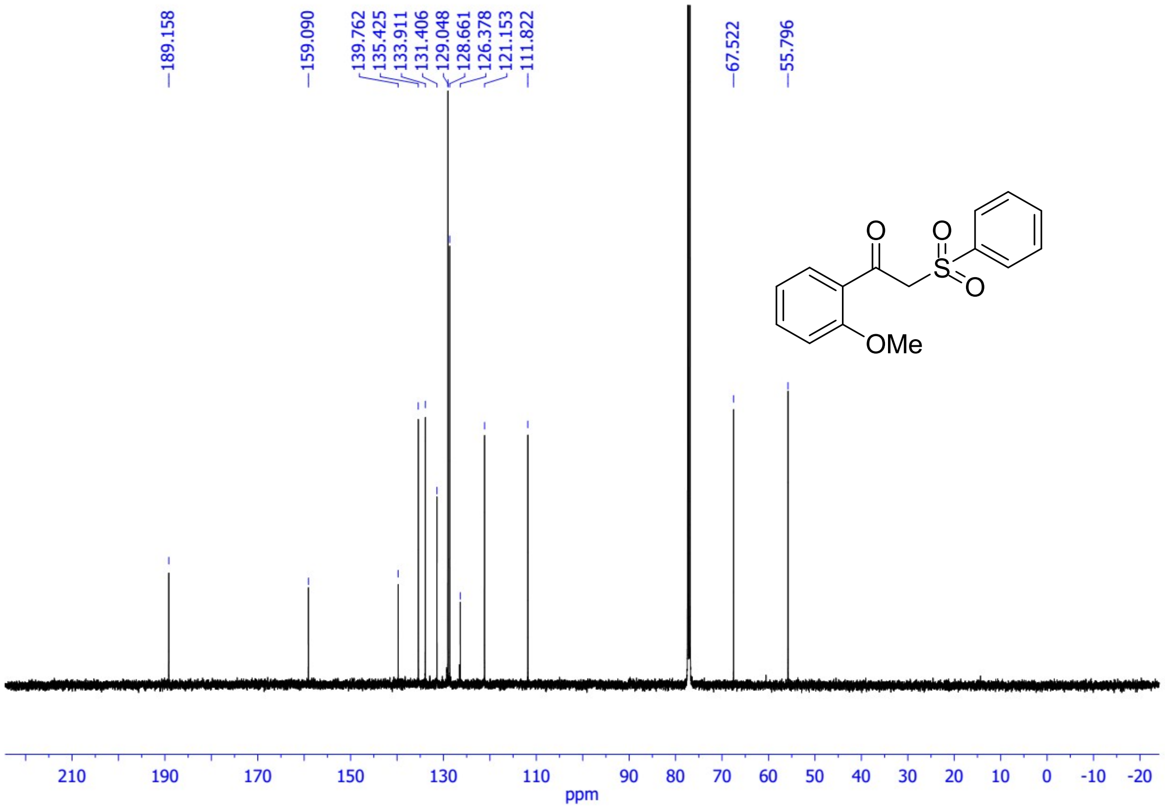

Fig. S19. ^{13}C NMR spectra of 1-(2-methoxyphenyl)-2-(phenylsulfonyl)ethanone

Characterization Data for 1-(2-methoxyphenyl)-2-(phenylsulfonyl)ethanone

Prepared as shown in the general experimental procedure and purified on silica gel (hexane/ethyl acetate = 2:1): yellow solid, 83% yield. ^{1}H NMR (500 MHz, $CDCl_3$) δ 7.88 (d, J = 8.0 Hz, 2H), 7.66 (d, J = 8 Hz, 1H), 7.61 (t, J = 7.5 Hz, 1H), 7.53 – 7.47 (m, 3H), 6.99 (t, J = 7.5 Hz, 1H), 6.89 (d, J = 8.4 Hz, 1H), 4.94 (s, 2H), 3.87 (s, 3H). ^{13}C NMR (125 MHz, $CDCl_3$) δ = 189.2, 159.1, 139.8, 135.4, 133.9, 131.4, 129.0, 128.7, 126.4, 121.2, 111.8, 67.5, 55.8.

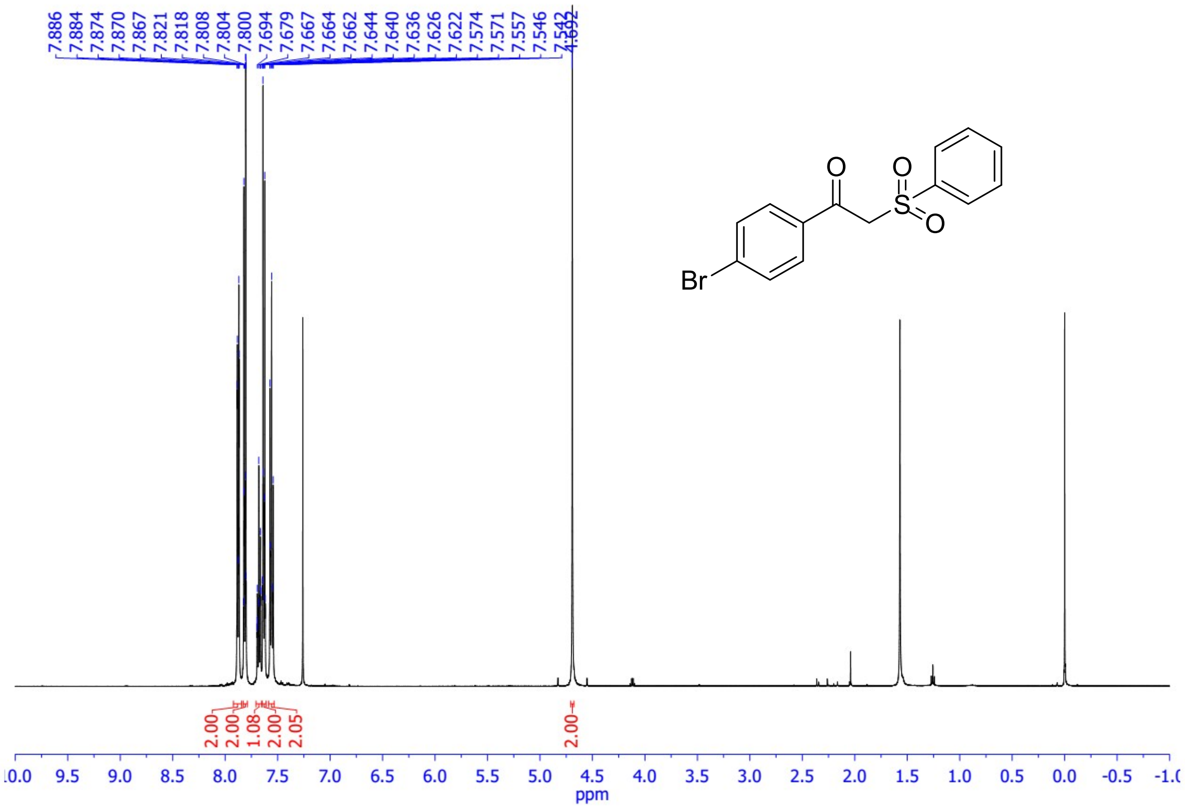

Fig. S20. ^{1}H NMR spectra of 1-(4-bromophenyl)-2-(phenylsulfonyl)ethanone

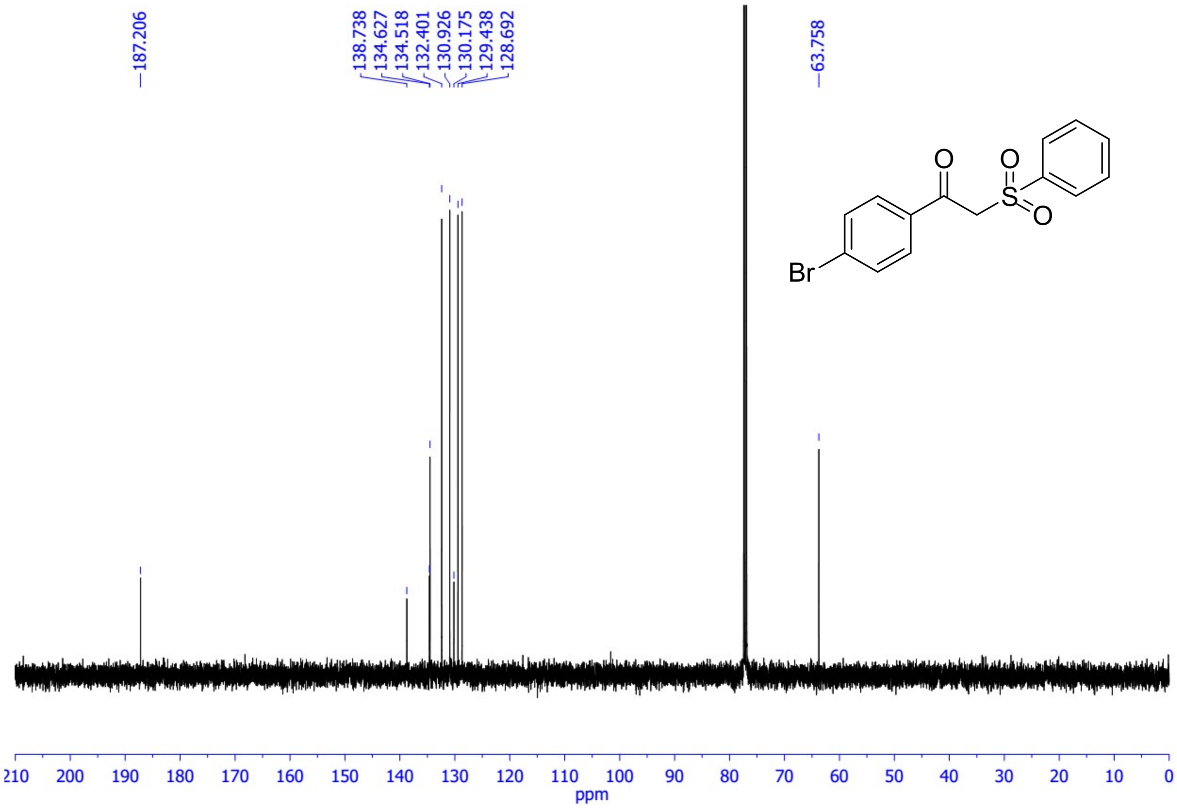

Fig. S21. ^{13}C NMR spectra of 1-(4-bromophenyl)-2-(phenylsulfonyl)ethanone

Characterization Data for 1-(4-bromophenyl)-2-(phenylsulfonyl)ethanone

Prepared as shown in the general experimental procedure and purified on silica gel (hexane/ethyl acetate = 3:1): yellow solid, 76% yield. ^{1}H NMR (500 MHz, $CDCl_3$) δ 7.92 – 7.84 (m, 2H), 7.83 – 7.79 (m, 2H), 7.71 – 7.65 (m, 1H), 7.65 – 7.61 (m, 2H), 7.59 – 7.53 (m, 2H), 4.69 (s, 2H). ^{13}C NMR (125 MHz, $CDCl_3$) δ = 187.2, 138.7, 134.6, 134.5, 132.40, 130.9, 130.2, 129.4, 128.7, 63.7.

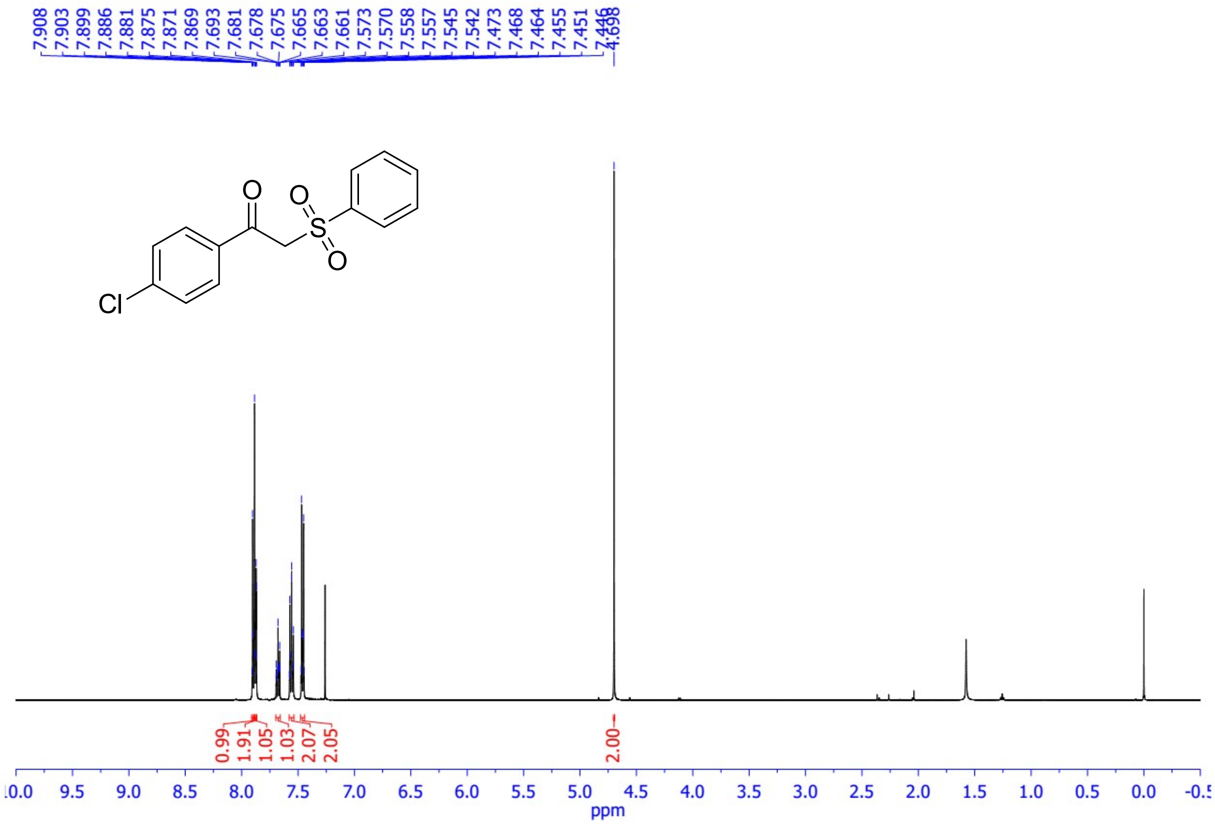

Fig. S22. ^{1}H-NMR spectra of 1-(4-chlorophenyl)-2-(phenylsulfonyl)ethanone

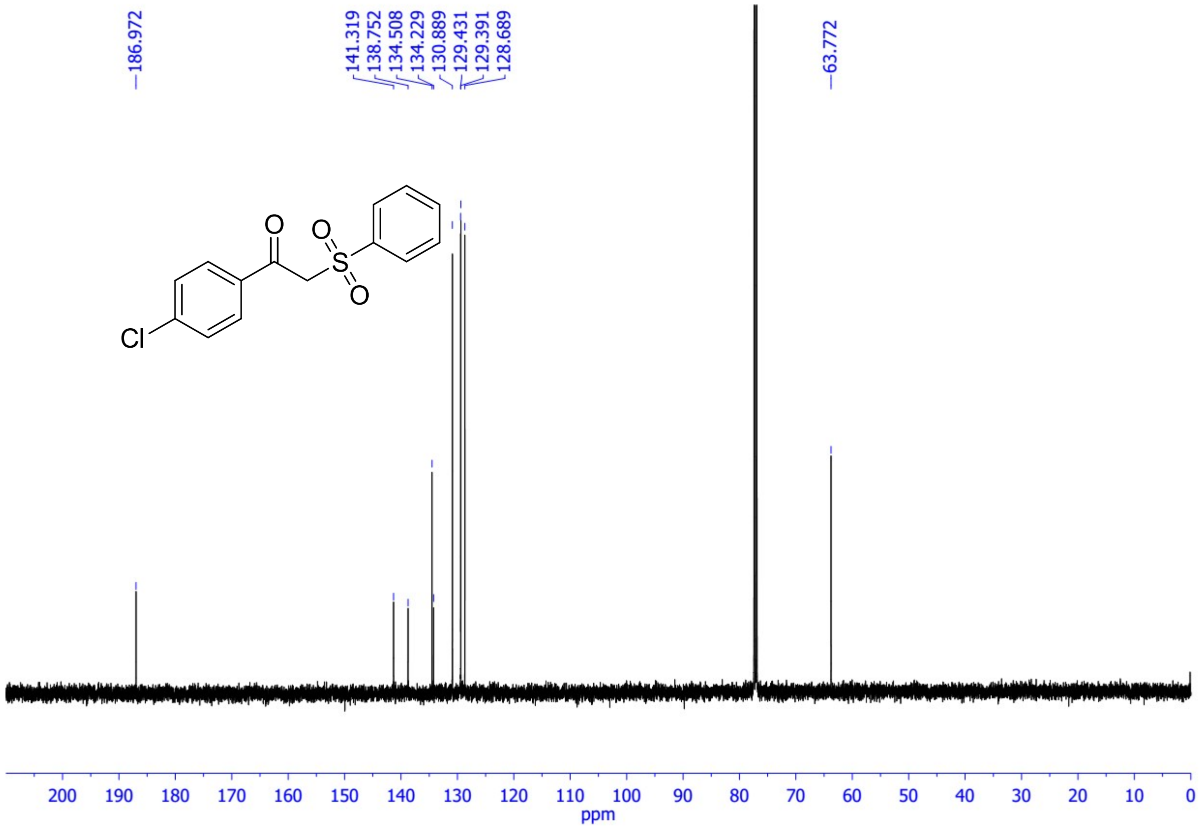

Fig. S23. ^{13}C-NMR spectra of 1-(4-chlorophenyl)-2-(phenylsulfonyl)ethanone

Characterization Data for 1-(4-chlorophenyl)-2-(phenylsulfonyl)ethanone

Prepared as shown in the general experimental procedure and purified on silica gel (hexane/ethyl acetate = 3:1): yellow solid, 80% yield. ^{1}H NMR (500 MHz, $CDCl_3$) δ 7.91 – 7.90 (m, 1H), 7.89 – 7.88 (m, 2H), 7.88 – 7.87 (m, 1H), 7.70 – 7.66 (m, 1H), 7.58 – 7.54 (m, 2H), 7.48 – 7.44 (m, 2H), 4.70 (s, 2H). ^{13}C NMR (126 MHz, $CDCl_3$) δ = 187.0, 141.3, 138.8, 134.5, 134.2, 130.9, 129.4, 129.4, 128.7, 63.8.

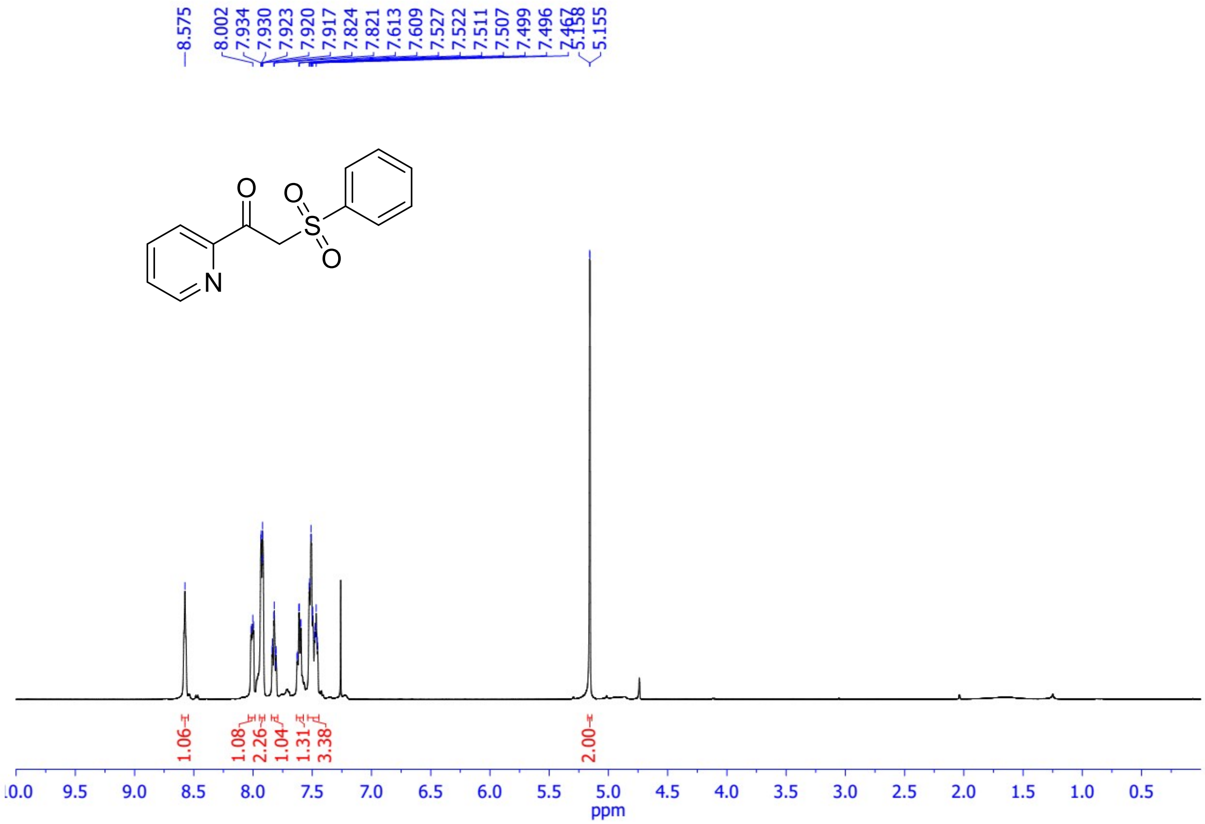

Fig. S24. ^{1}H-NMR spectra of 2-(phenylsulfonyl)-1-(pyridin-2-yl)ethanone

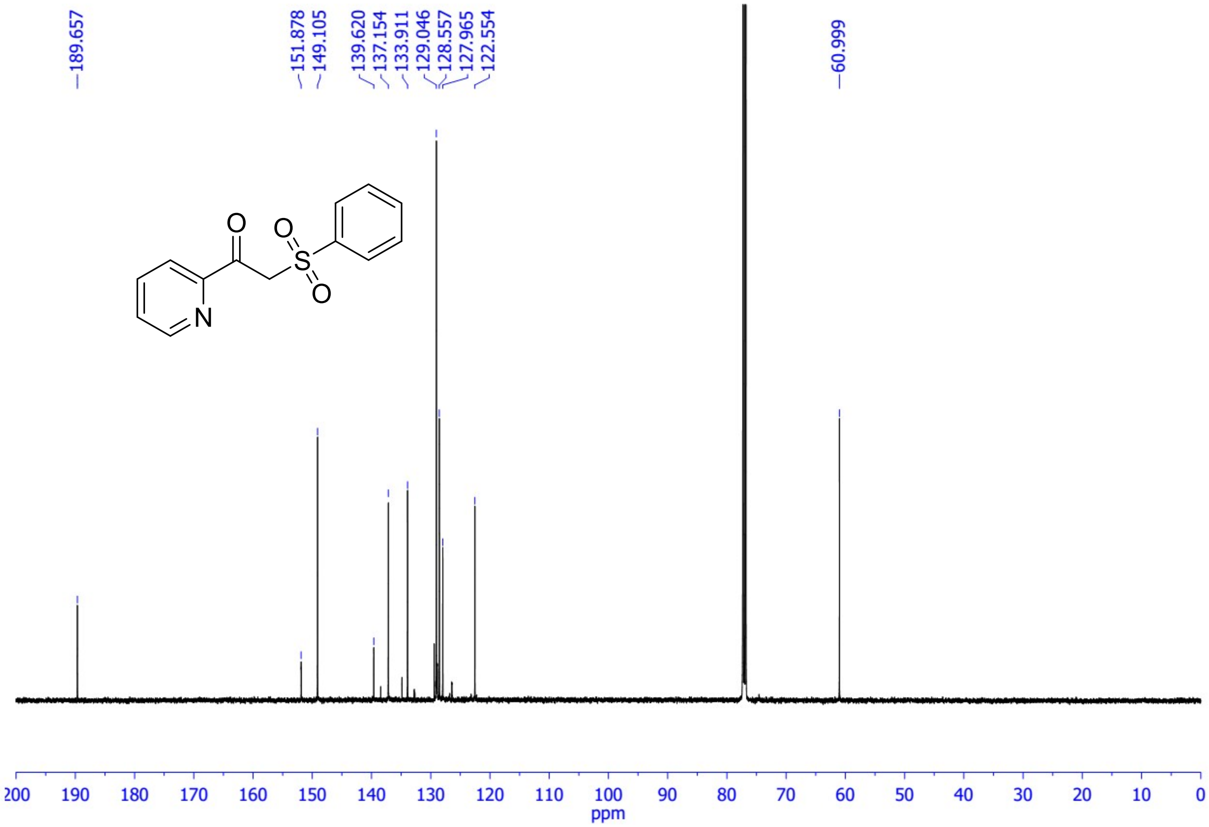

Fig. S25. ^{13}C-NMR spectra of 2-(phenylsulfonyl)-1-(pyridin-2-yl)ethanone

Characterization Data for 2-(phenylsulfonyl)-1-(pyridin-2-yl)ethanone

Prepared as shown in the general experimental procedure and purified on silica gel (hexane/ethyl acetate = 1:1): light brown solid, 80% yield. ^{1}H NMR (500 MHz, $CDCl_3$) δ 8.57 (s, 1H), 8.01 (dd, J = 8, 3 Hz, 1H), 7.95 – 7.90 (m, 2H), 7.85 – 7.79 (m, 1H), 7.63 – 7.57 (m, 1H), 7.54 – 7.44 (m, 3H), 5.16 (d, J = 1.6 Hz, 2H). ^{13}C NMR (125 MHz, $CDCl_3$) δ = 189.7, 151.9, 149.1, 139.62, 137.2, 133.9, 129.0, 128.6, 128.0, 122.6, 61.0.

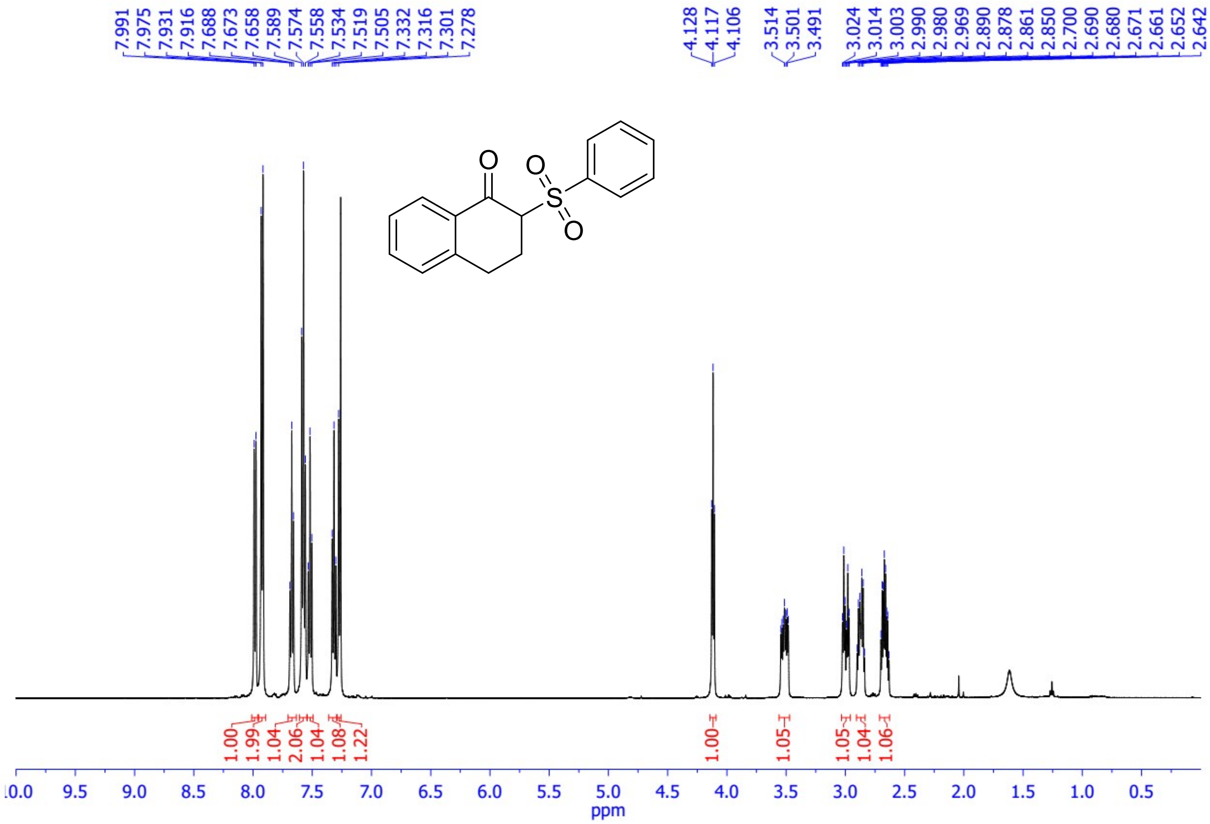

Fig. S26. ^{1}H-NMR spectra of 2-(phenylsulfonyl)-3,4-dihydronaphthalen-1(2H)-one

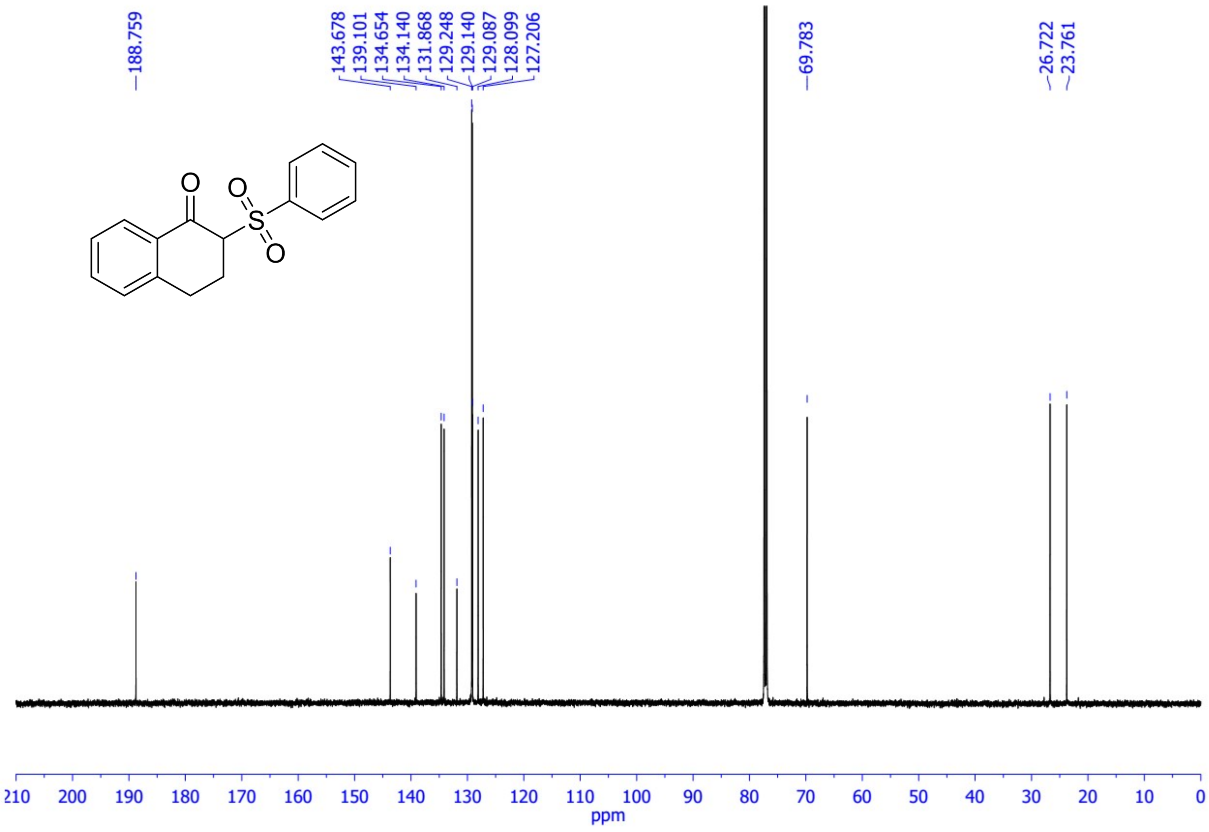

Fig. S27. ^{13}C-NMR spectra of 2-(phenylsulfonyl)-3,4-dihydronaphthalen-1(2H)-one

Characterization Data for 2-(phenylsulfonyl)-3,4-dihydronaphthalen-1(2H)-one

Prepared as shown in the general experimental procedure and purified on silica gel (hexane/ethyl acetate = 3:1): brown solid, 82% yield. ^{1}H NMR (500 MHz, $CDCl_3$) δ 7.98 (d, *J* = 8 Hz, 1H), 7.92 (d, *J* = 7.5 Hz, 2H), 7.67 (t, *J* = 7.5 Hz, 1H), 7.57 (t, *J* = 8 Hz, 2H), 7.52 (t, *J* = 7.5 Hz, 1H), 7.32 (t, *J* = 7.5 Hz, 1H), 7.28 (s, 1H), 4.12 (t, *J* = 5.5 Hz, 1H), 3.56 – 3.47 (m, 1H), 3.04 – 2.96 (m, 1H), 2.91 – 2.83 (m, 1H), 2.71 – 2.63 (m, 1H). ^{13}C NMR (125 MHz, $CDCl_3$) δ = 188.8, 143.7, 139.1, 134.6, 134.1, 131.9, 129.2, 129.1, 129.1, 128.1, 127.2, 69.8, 26.7, 23.8.

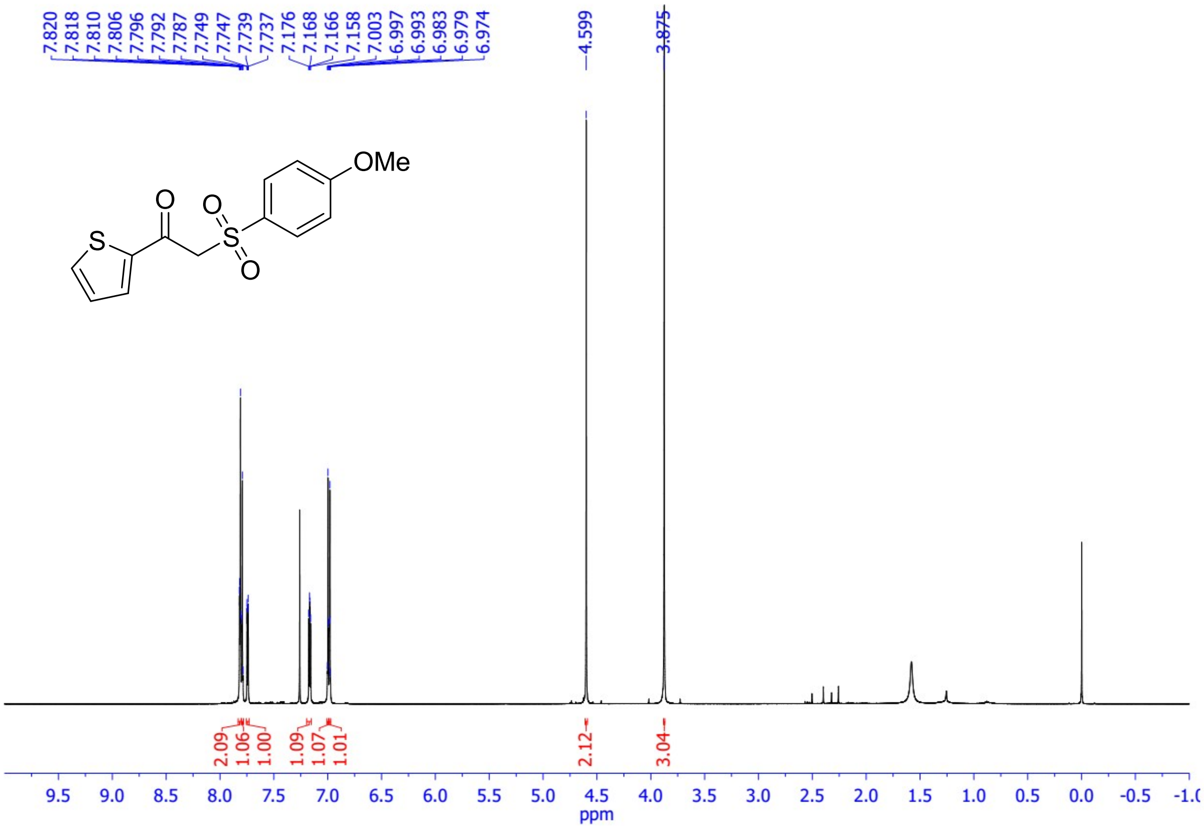

Fig. S28. ^{1}H-NMR spectra of 2-((4-methoxyphenyl)sulfonyl)-1-(thiophen-2-yl)ethanone

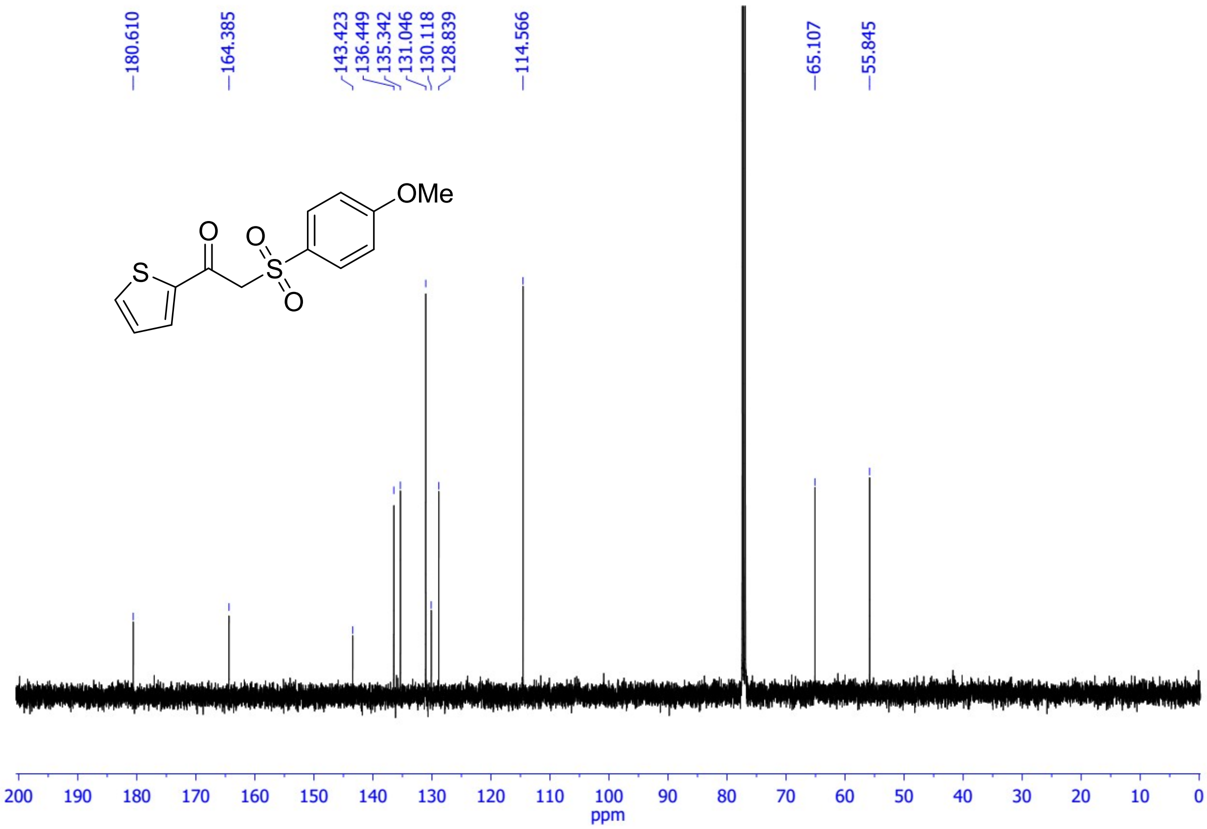

Fig. S29. ^{13}C-NMR spectra of 2-((4-methoxyphenyl)sulfonyl)-1-(thiophen-2-yl)ethanone

Characterization Data for 2-((4-methoxyphenyl)sulfonyl)-1-(thiophen-2-yl)ethanone

Prepared as shown in the general experimental procedure and purified on silica gel (hexane/ethyl acetate = 2:1): yellow solid, 90% yield. ^{1}H NMR (500 MHz, $CDCl_3$) δ 7.83 – 7.80 (m, 2H), 7.80 – 7.78 (m, 1H), 7.74 (dd, J = 5, 1.0 Hz, 1H), 7.17 (dd, J = 5, 4.0 Hz, 1H), 7.01 – 6.99 (m, 1H), 6.99 – 6.97 (m, 1H), 4.60 (s, 2H), 3.88 (s, 3H). ^{13}C NMR (125 MHz, $CDCl_3$) δ = 180.6, 164.4, 143.4, 136.4, 135.3, 131.0, 130.1, 128.8, 114.6, 65.1, 55.8

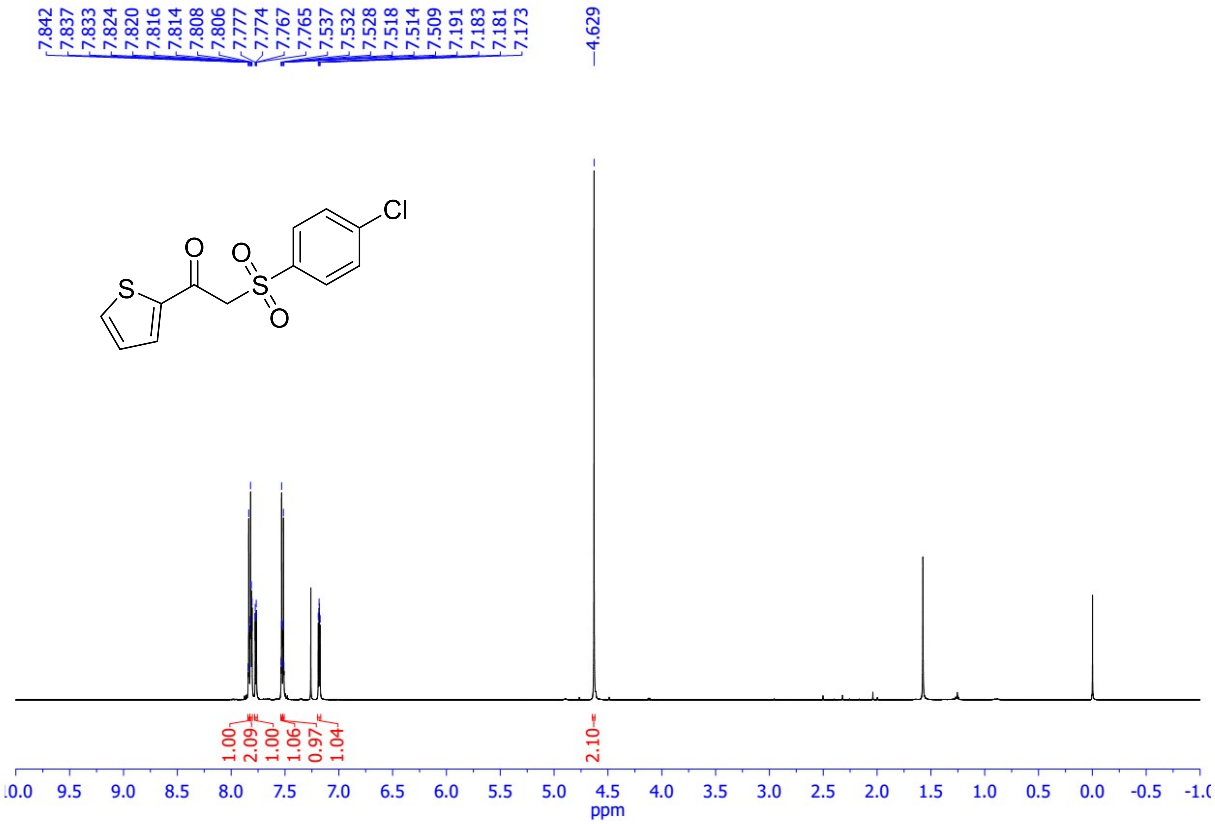

Fig. S30. ^{1}H-NMR spectra of 2-((4-chlorophenyl)sulfonyl)-1-(thiophen-2-yl)ethanone

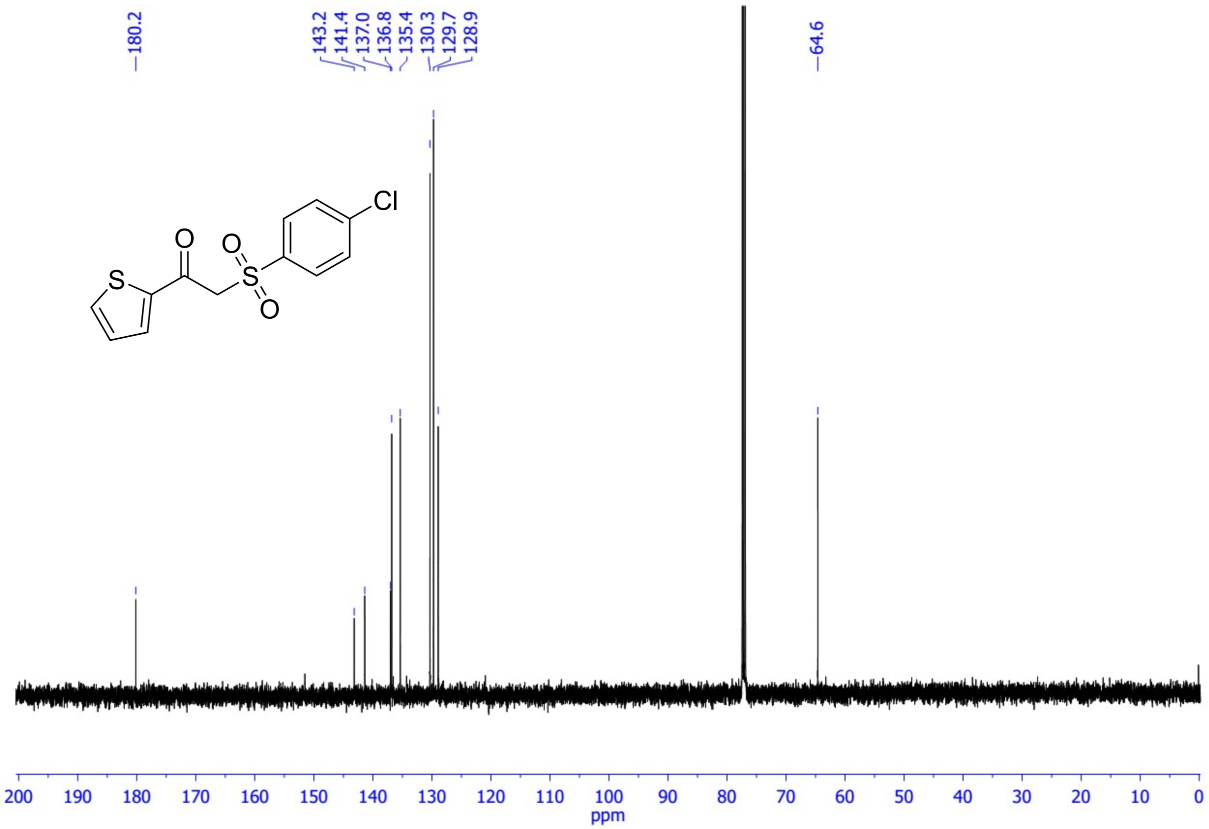

Fig. S31. ^{13}C-NMR spectra of 2-((4-chlorophenyl)sulfonyl)-1-(thiophen-2-yl)ethanone

Characterization Data for 2-((4-chlorophenyl)sulfonyl)-1-(thiophen-2-yl)ethanone

Prepared as shown in the general experimental procedure and purified on silica gel (hexane/ethyl acetate = 2:1): yellow solid, 78% yield. ^{1}H NMR (500 MHz, $CDCl_3$) δ 7.85 – 7.83 (m, 1H), 7.83 – 7.80 (m, 2H), 7.77 (dd, J = 5, 1 Hz, 1H), 7.54 – 7.52 (m, 1H), 7.52 – 7.51 (m, 1H), 7.18 (dd, J = 5, 4 Hz, 1H), 4.63 (s, 2H). ^{13}C NMR (125 MHz, $CDCl_3$) δ = 180.2, 143.2, 141.4, 137.0, 136.8, 135.4, 130.3, 129.7, 128.9, 64.6.

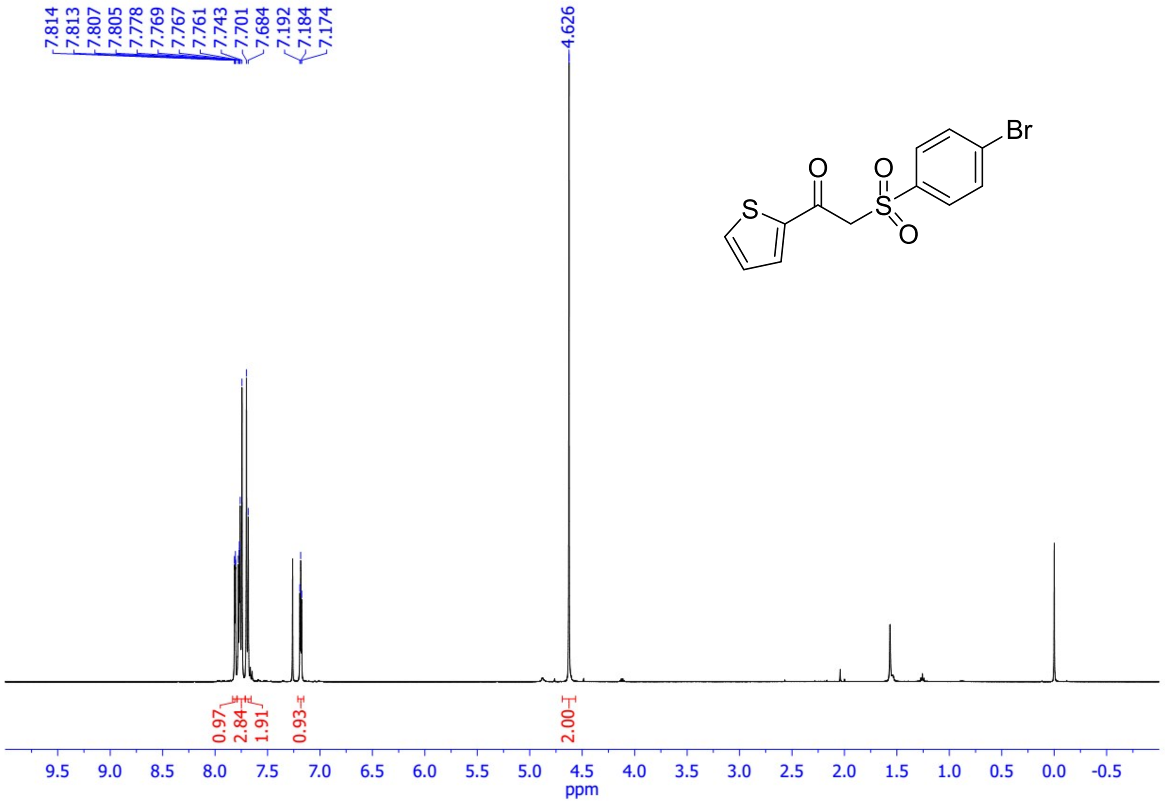

Fig. S32. ^{1}H NMR spectra of 2-((4-bromophenyl)sulfonyl)-1-(thiophen-2-yl)ethanone

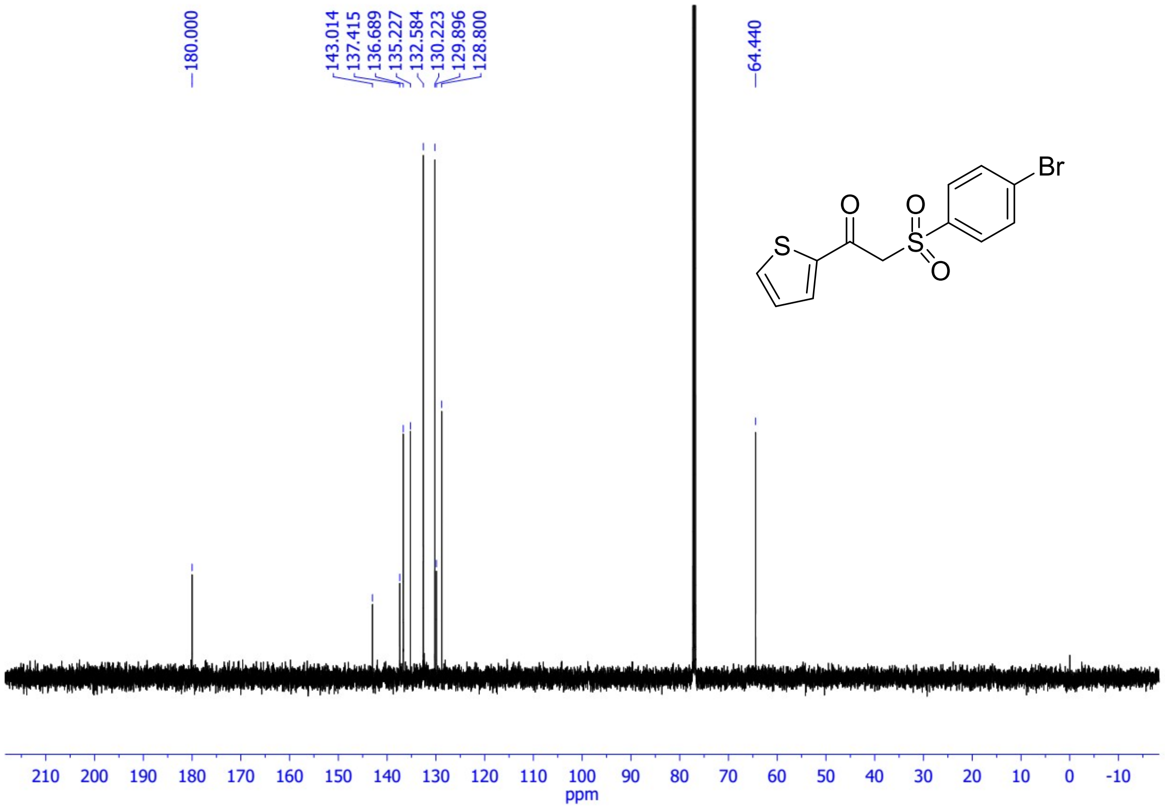

Fig. S33. ^{13}C NMR spectra of 2-((4-bromophenyl)sulfonyl)-1-(thiophen-2-yl)ethanone

Characterization Data for 2-((4-bromophenyl)sulfonyl)-1-(thiophen-2-yl)ethanone

Prepared as shown in the general experimental procedure and purified on silica gel (hexane/ethyl acetate = 2:1): yellow solid, 65% yield. ^{1}H NMR (500 MHz, $CDCl_3$) δ 7.81 (dd, J = 4.0, 1 Hz, 1H), 7.79 – 7.71 (m, 3H), 7.69 (d, J = 8.5 Hz, 2H), 7.21 – 7.16 (m, 1H), 4.63 (s, 2H). ^{13}C NMR (125 MHz, $CDCl_3$) δ = 180.0, 143.0, 137.4, 136.7, 135.2, 132.6, 130.2, 129.9, 128.8, 64.4.

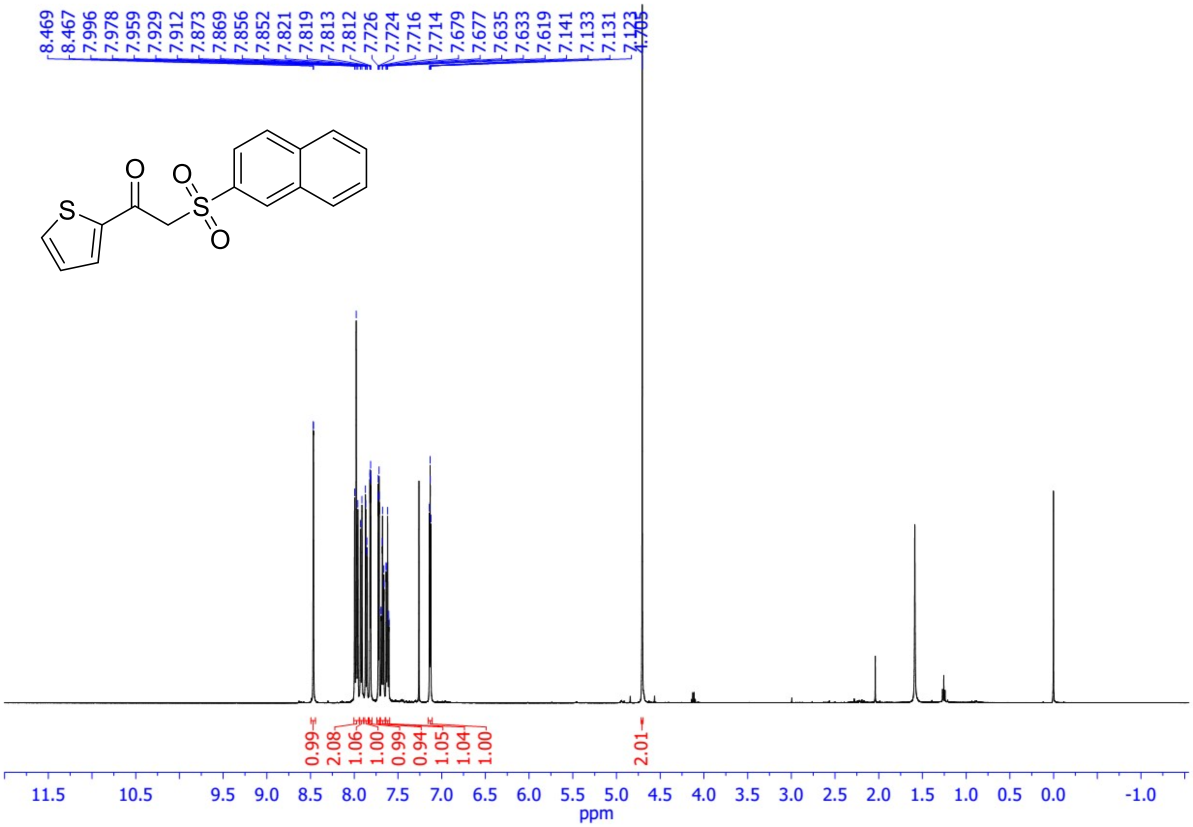

Fig. S34. ^{1}H-NMR spectra of 2-(naphthalen-2-ylsulfonyl)-1-(thiophen-2-yl)ethanone

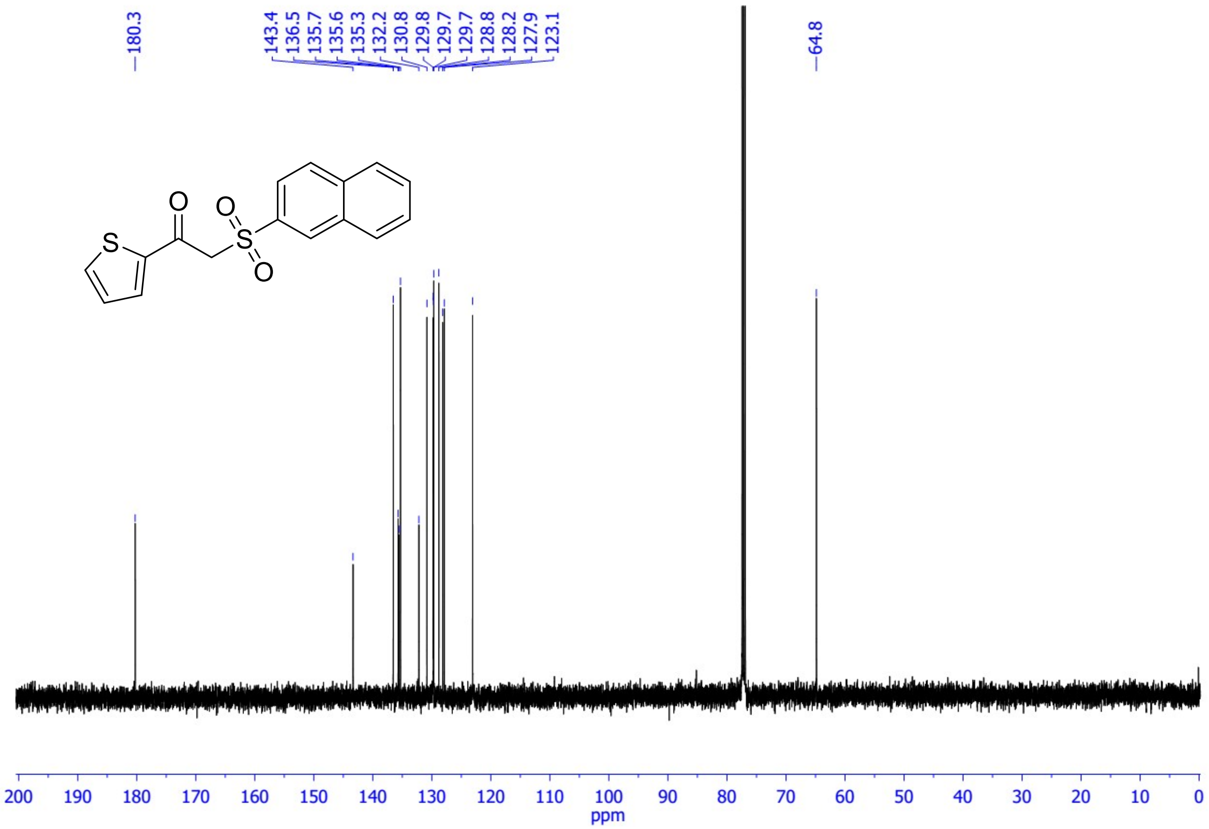

Fig. S35. ^{13}C-NMR spectra of 2-(naphthalen-2-ylsulfonyl)-1-(thiophen-2-yl)ethanone

Characterization Data for 2-(naphthalen-2-ylsulfonyl)-1-(thiophen-2-yl)ethanone

Prepared as shown in the general experimental procedure and purified on silica gel (hexane/ethyl acetate = 2:1): brown solid, 74% yield. ^{1}H NMR (500 MHz, $CDCl_3$) δ 8.47 (d, J = 1 Hz, 1H), 7.98 (t, J = 9 Hz, 2H), 7.92 (d, J = 8 Hz, 1H), 7.86 (dd, J = 8.5, 2 Hz, 1H), 7.82 (dd, J = 4, 1.0 Hz, 1H), 7.72 (dd, J = 5, 1 Hz, 1H), 7.70 – 7.65 (m, 1H), 7.64 – 7.60 (m, 1H), 7.13 (dd, J = 5, 4.0 Hz, 1H), 4.71 (s, 2H). ^{13}C NMR (125 MHz, $CDCl_3$) δ = 180.3, 143.4, 136.5, 135.7, 135.6, 135.3, 132.2, 130.8, 129.8, 129.7, 129.7, 128.8, 128.1, 127.9, 123.1, 64.8.

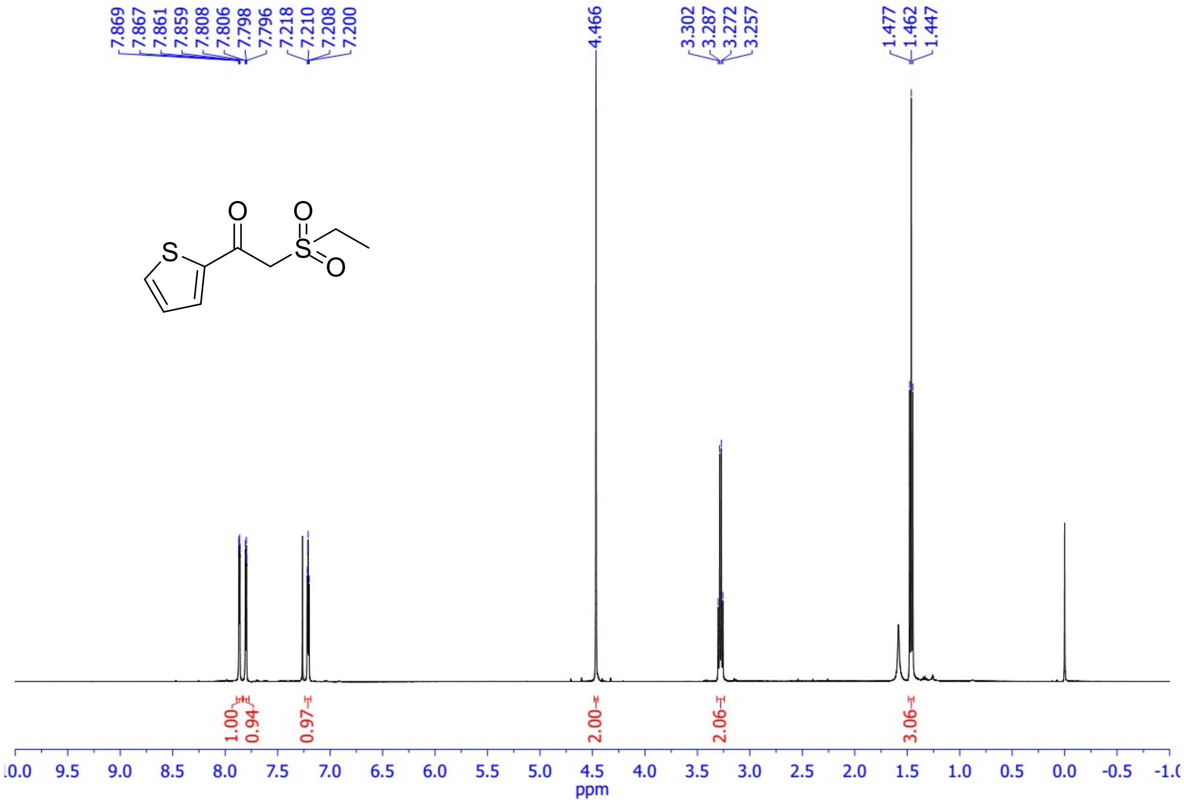

Fig. S36. ^{1}H NMR spectra of 2-(ethylsulfonyl)-1-(thiophen-2-yl)ethanone

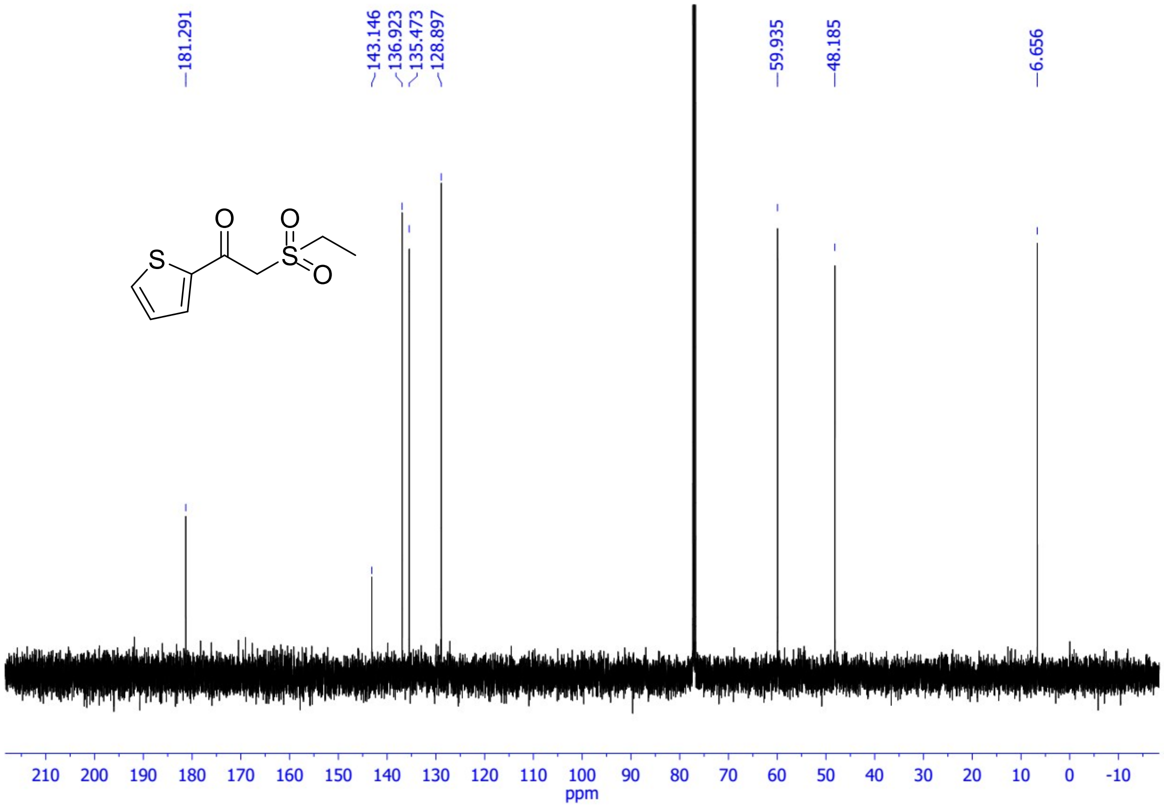

Fig. S37. ^{13}C NMR spectra of 2-(ethylsulfonyl)-1-(thiophen-2-yl)ethanone

Characterization Data for 2-(ethylsulfonyl)-1-(thiophen-2-yl)ethanone

Prepared as shown in the general experimental procedure and purified on silica gel (hexane/ethyl acetate = 2:1): white solid, 79% yield. ^{1}H NMR (500 MHz, $CDCl_3$) δ 7.86 (dd, *J* = 4.0, 1.0 Hz, 1H), 7.80 (dd, *J* = 5.0, 1.0 Hz, 1H), 7.21 (dd, *J* = 5.0, 4.0 Hz, 1H), 4.47 (s, 2H), 3.28 (q, *J* = 7.5 Hz, 2H), 1.46 (t, *J* = 7.5 Hz, 3H). ^{13}C NMR (126 MHz, $CDCl_3$) δ = 181.3, 143.2, 136.9, 135.5, 128.9, 59.9, 48.2, 6.7.

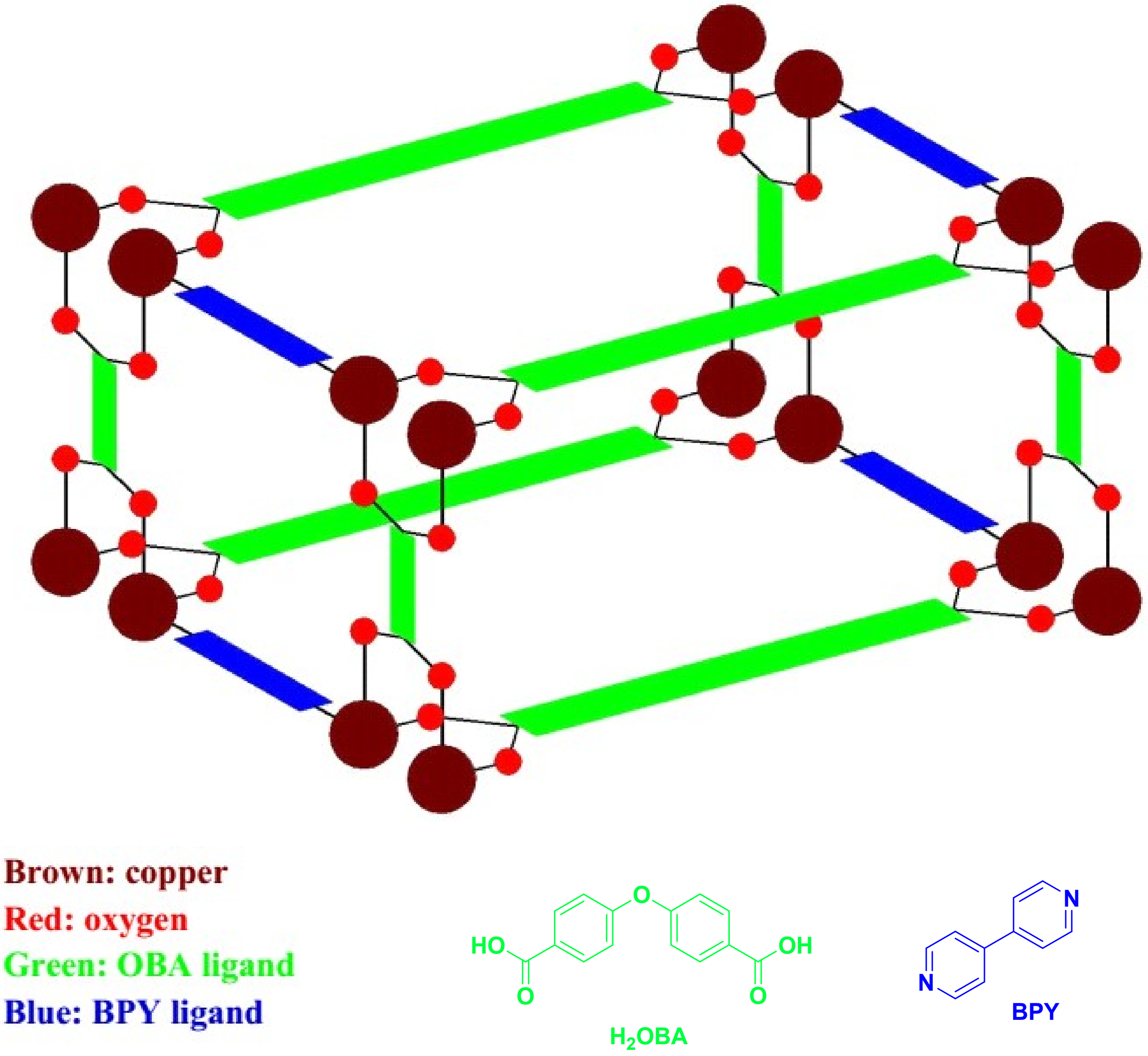

Fig. S38. An illustration of $Cu_2(OBA)_2(BPY)$ structure, showing the arrangement of OBA and BPY ligands in the framework.
